# Supplementary material for: Catalytic condensation for the formation of polycyclic heteroaromatic compounds
Source: Nat Commun. 2018 May 1;9:1751. doi: 10.1038/s41467-018-04143-6 (PMC5931520; doi:10.1038/s41467-018-04143-6)
Supplement: Supplementary file 1 — Supplementary Information [file 41467_2018_4143_MOESM1_ESM.pdf]

Catalytic Condensation for the Formation of Polycyclic Heteroaromatic Compounds

Forberg et al.

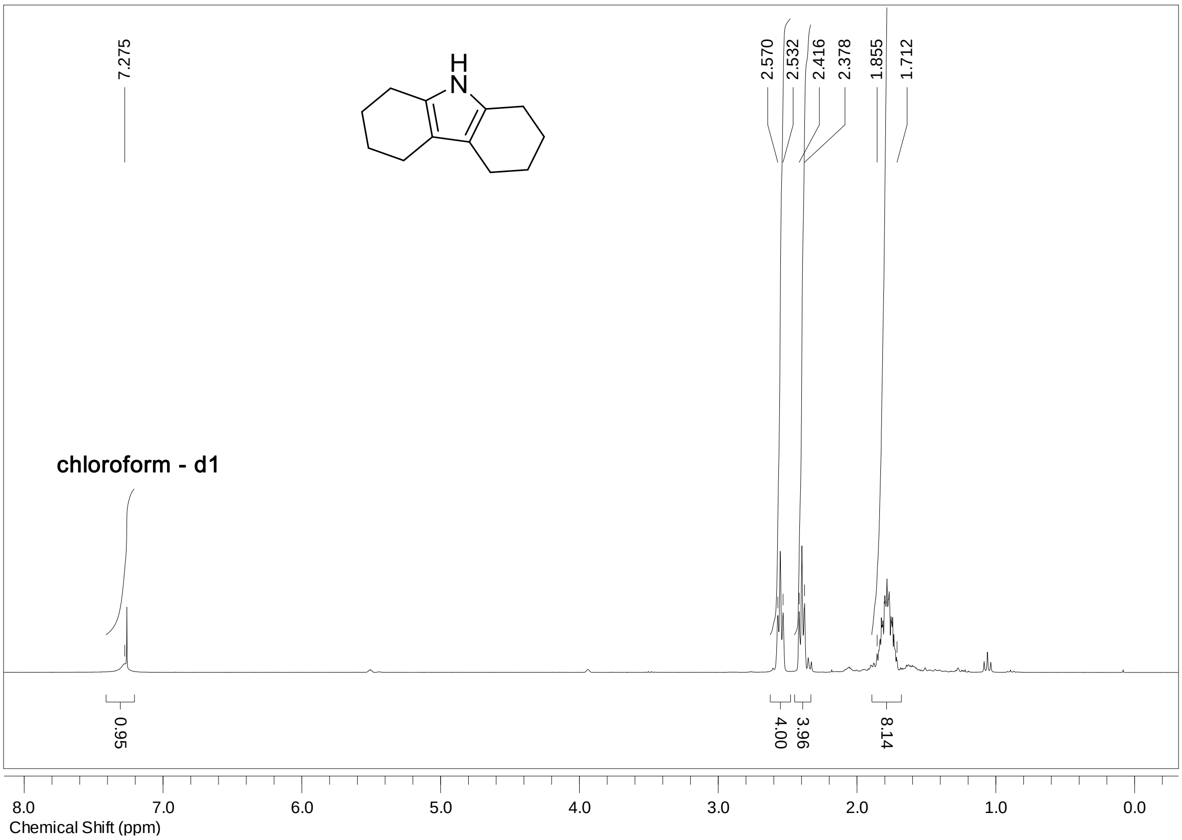

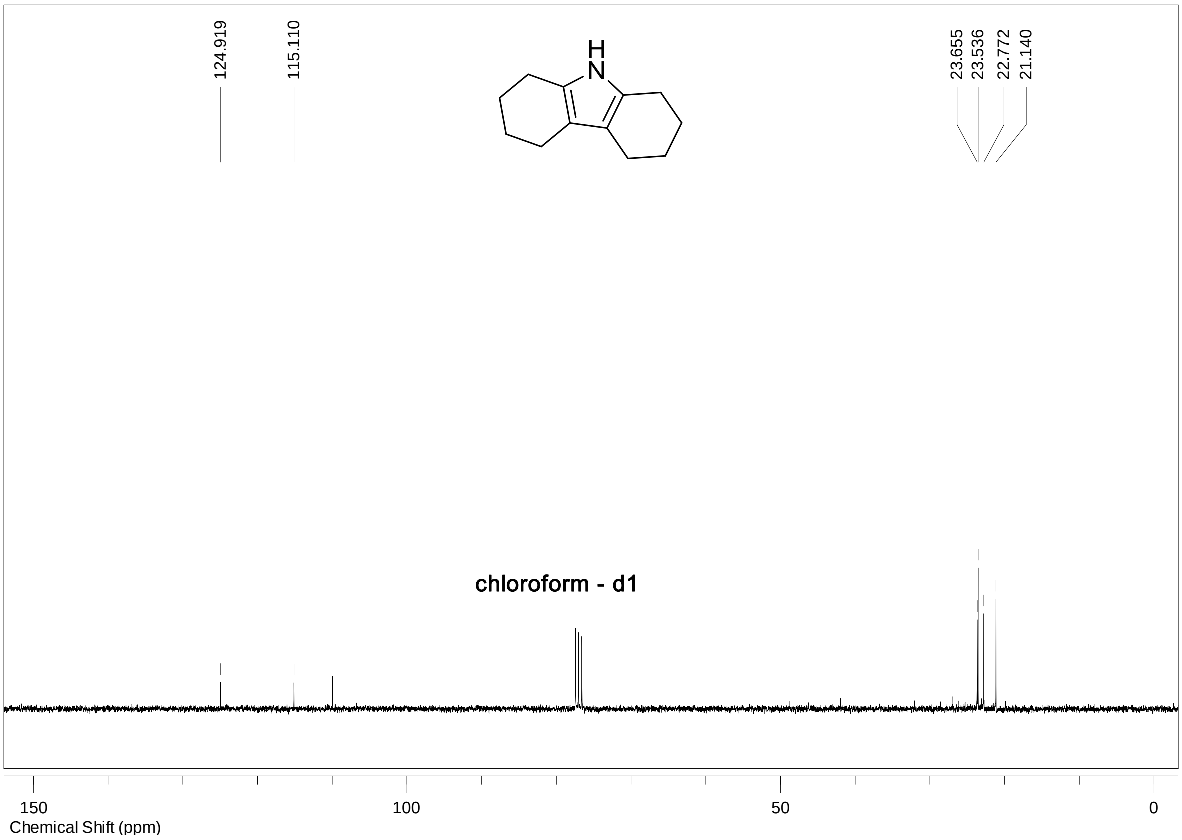

Supplementary Figure 1: NMR Spectra of compound 1a.

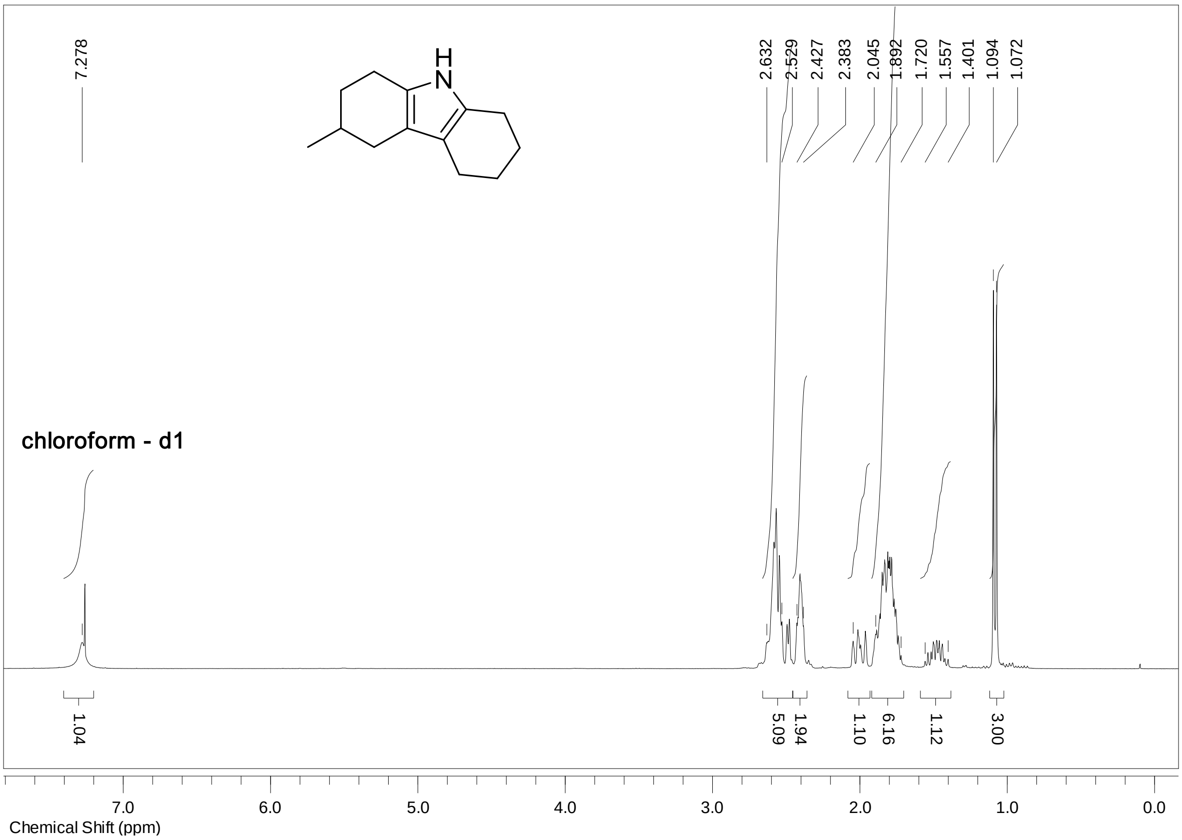

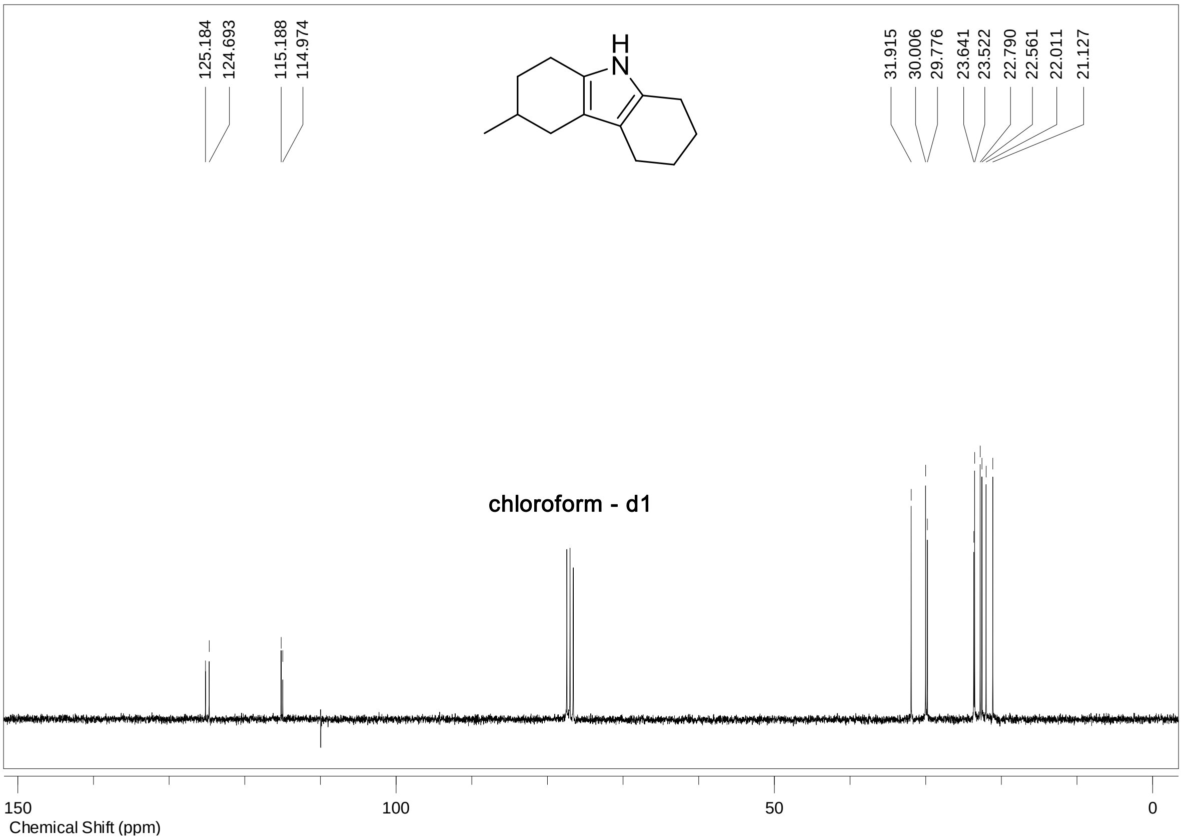

Supplementary Figure 2: NMR Spectra of compound 1b.

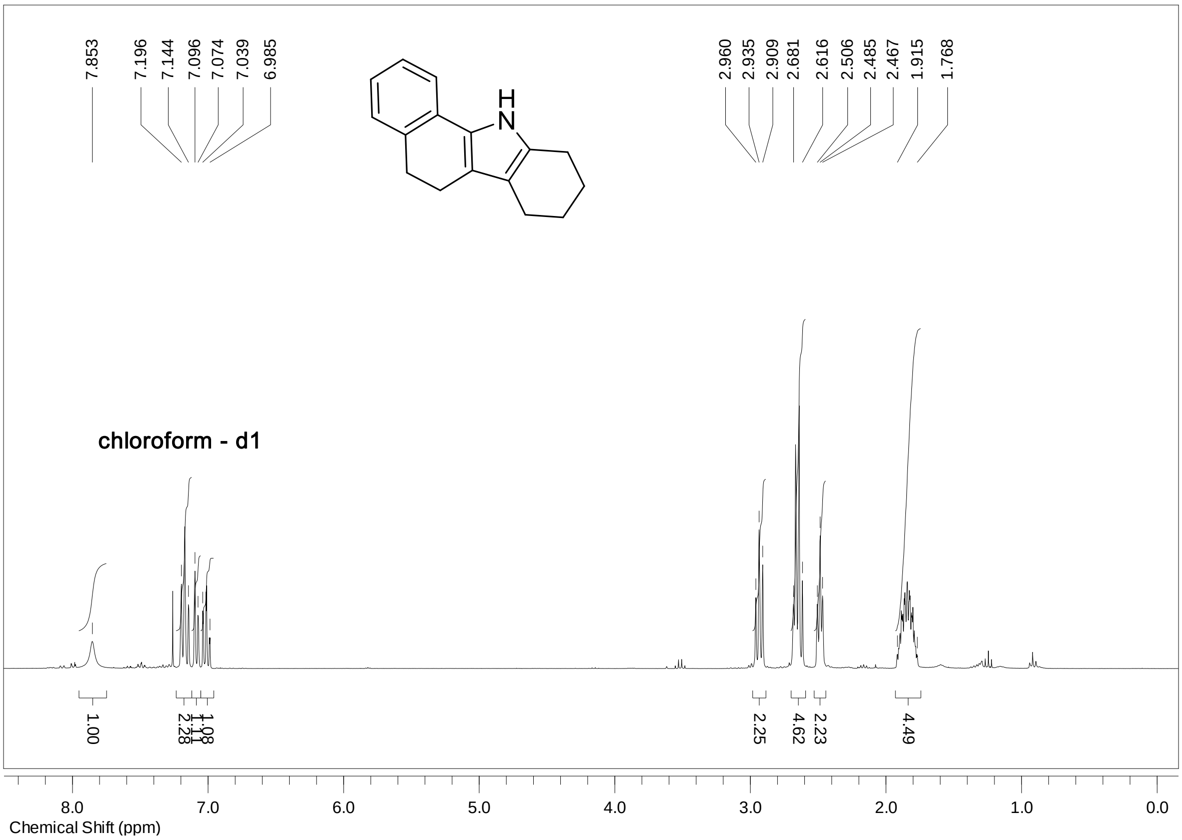

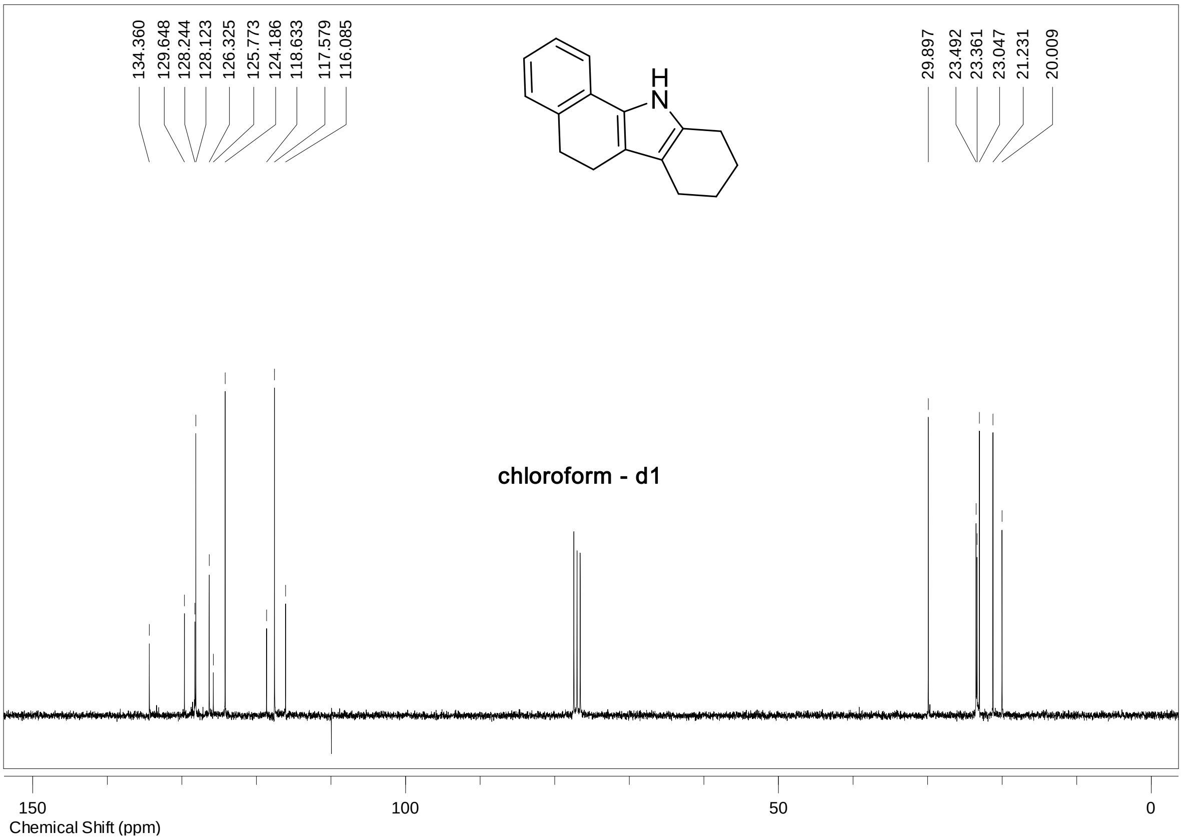

Supplementary Figure 3: NMR Spectra of compound 1c.

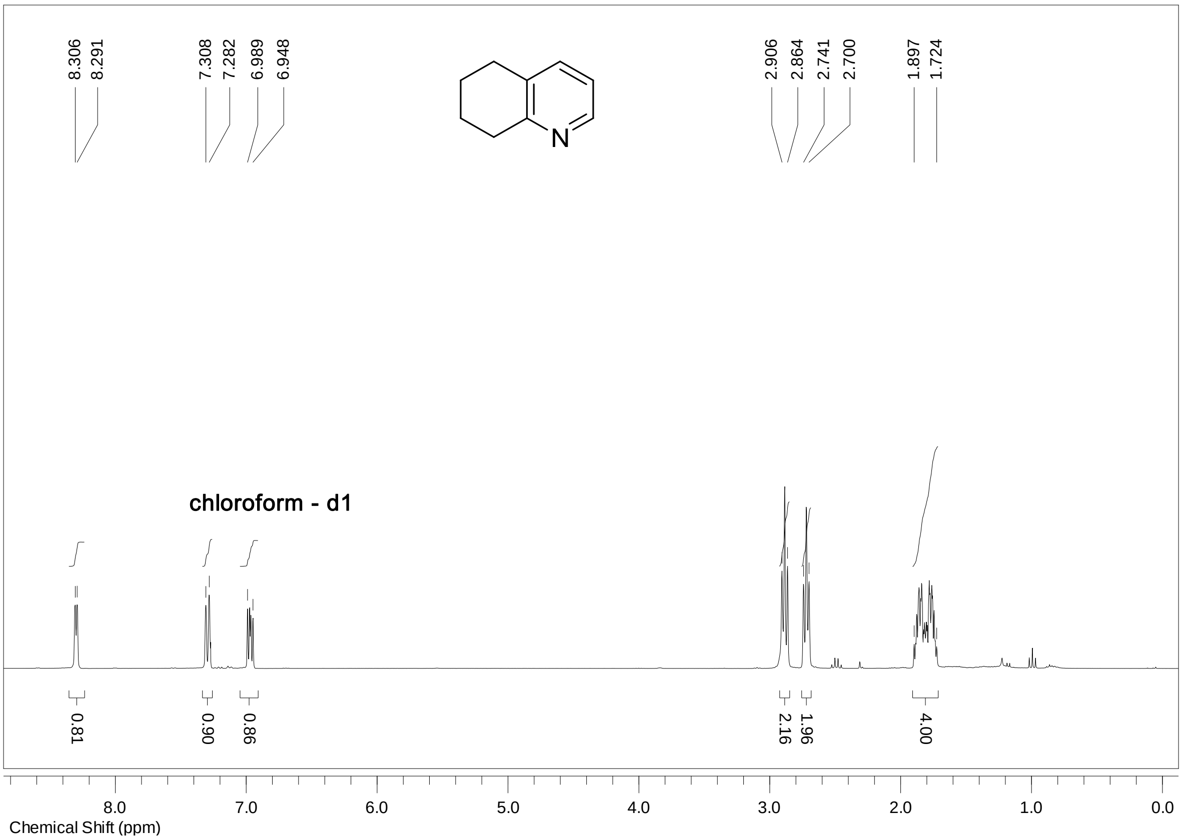

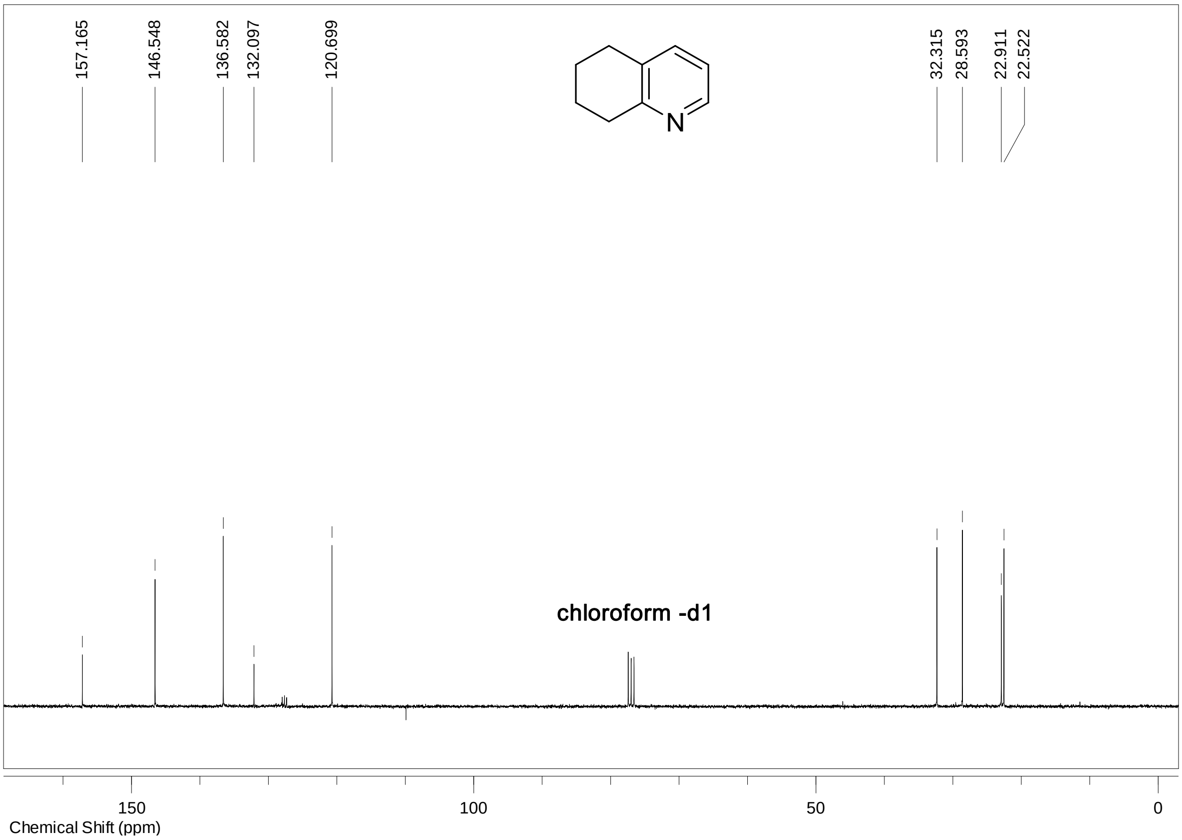

Supplementary Figure 4: NMR Spectra of compound 2a.

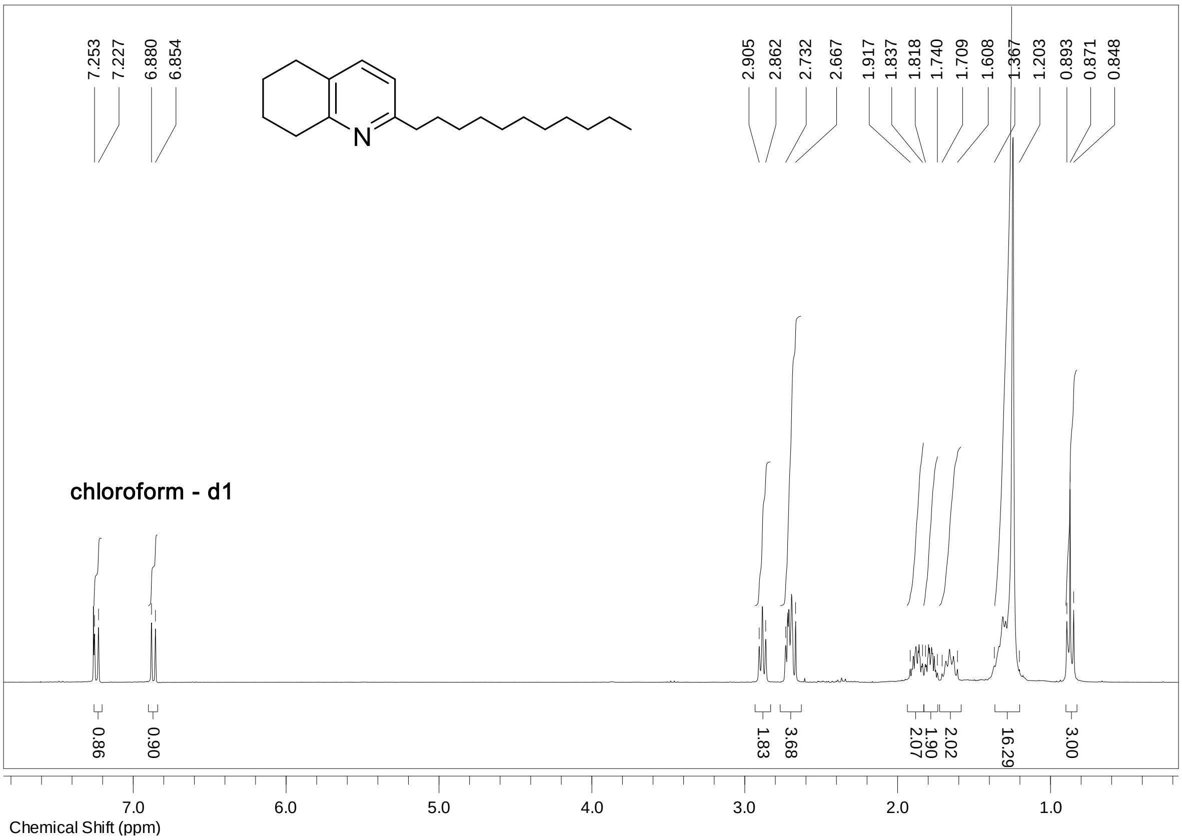

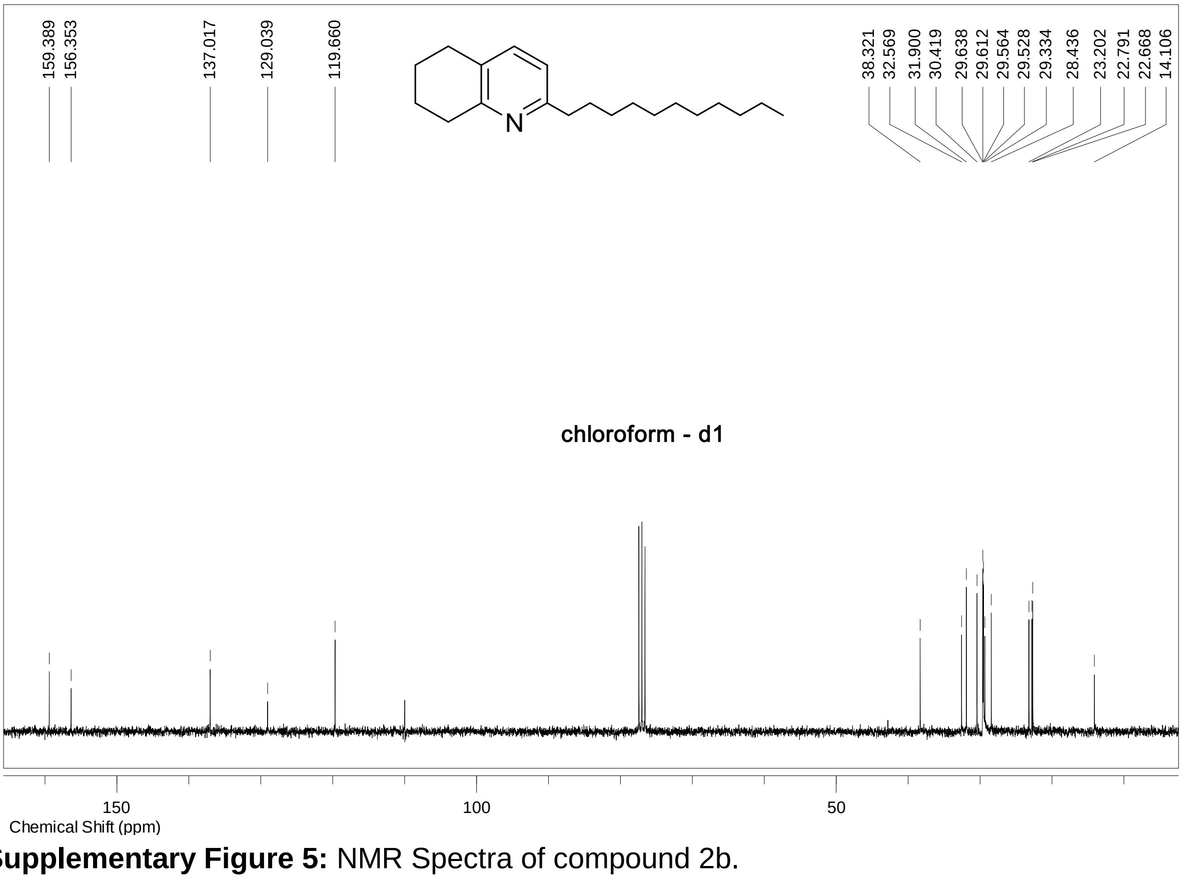

Supplementary Figure 5: NMR Spectra of compound 2b.

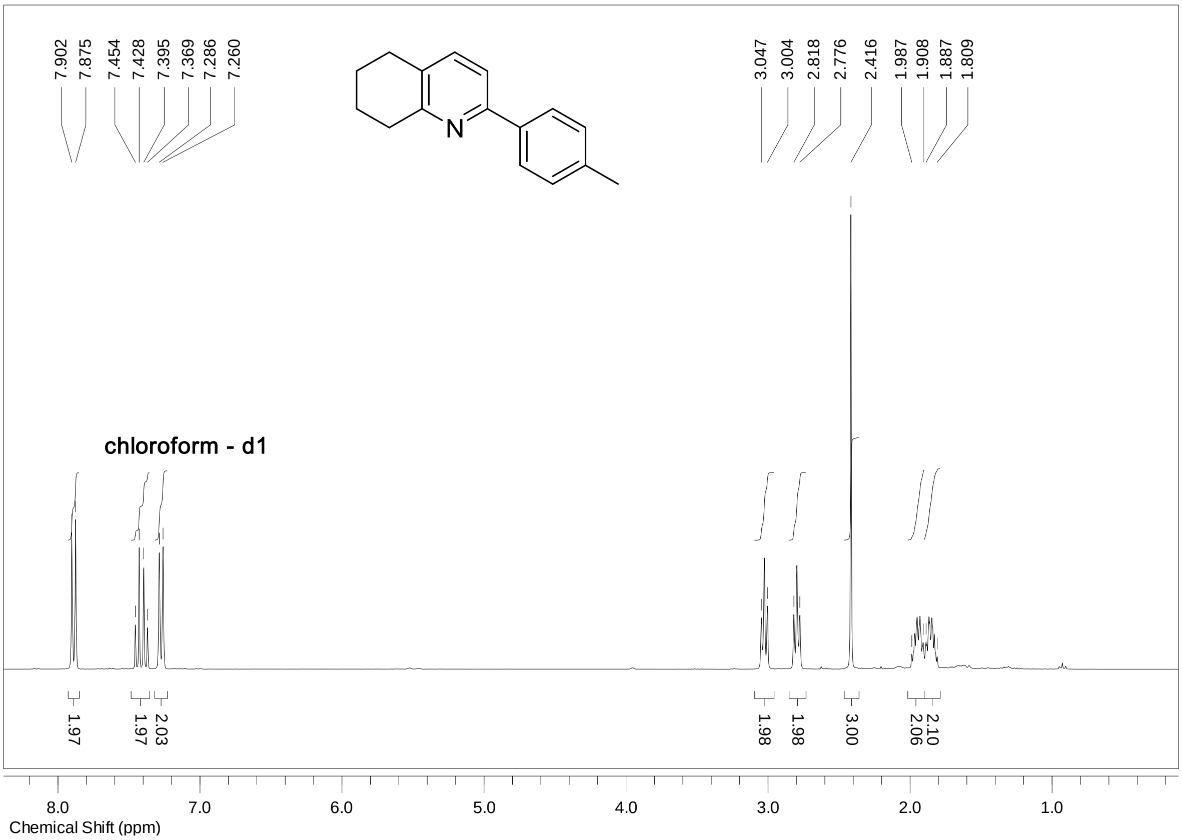

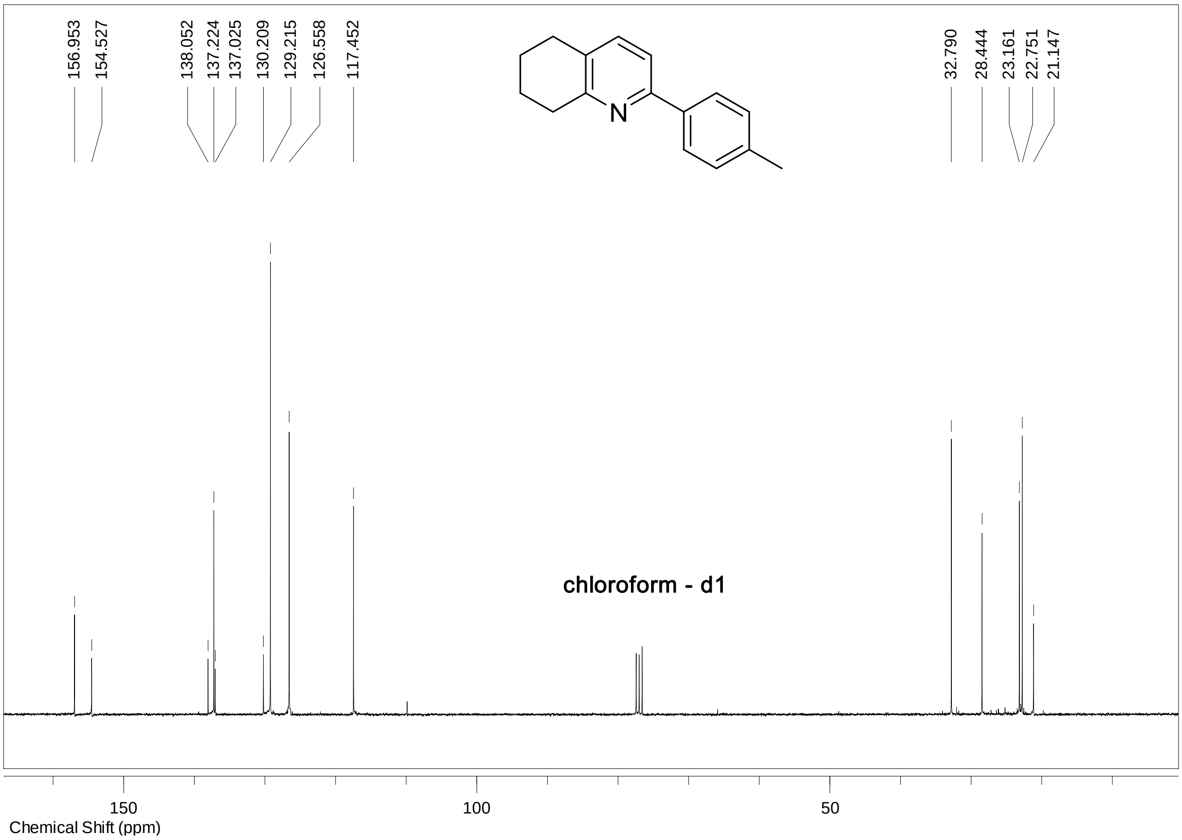

Supplementary Figure 6: NMR Spectra of compound 2c.

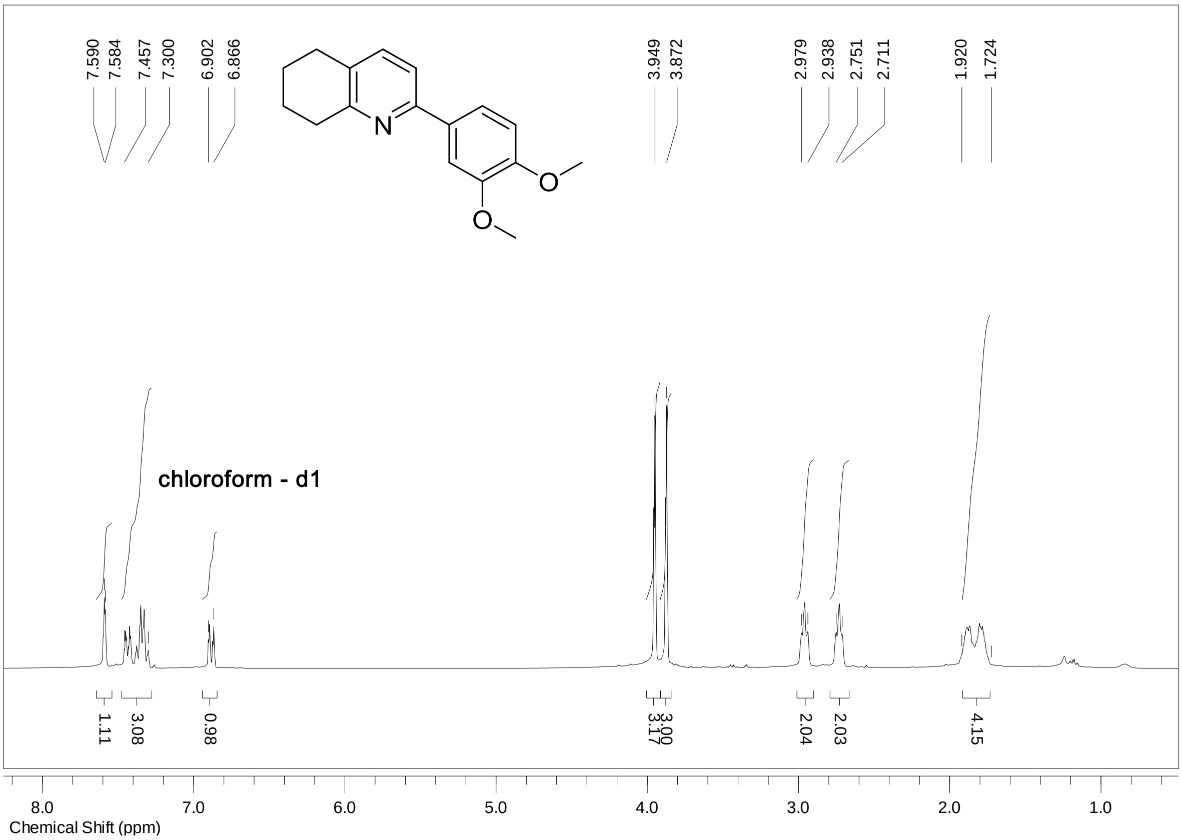

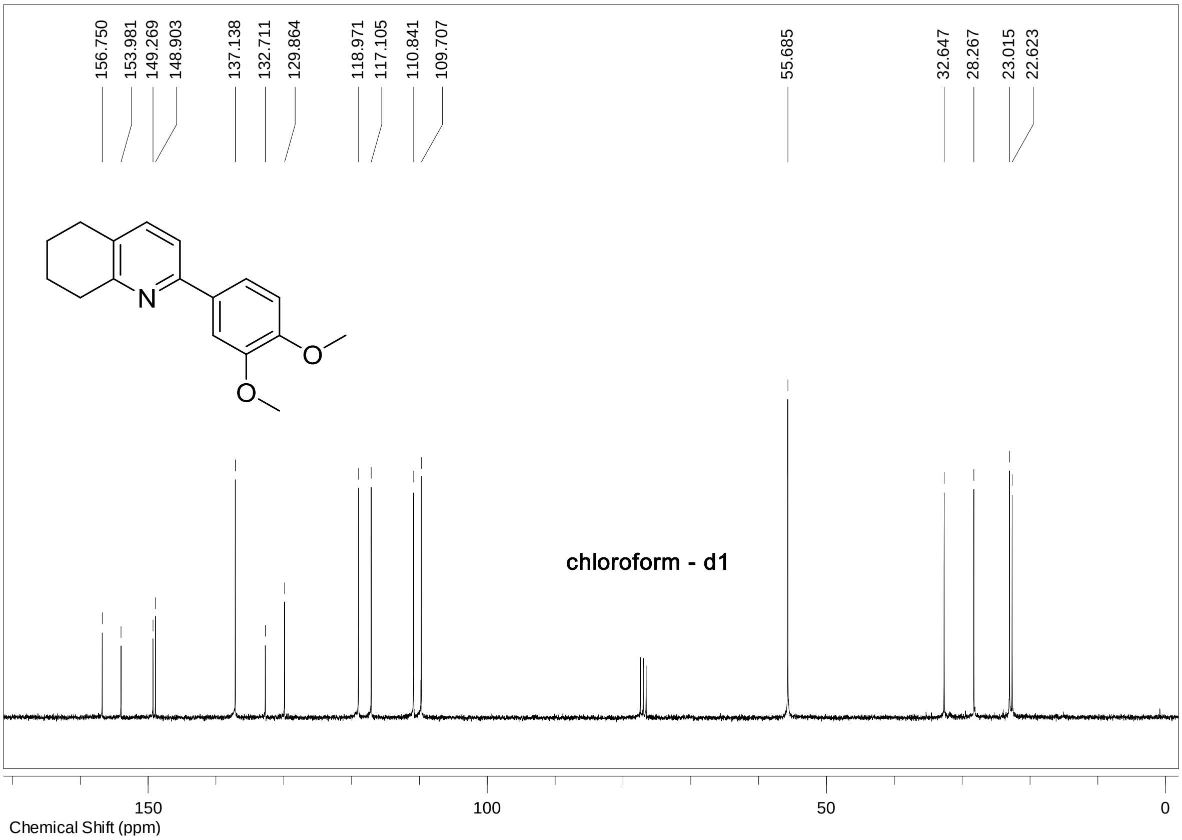

Supplementary Figure 7: NMR Spectra of compound 2d.

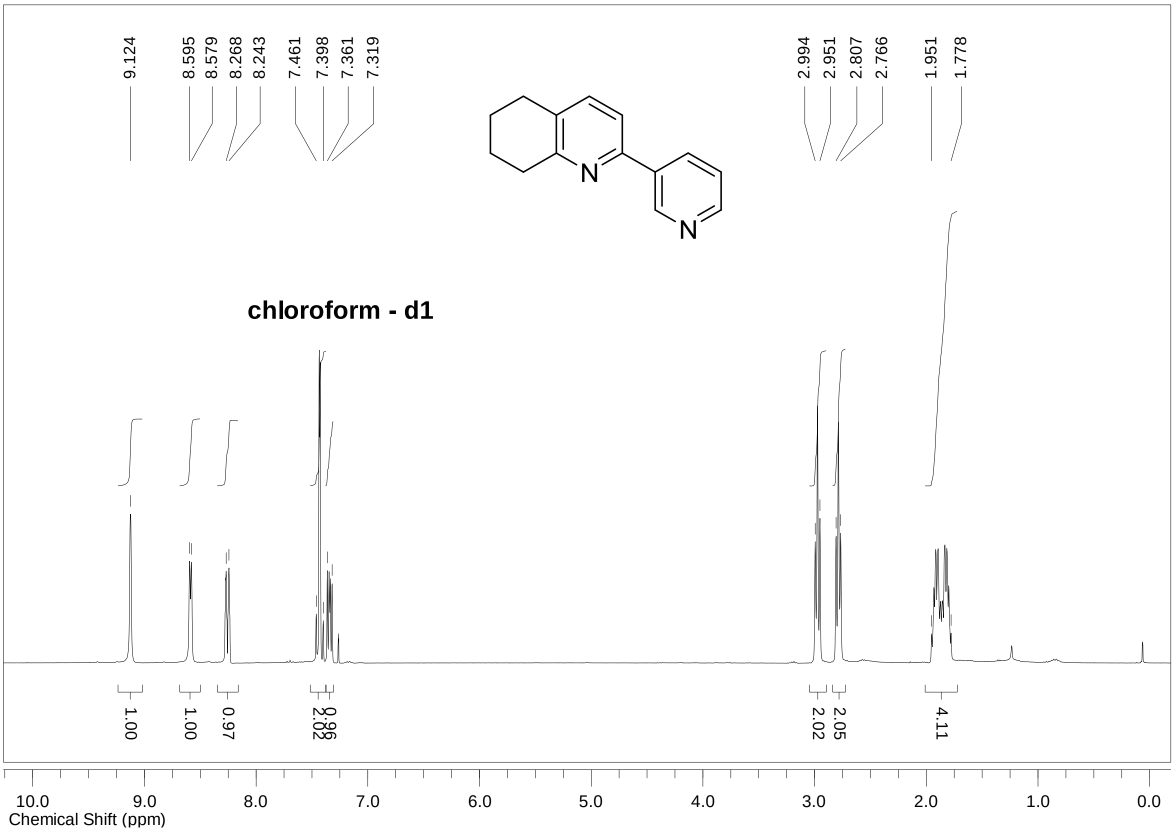

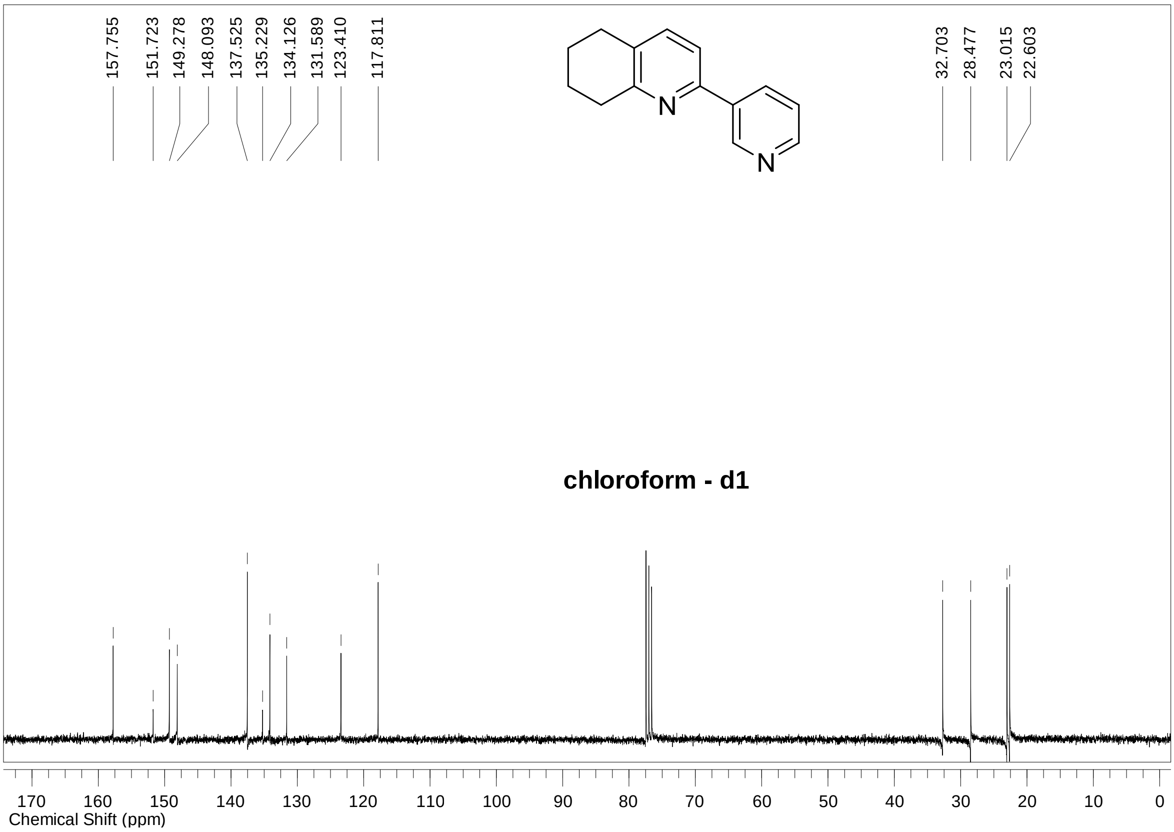

Supplementary Figure 8: NMR Spectra of compound 2e.

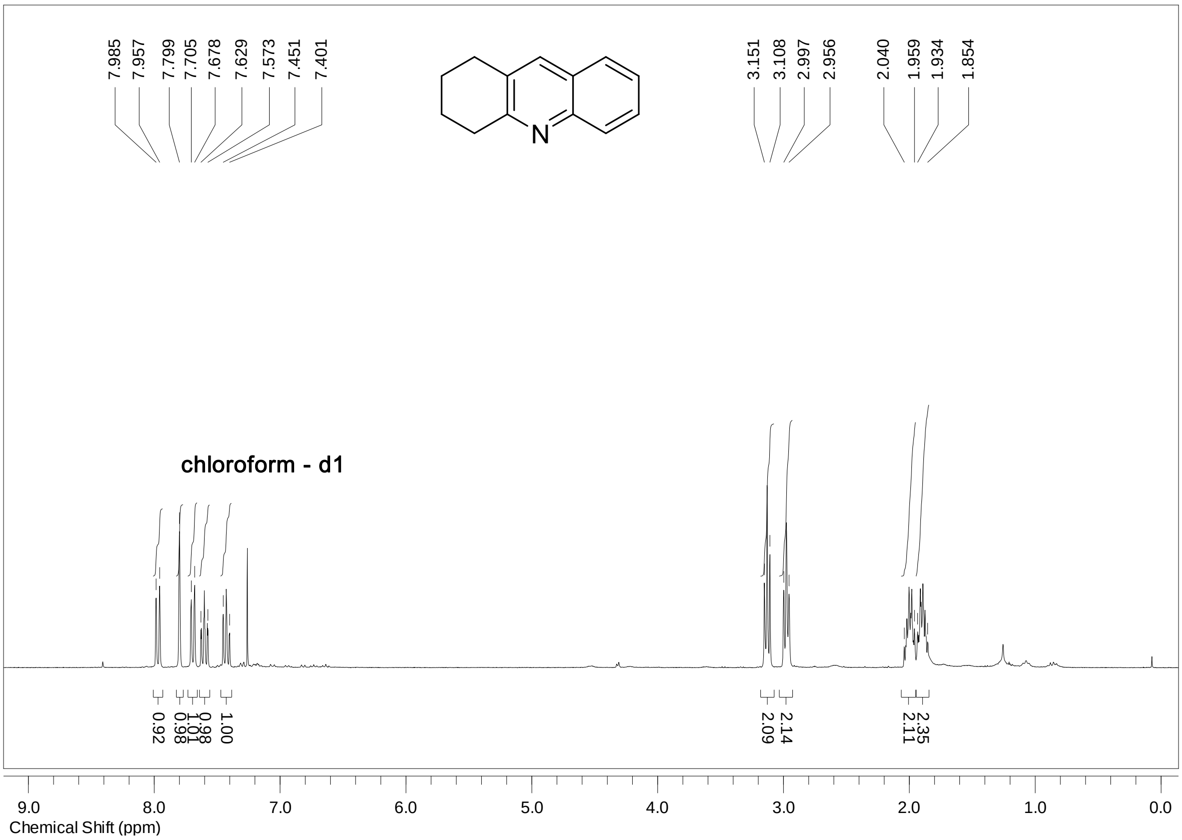

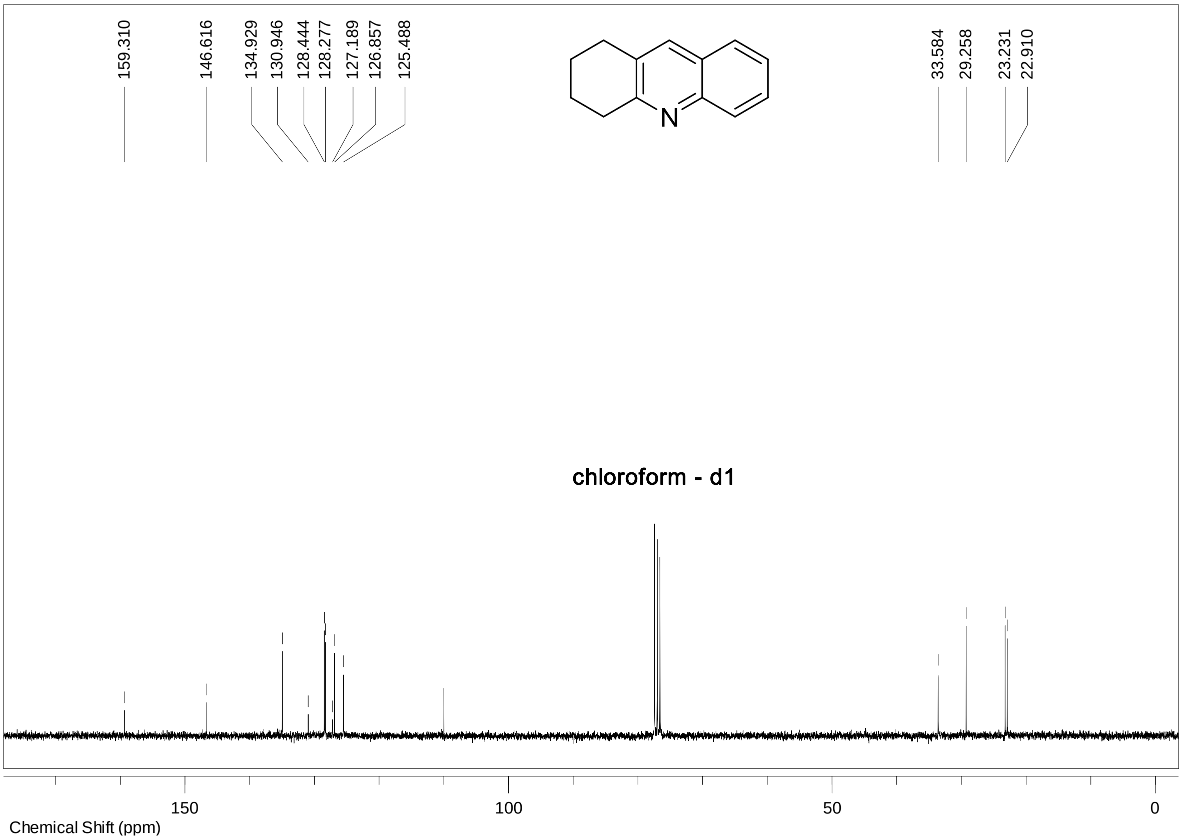

Supplementary Figure 9: NMR Spectra of compound 3a.

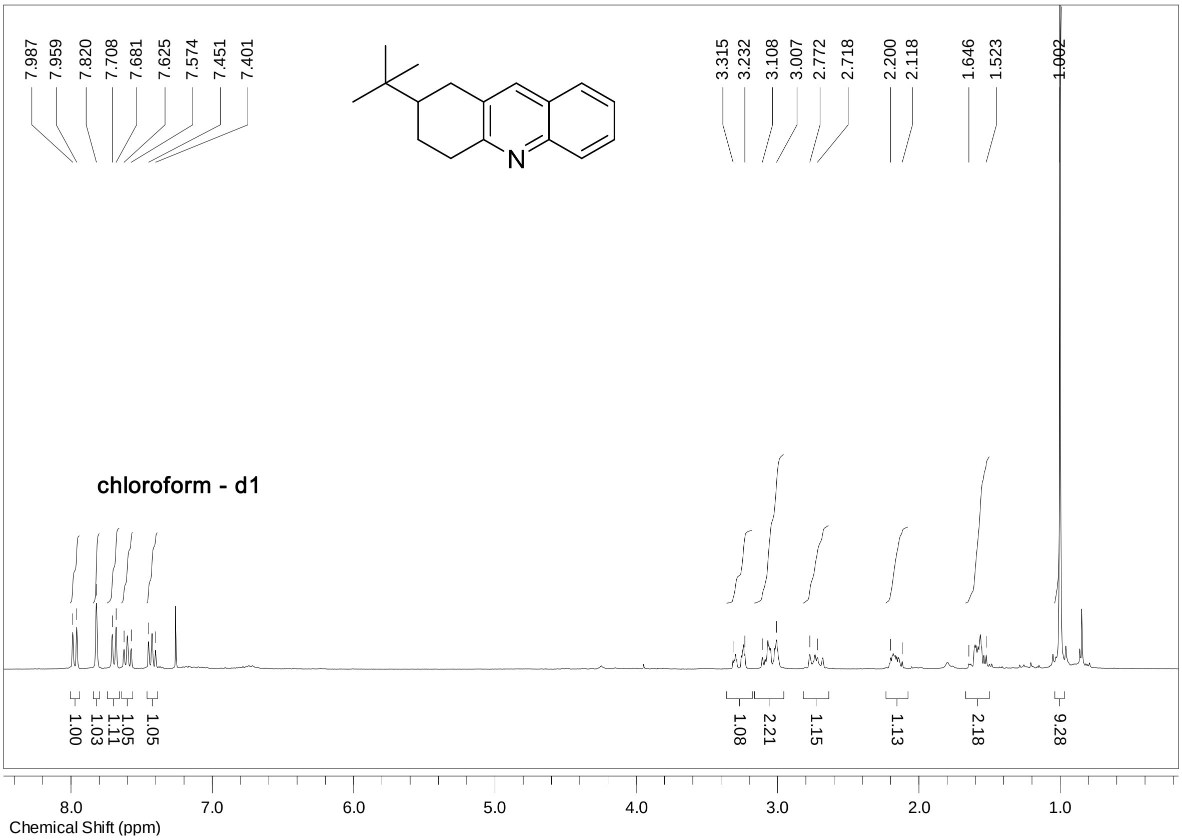

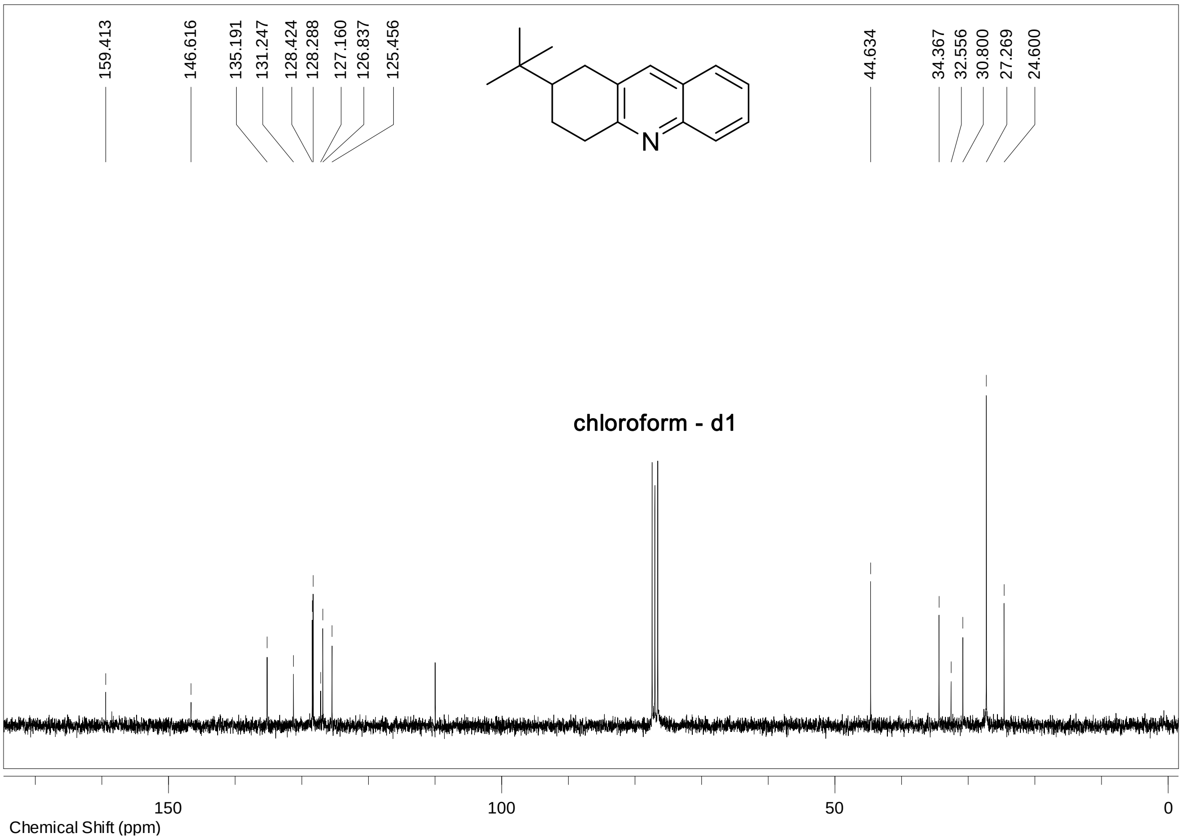

Supplementary Figure 10: NMR Spectra of compound 3b.

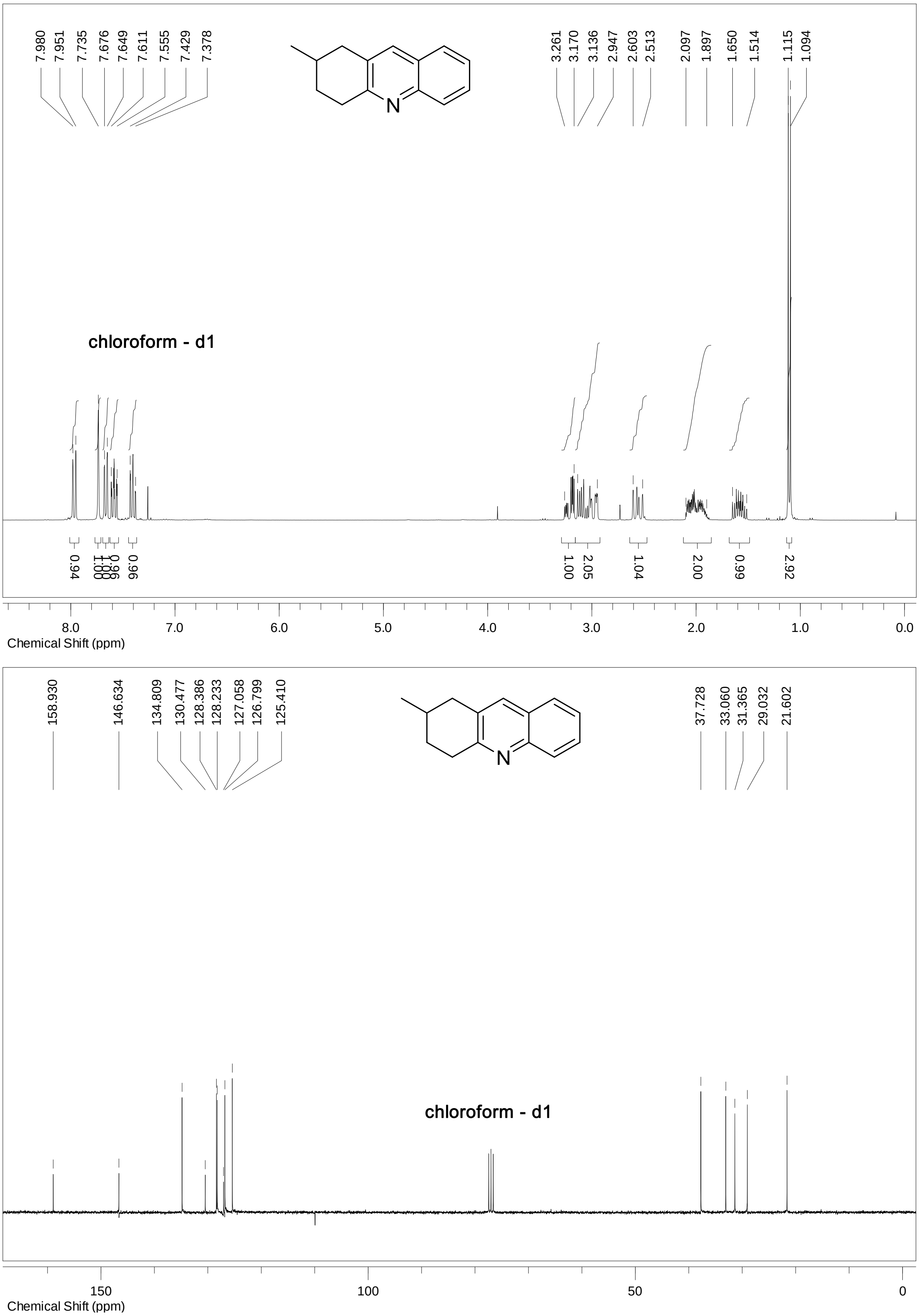

Supplementary Figure 11: NMR Spectra of compound 3c.

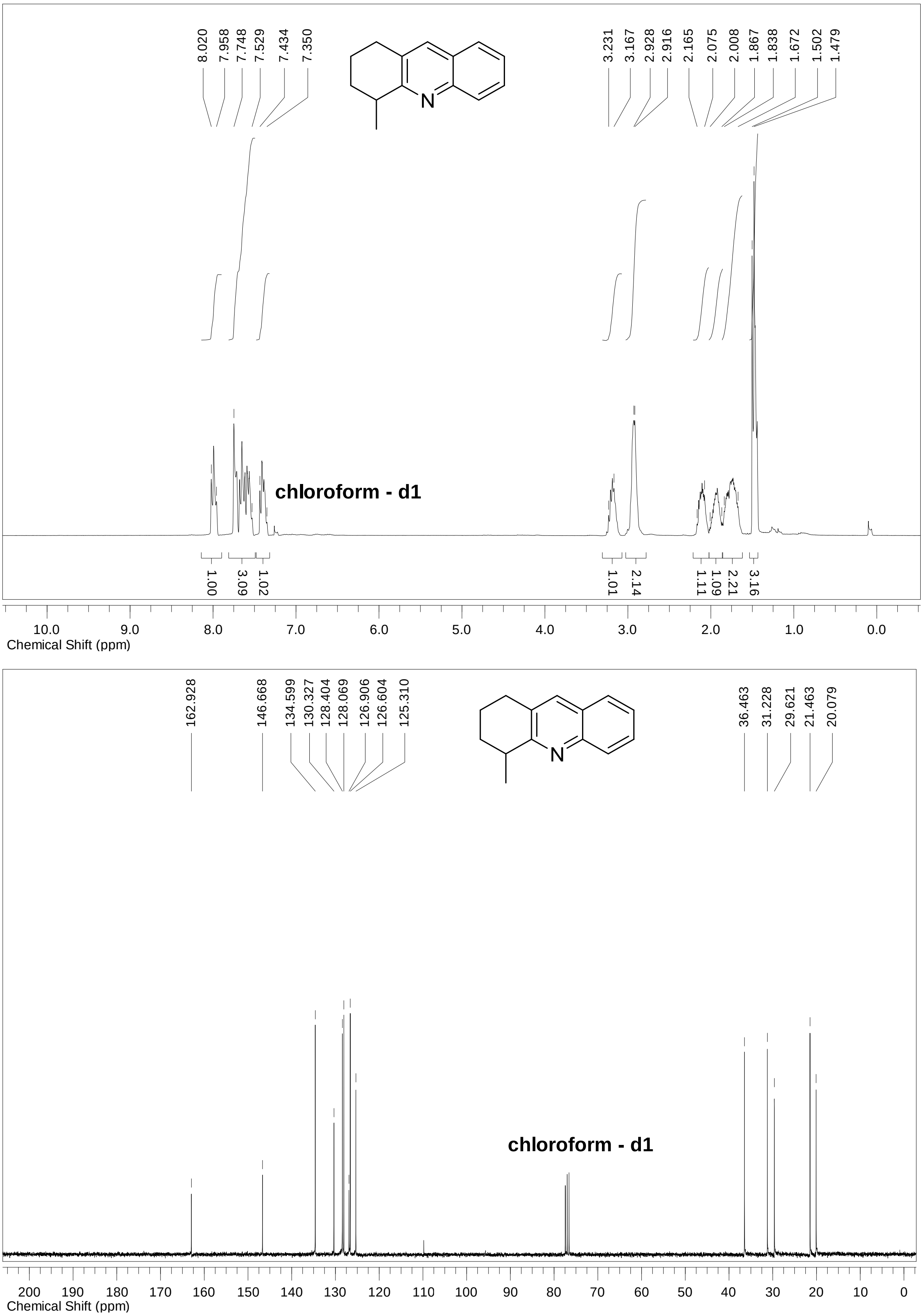

Supplementary Figure 12: NMR Spectra of compound 3d.

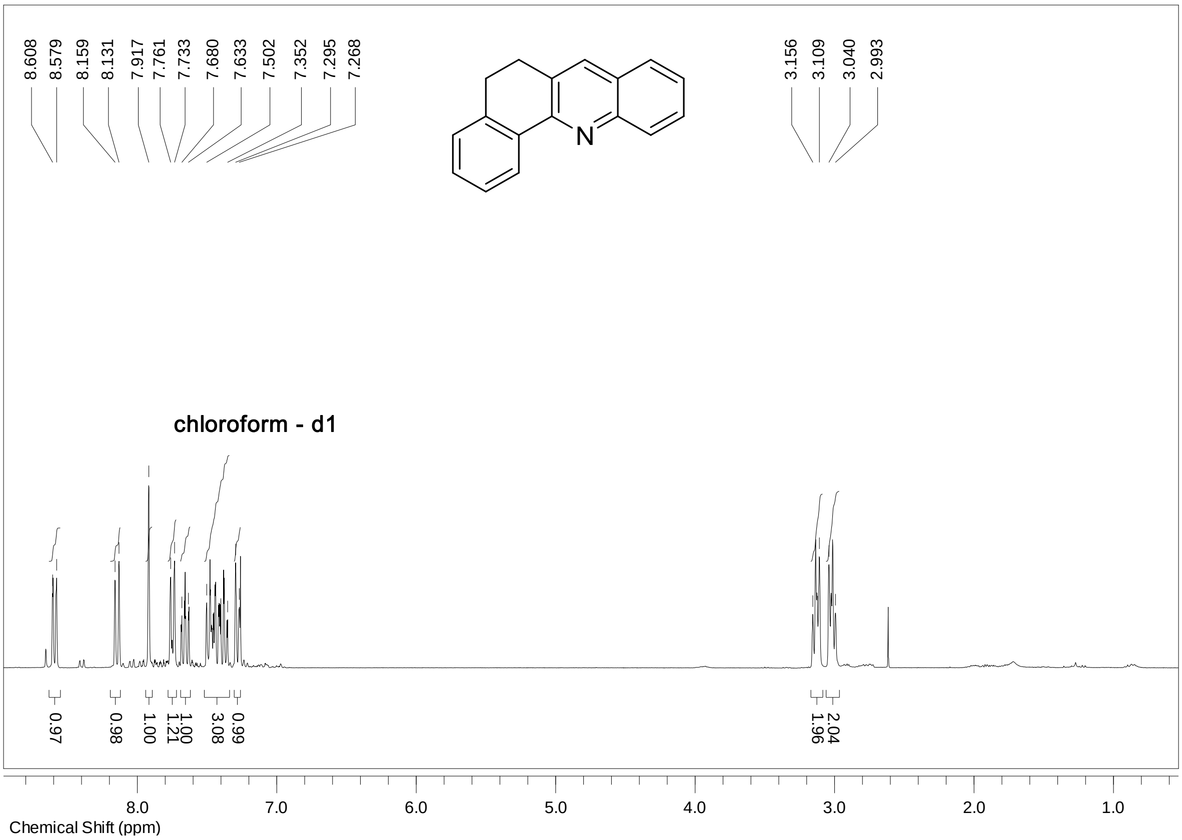

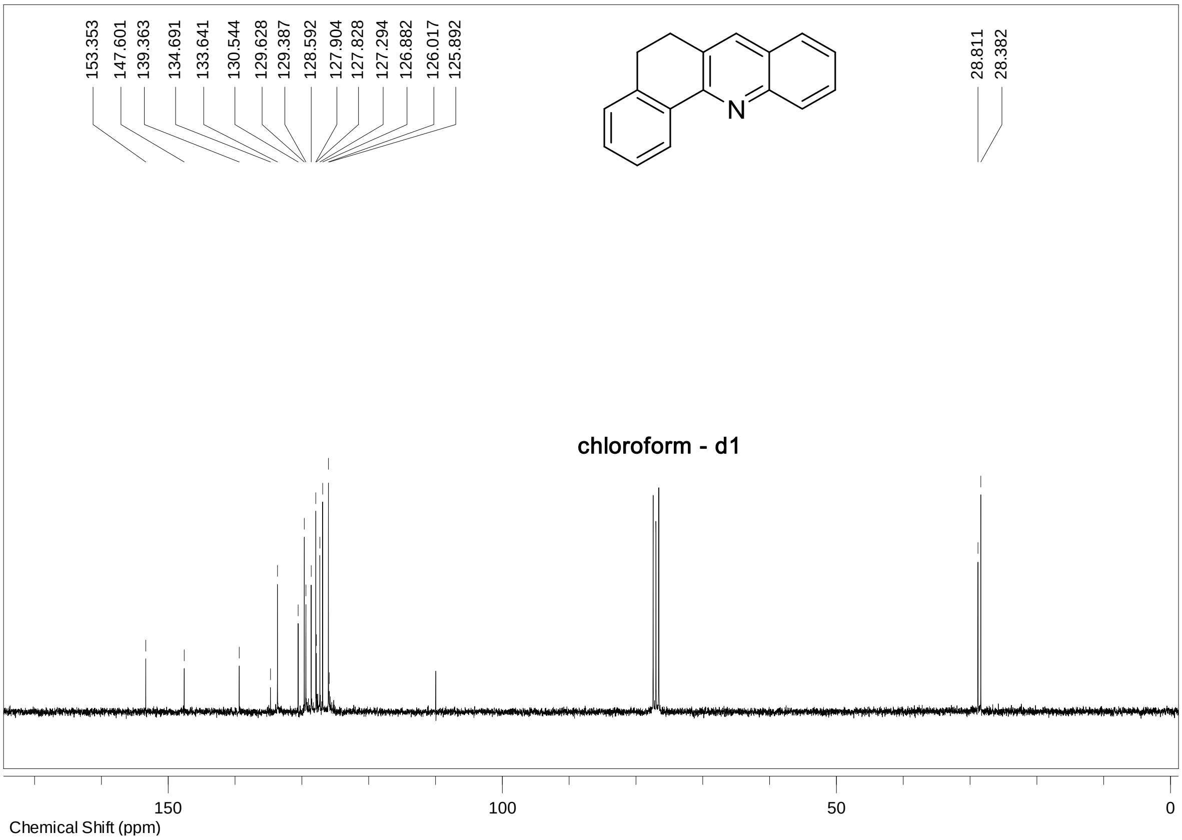

Supplementary Figure 13: NMR Spectra of compound 3f.

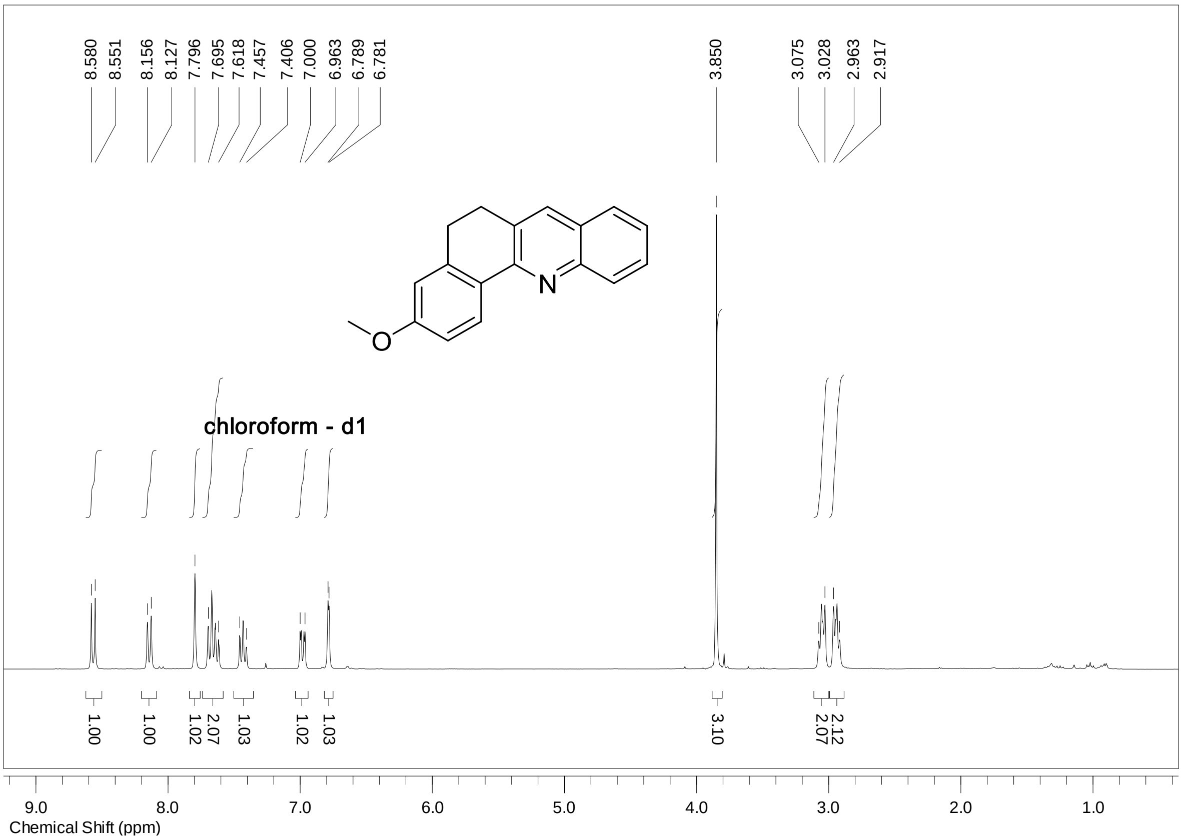

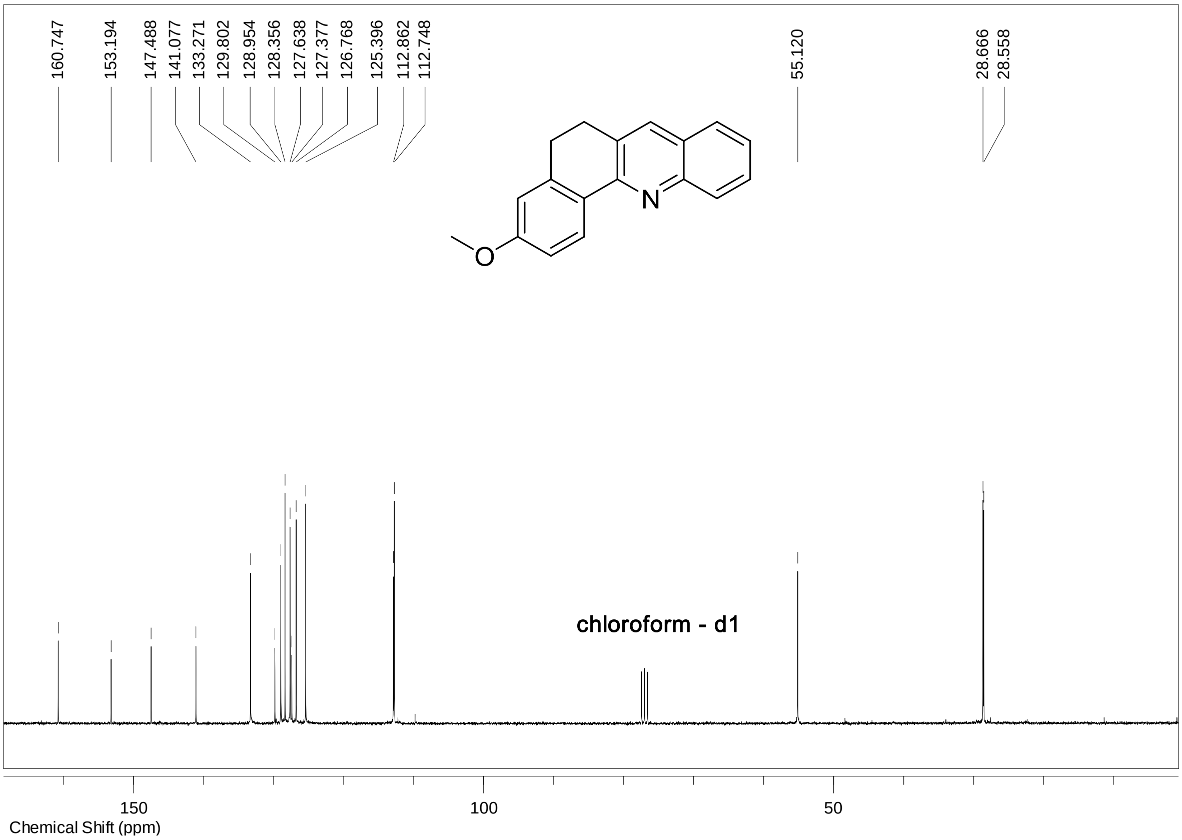

Supplementary Figure 14: NMR Spectra of compound 3g.

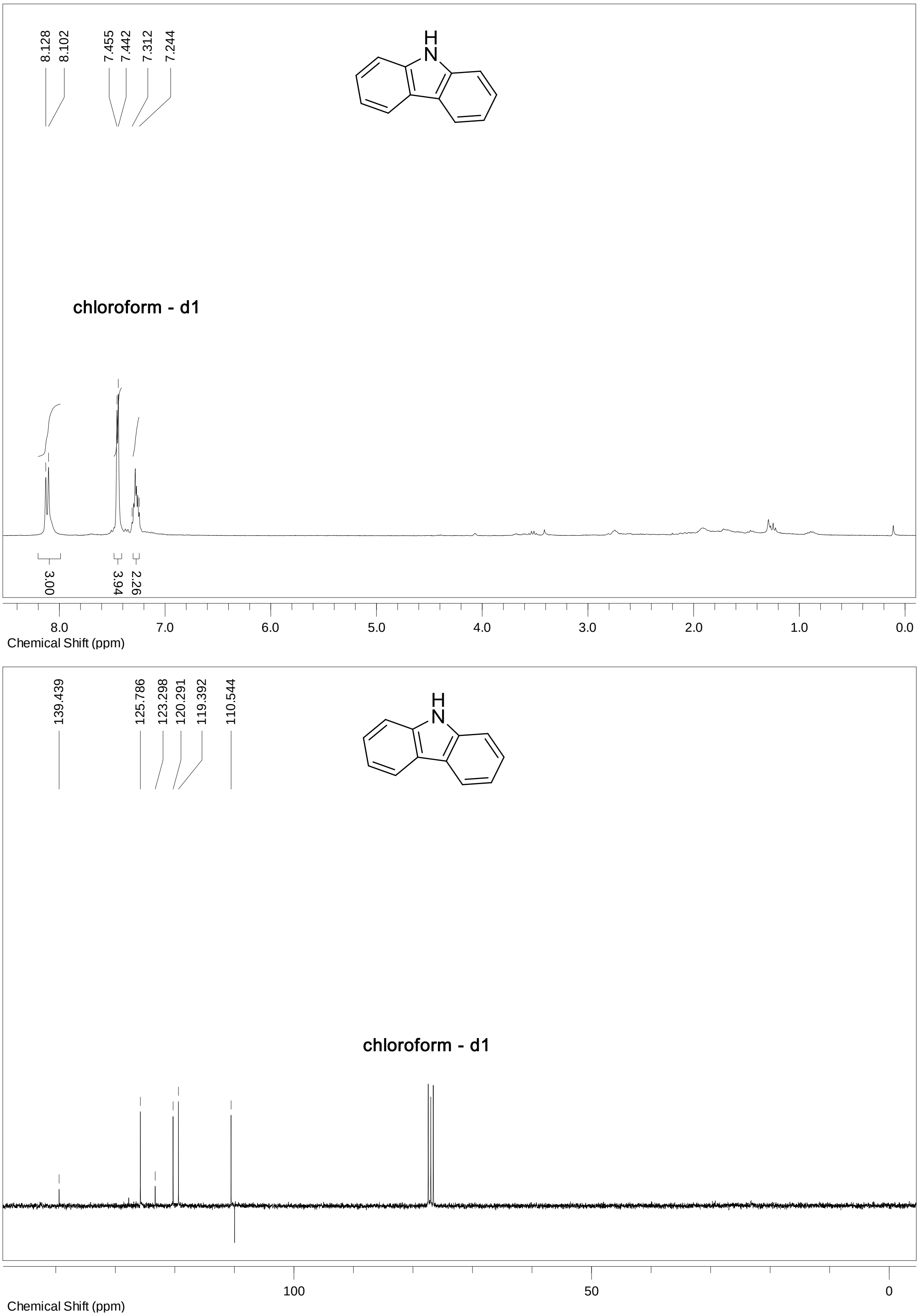

Supplementary Figure 15: NMR Spectra of compound 4a.

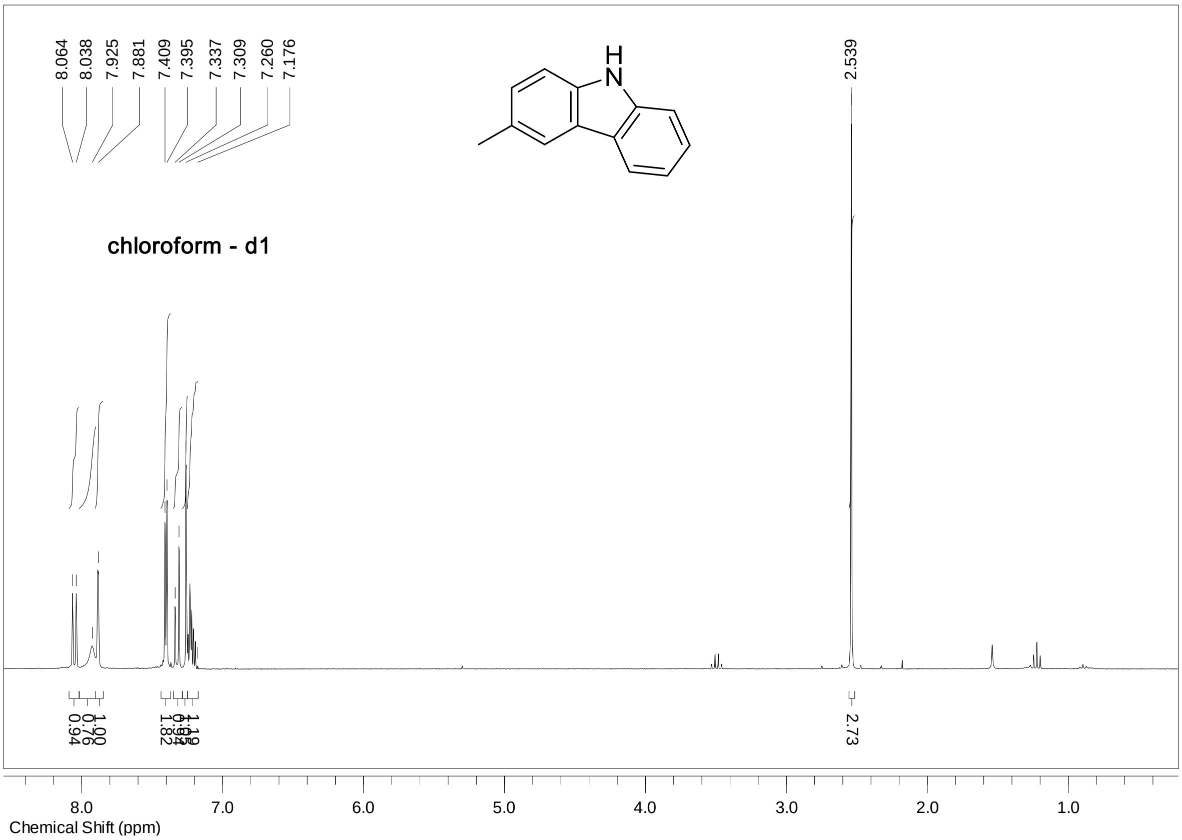

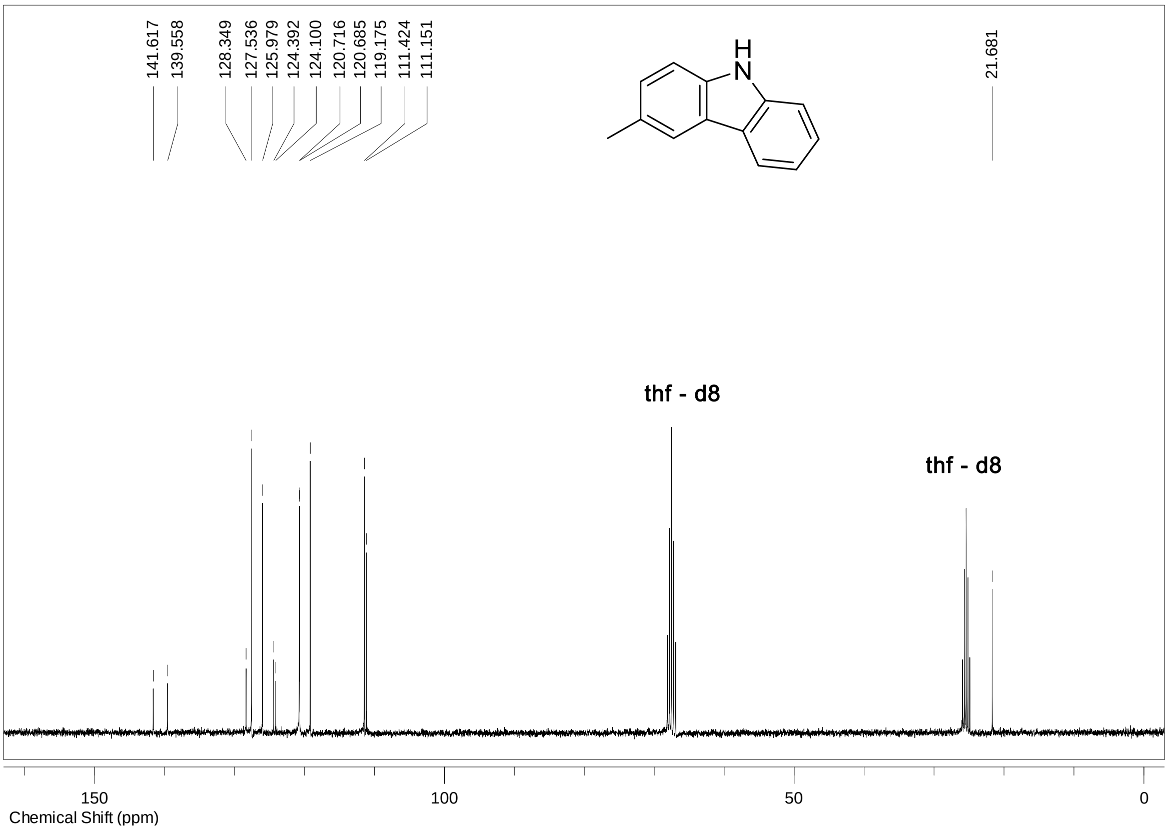

Supplementary Figure 16: NMR Spectra of compound 4b.

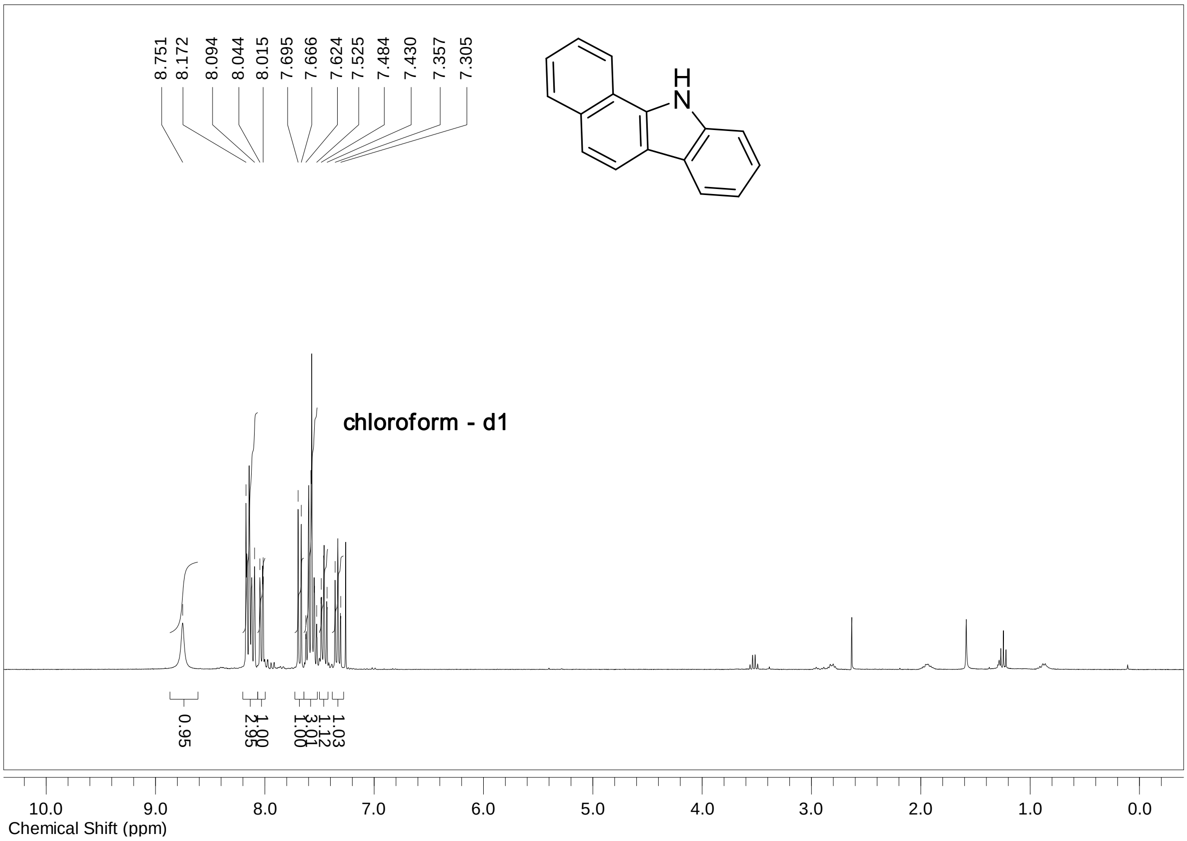

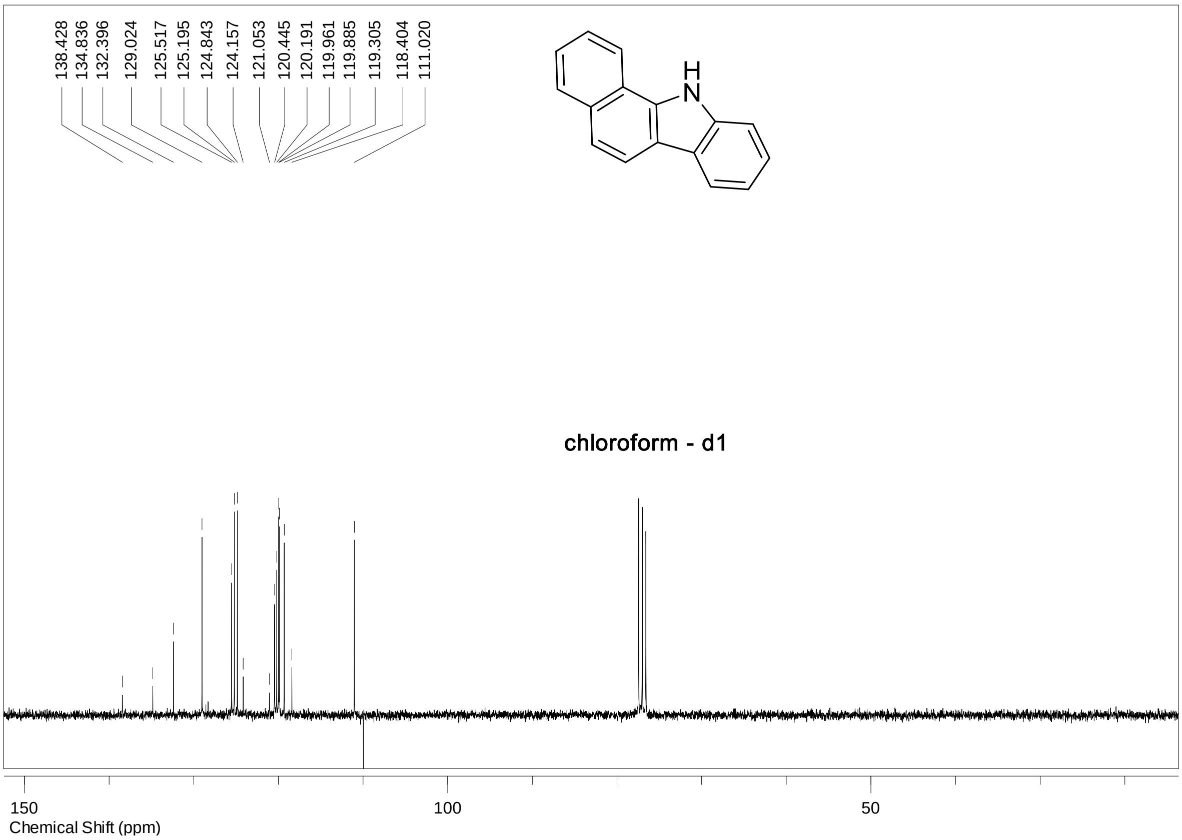

Supplementary Figure 17: NMR Spectra of compound 4c.

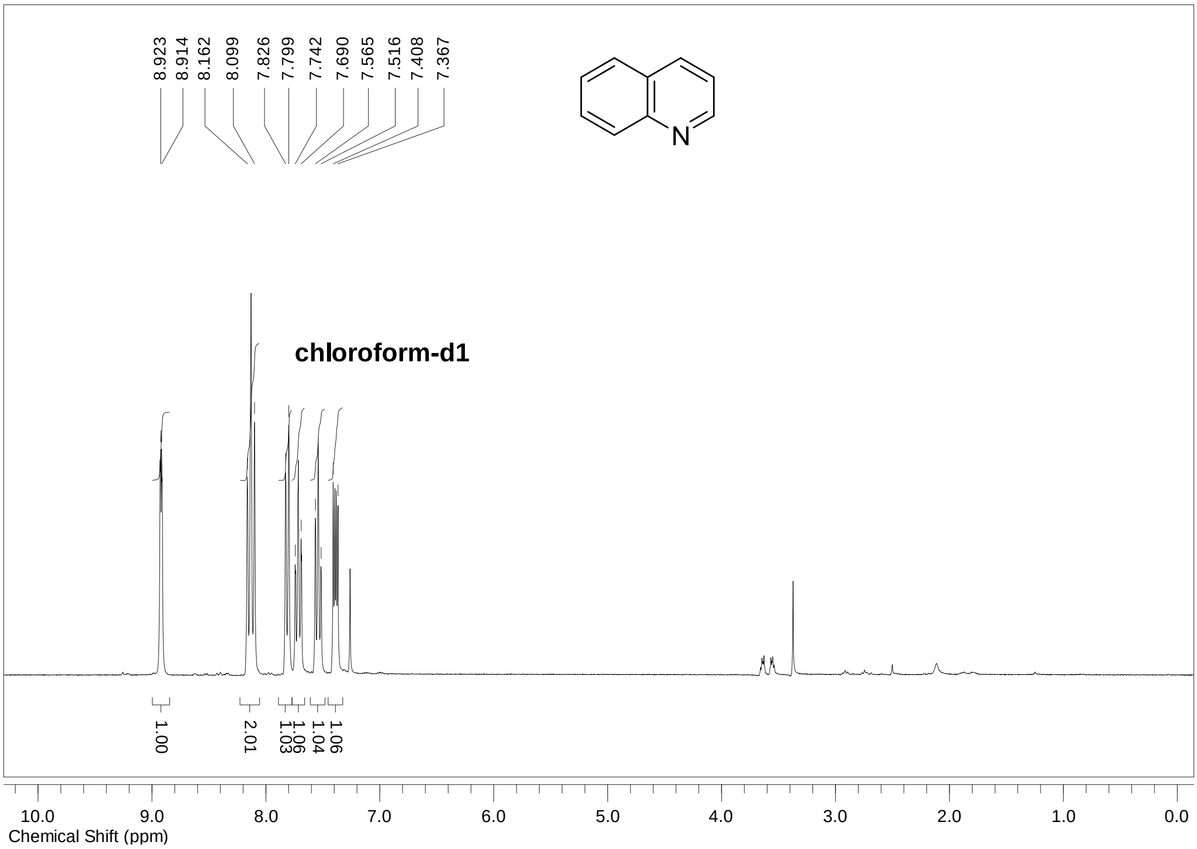

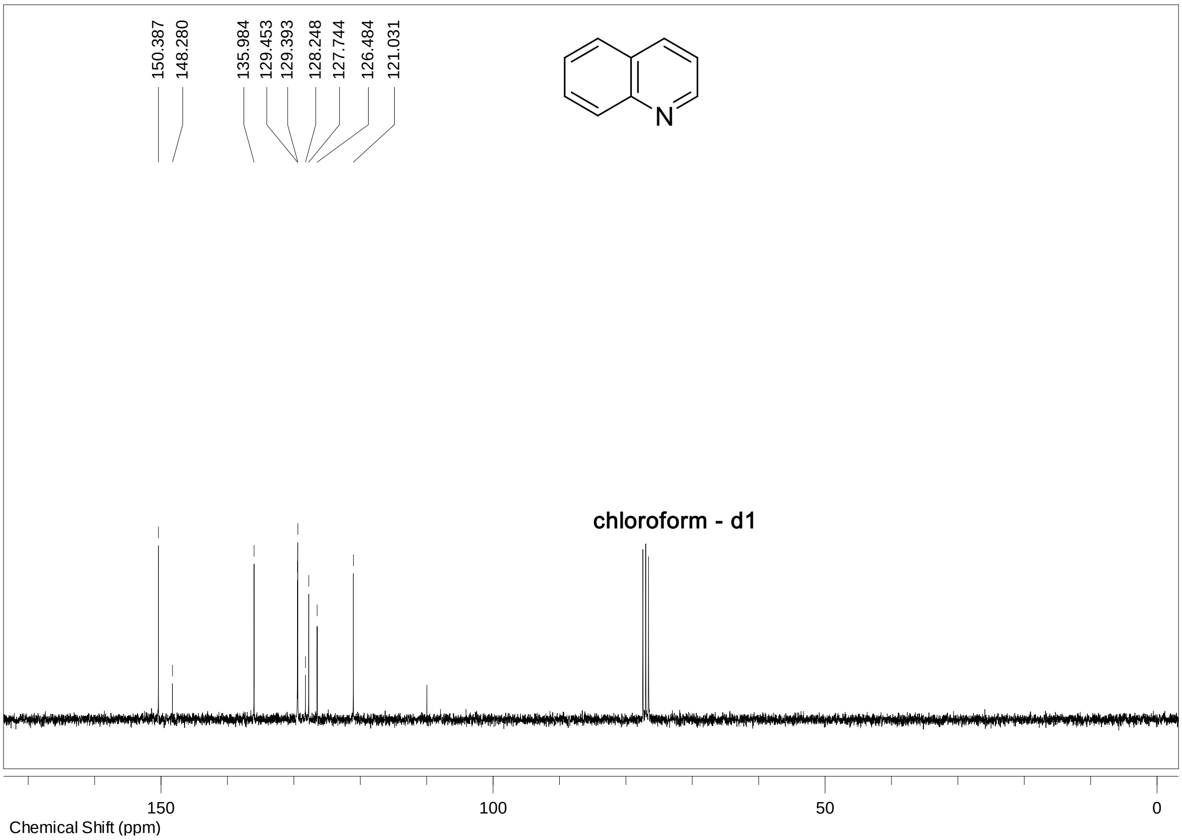

Supplementary Figure 18: NMR Spectra of compound 5a.

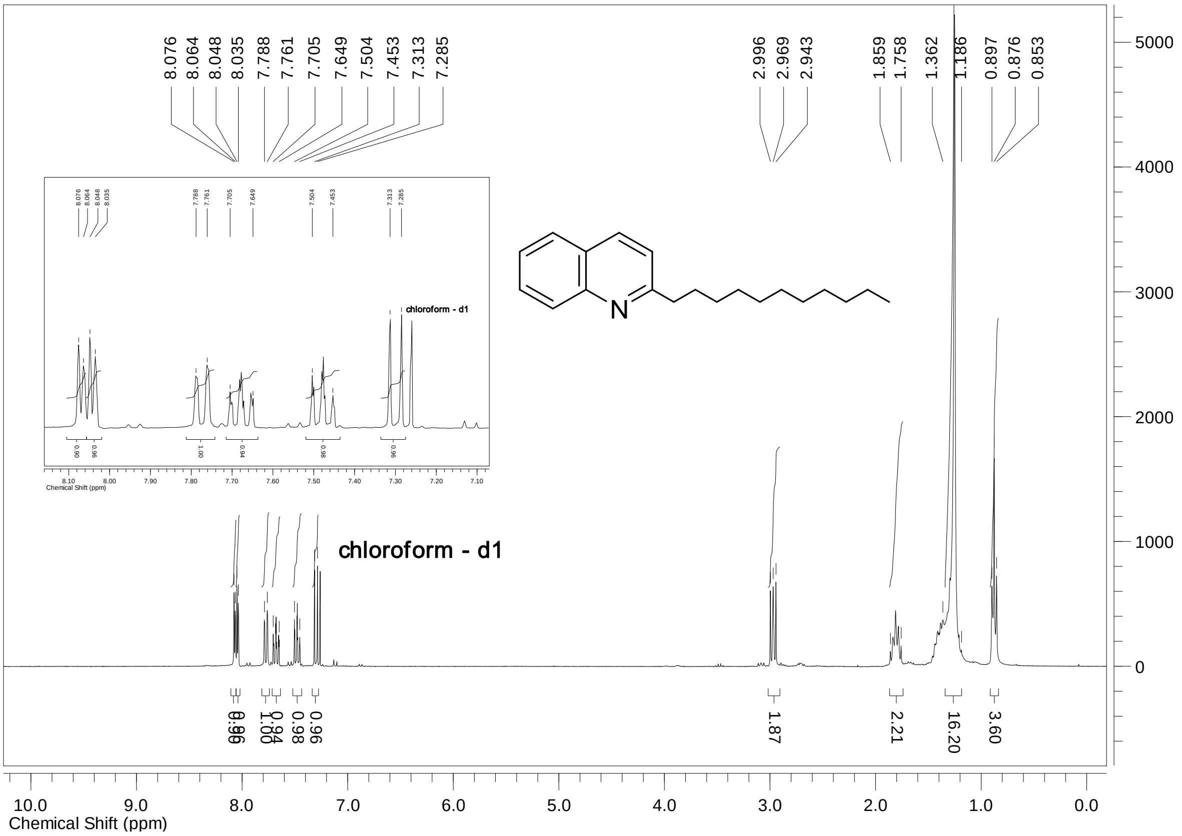

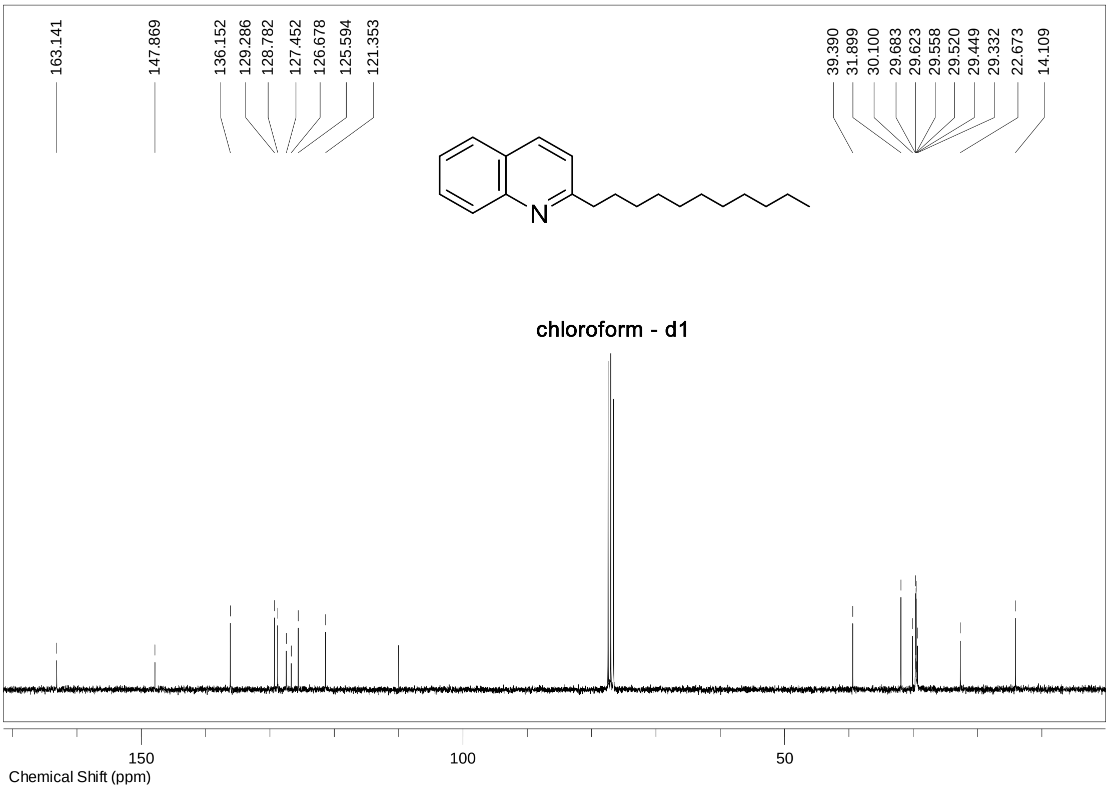

Supplementary Figure 19: NMR Spectra of compound 5b.

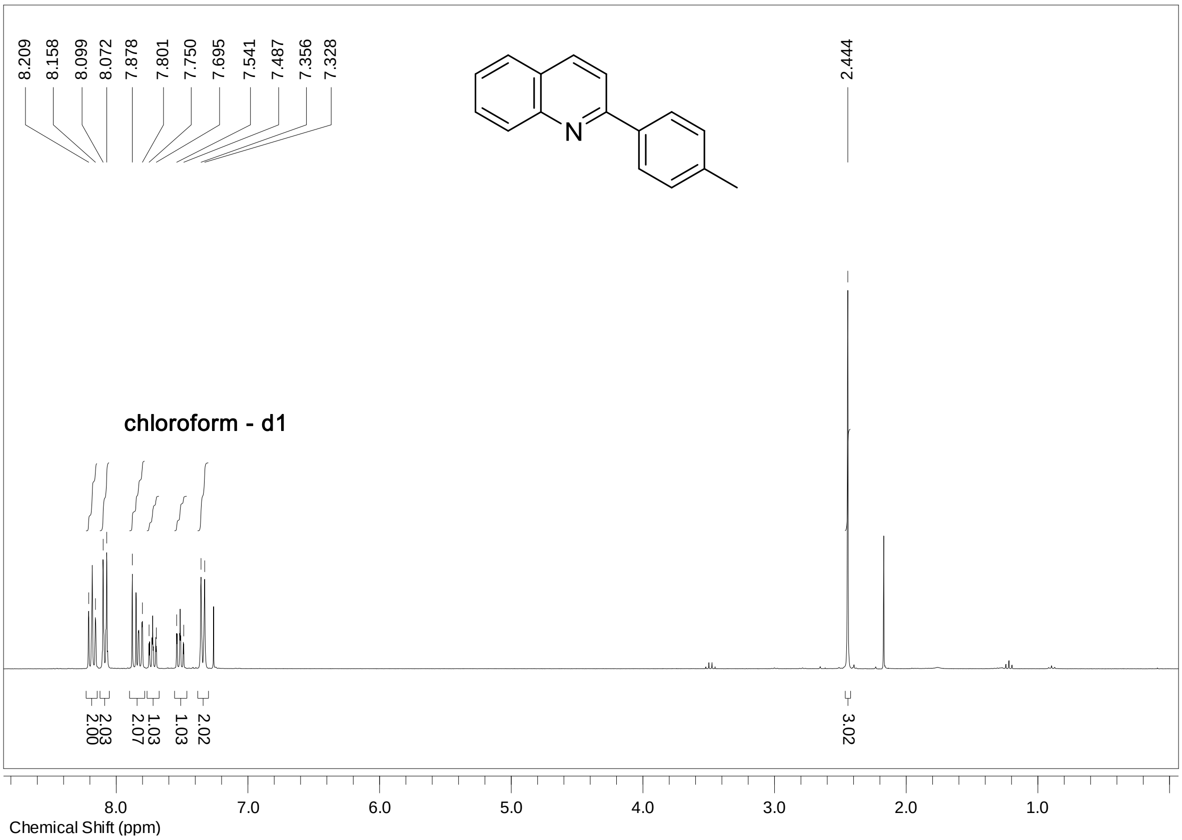

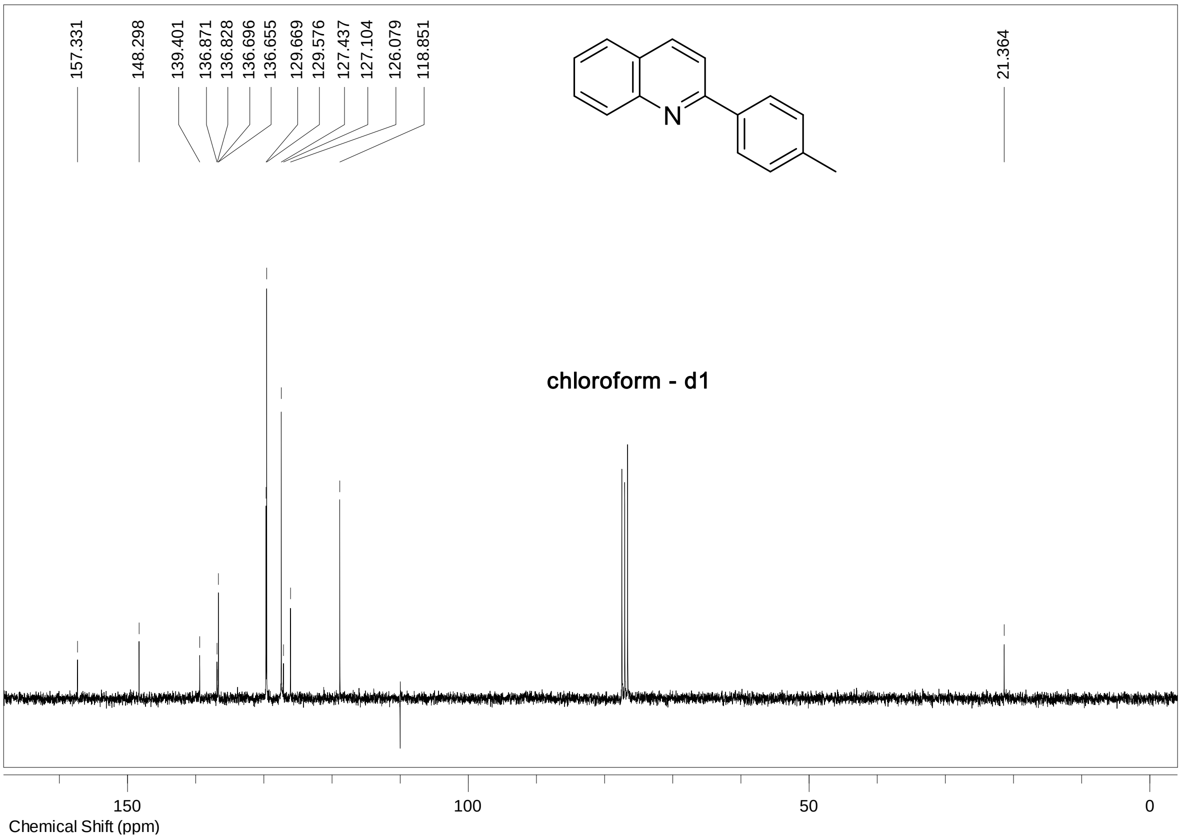

Supplementary Figure 20: NMR Spectra of compound 5c.

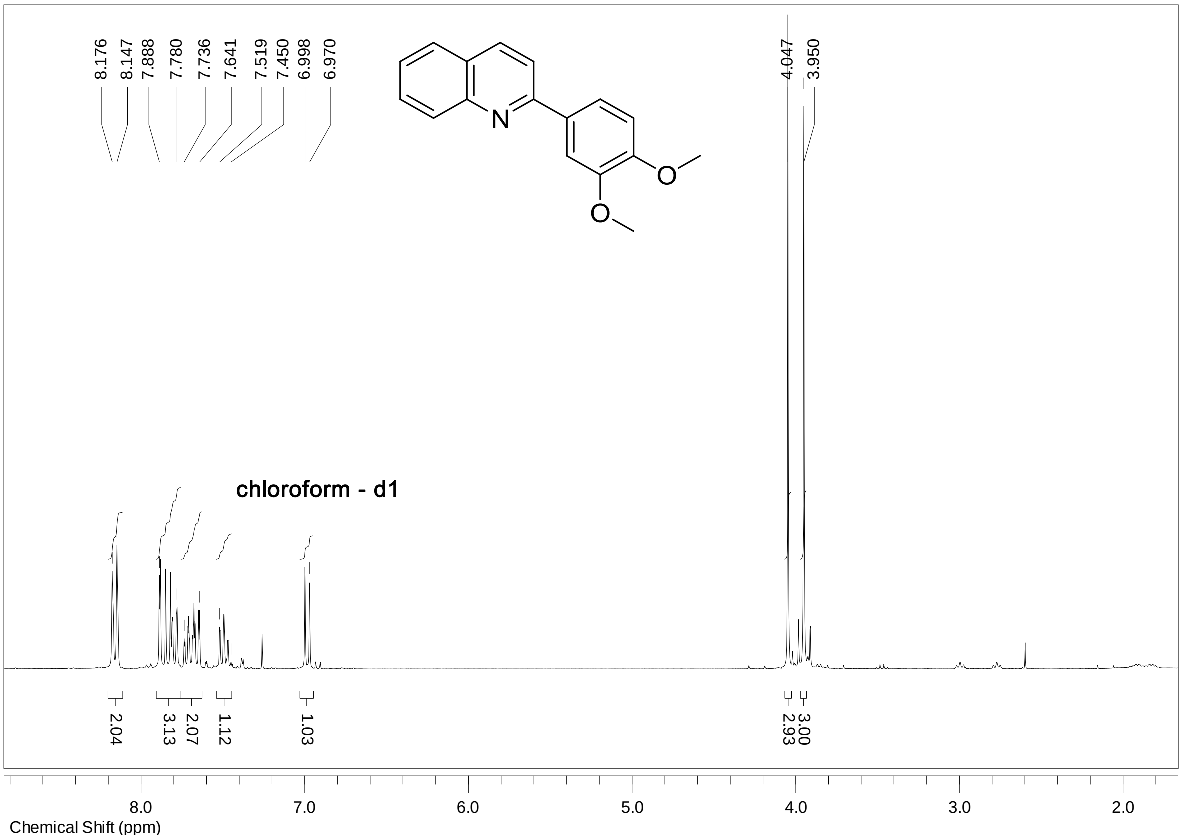

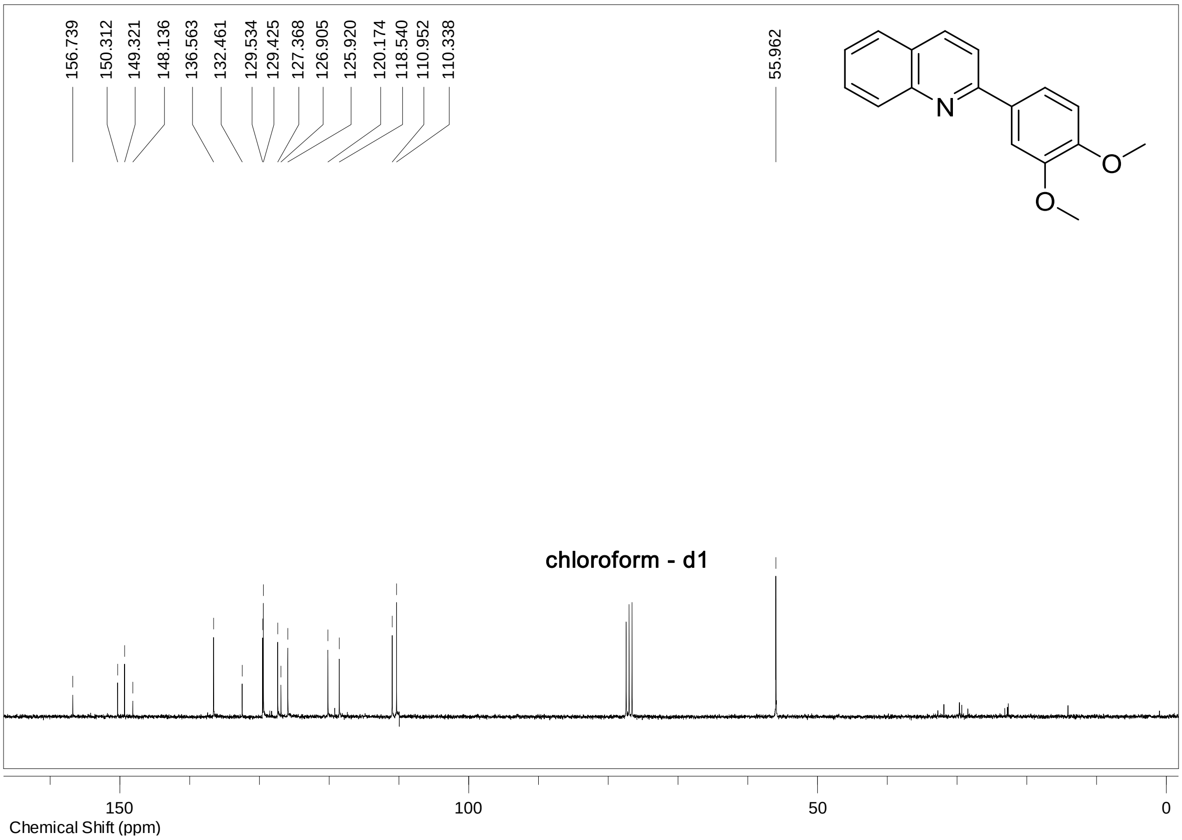

Supplementary Figure 21: NMR Spectra of compound 5d.

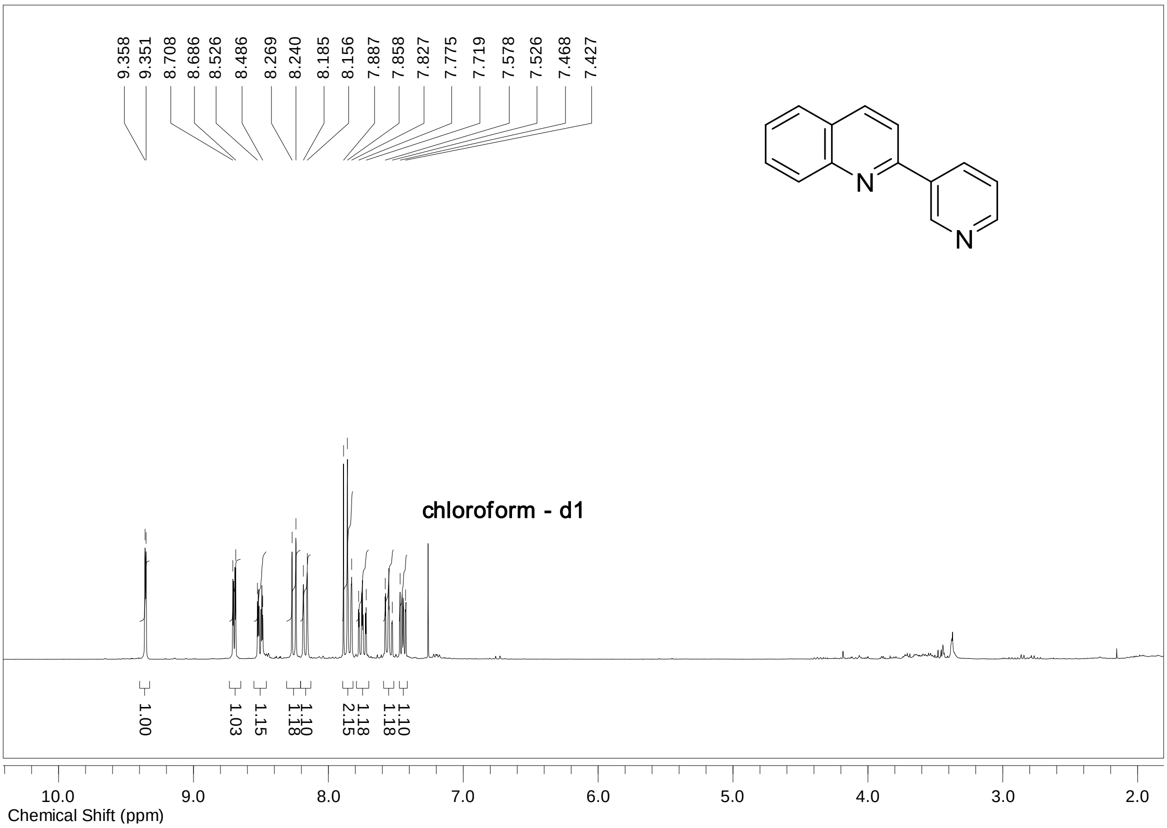

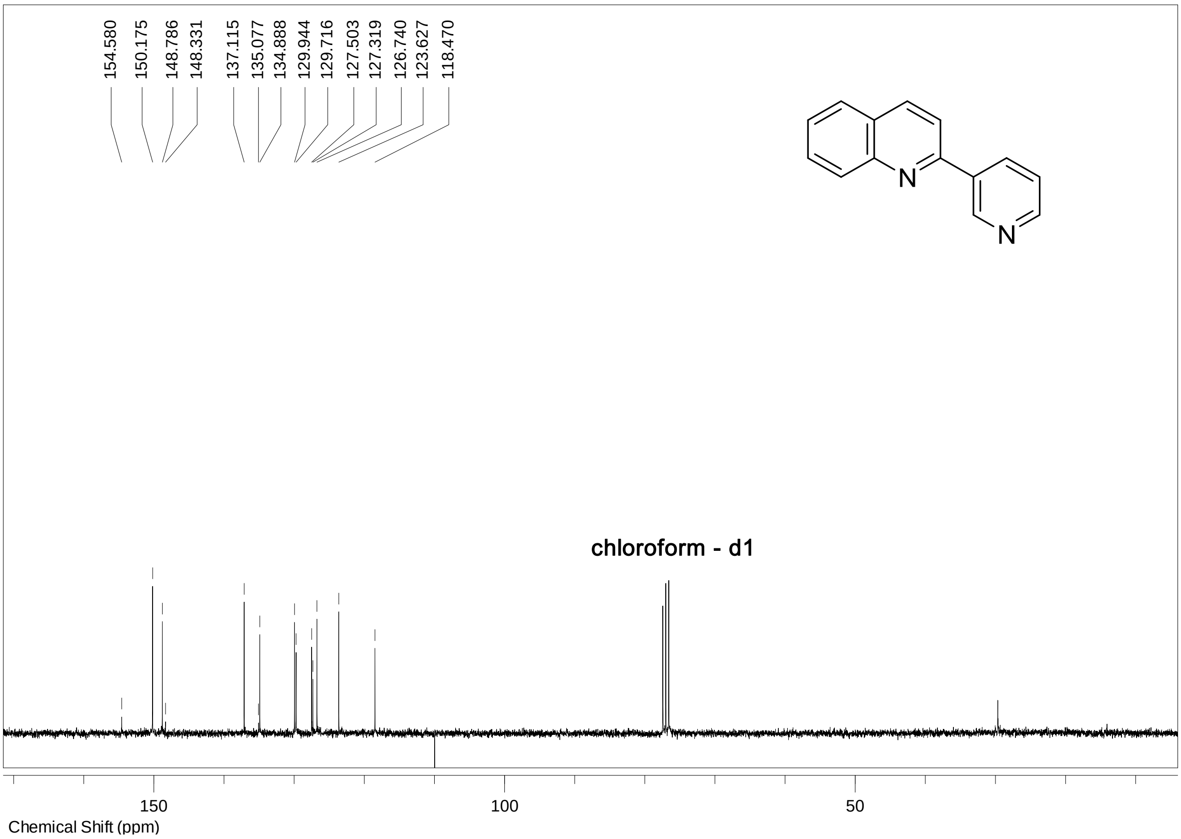

Supplementary Figure 22: NMR Spectra of compound 5e.

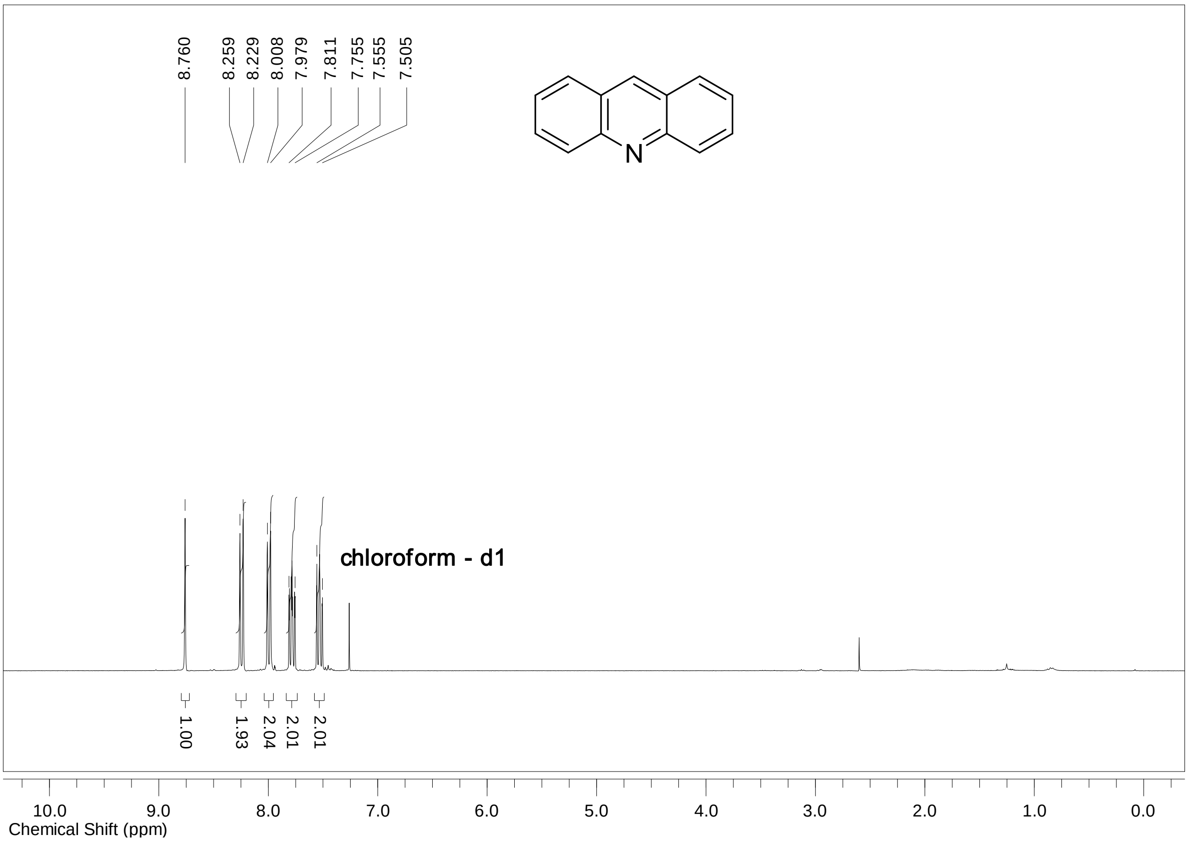

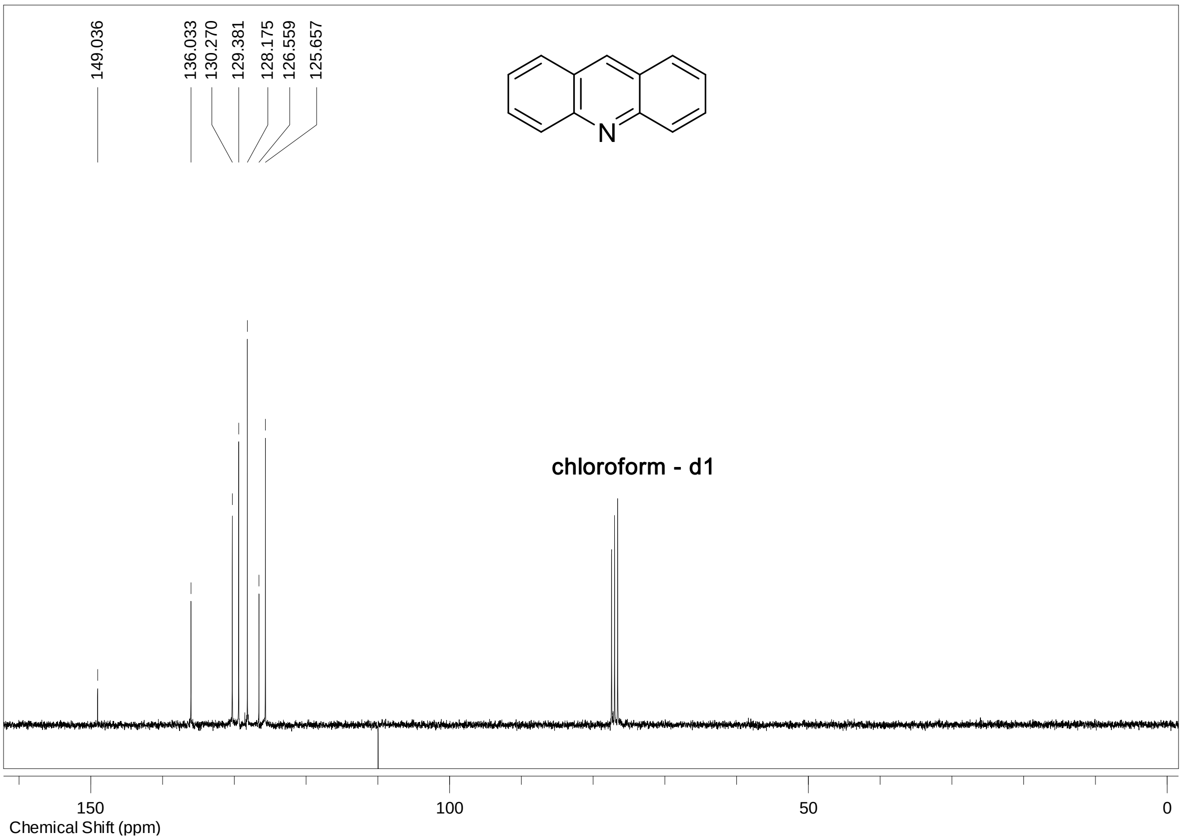

Supplementary Figure 23: NMR Spectra of compound 6a.

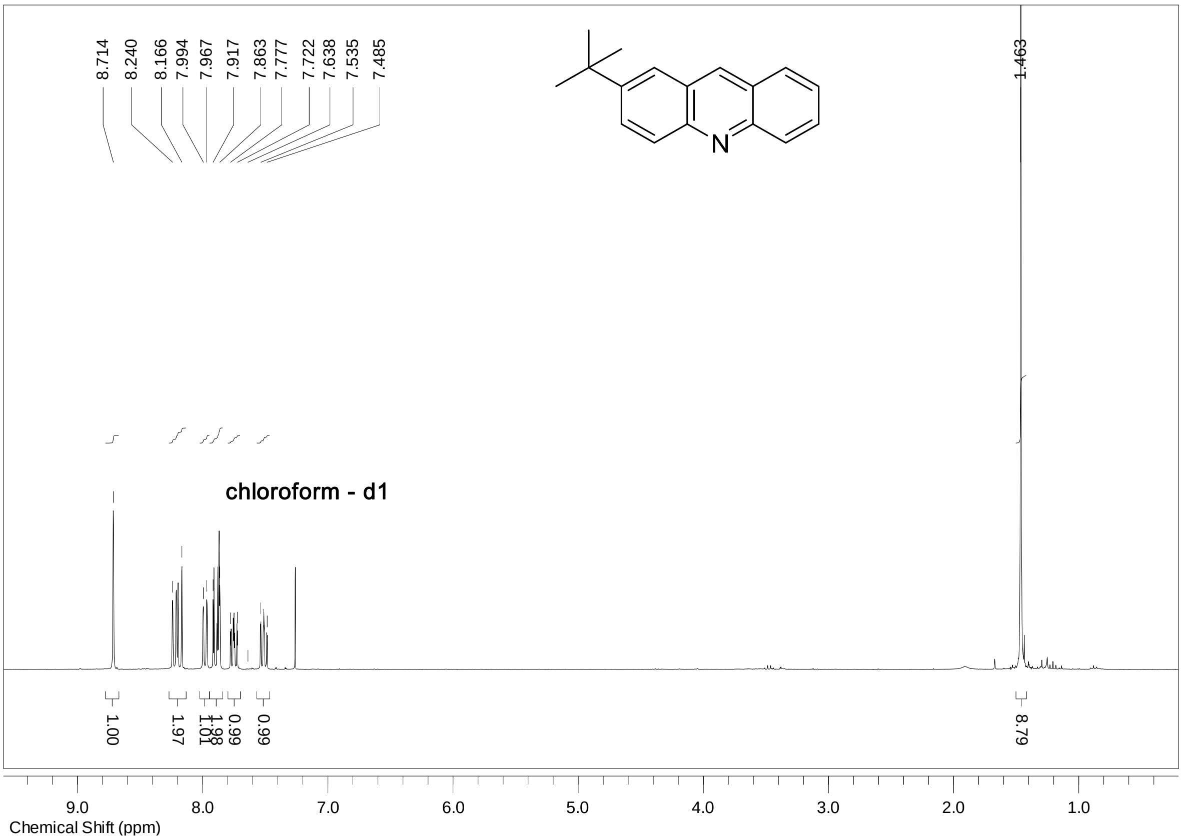

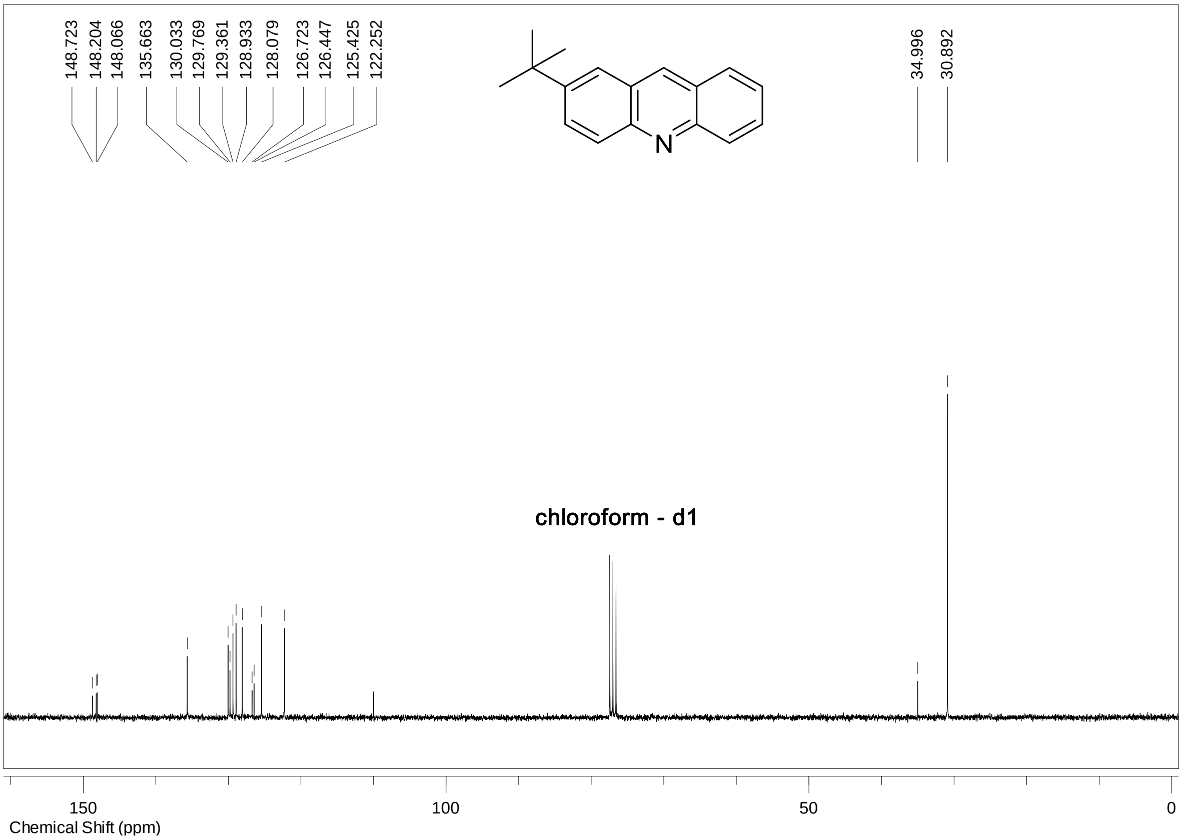

Supplementary Figure 24: NMR Spectra of compound 6b.

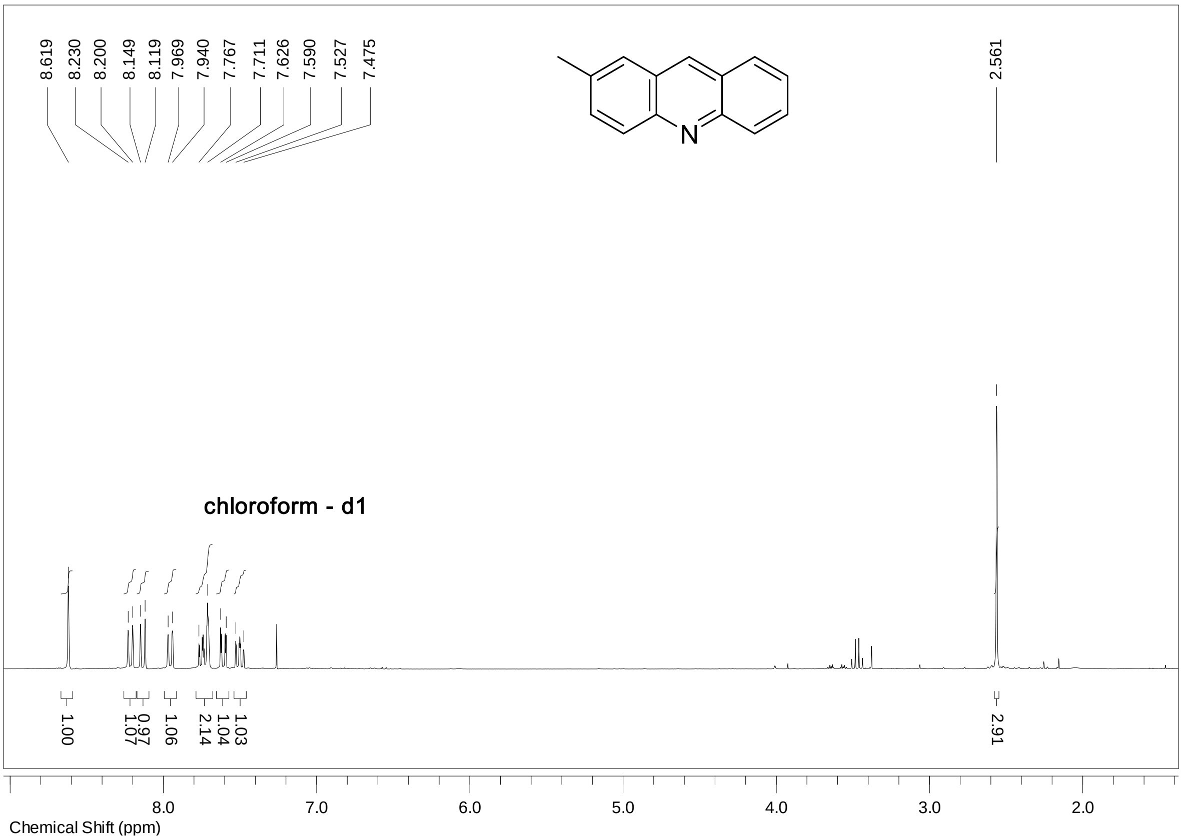

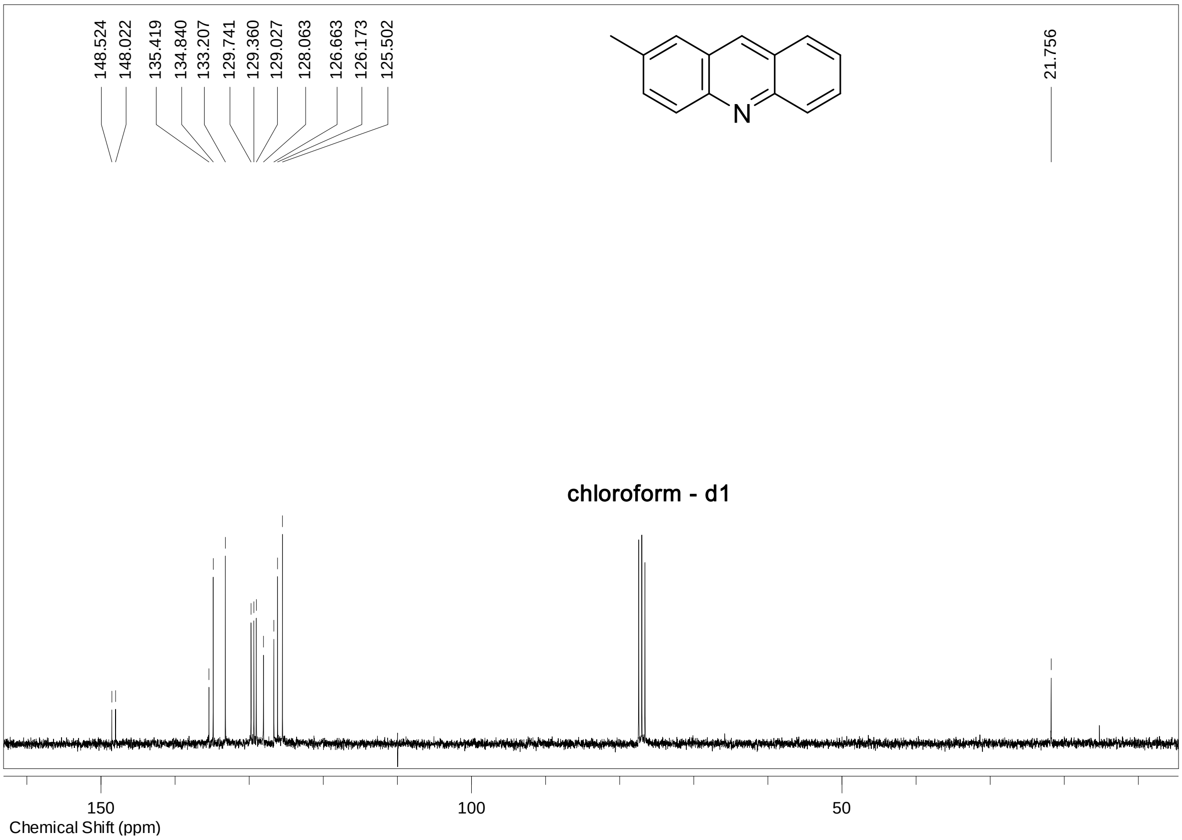

Supplementary Figure 25: NMR Spectra of compound 6c.

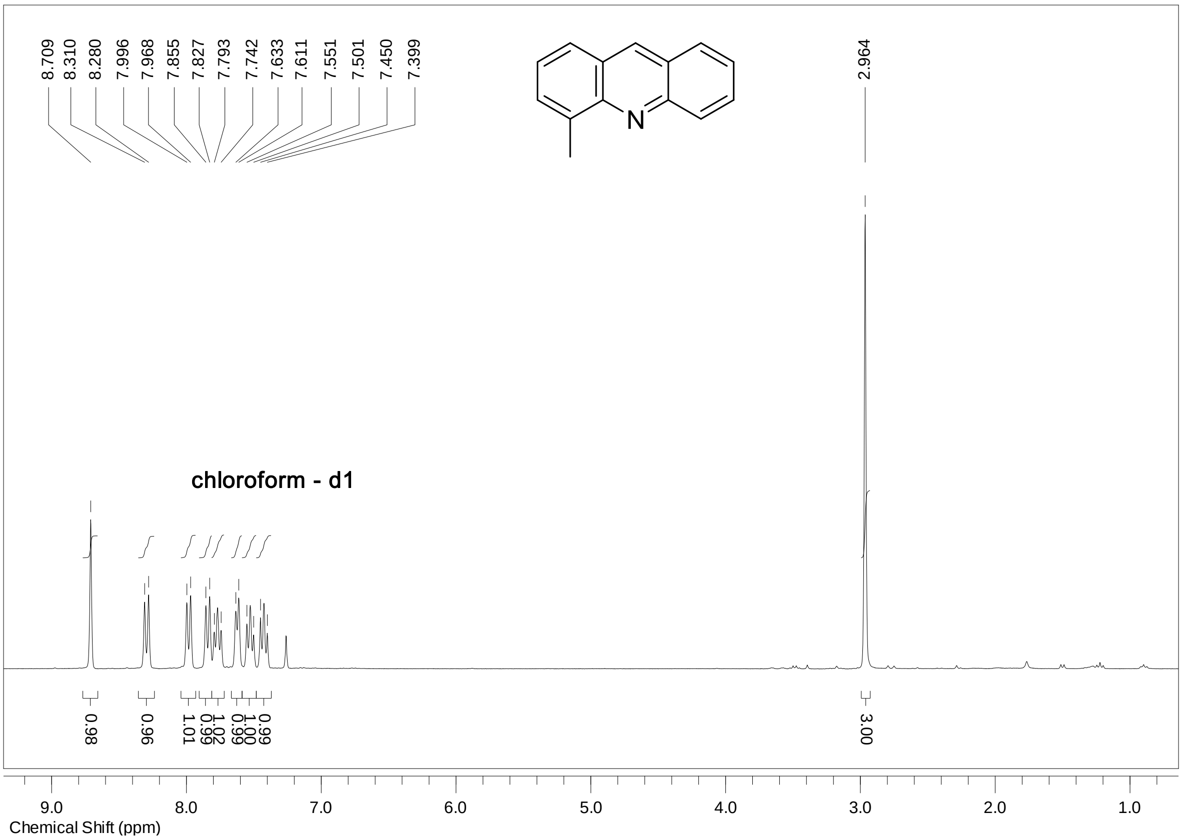

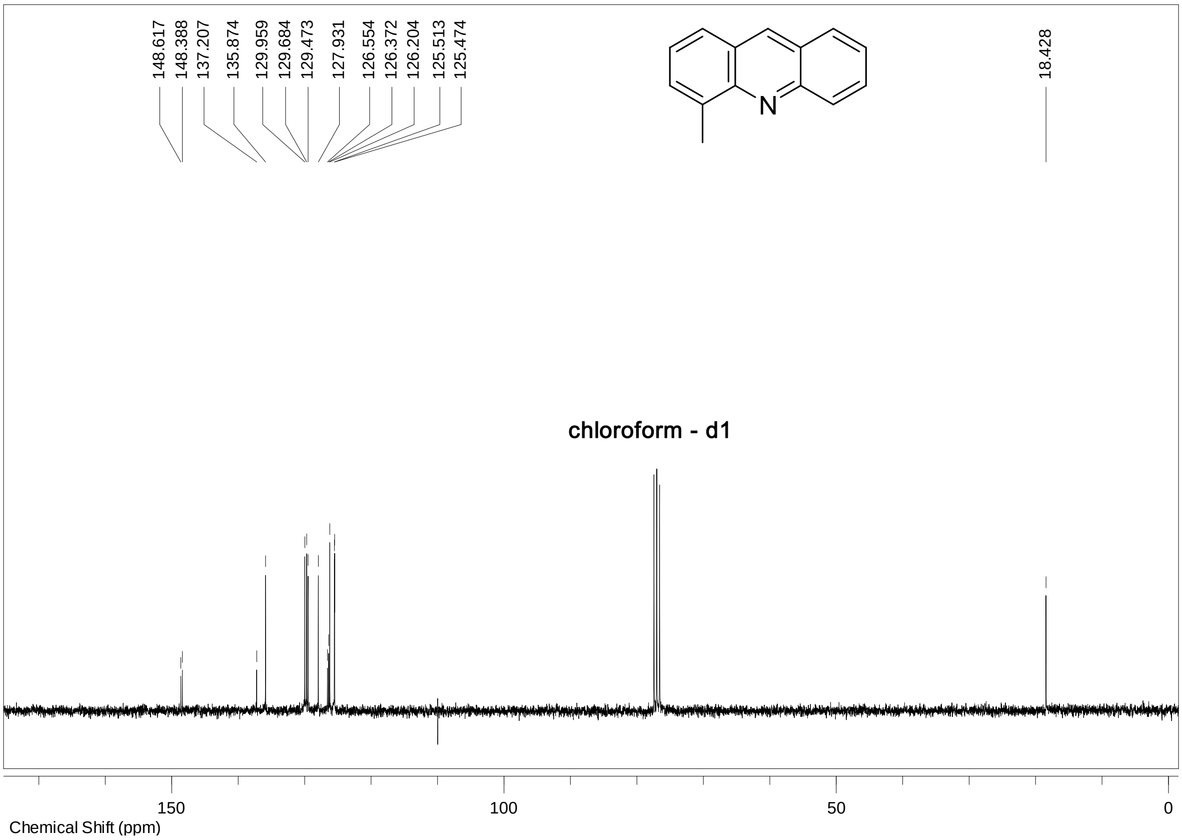

Supplementary Figure 26: NMR Spectra of compound 6d.

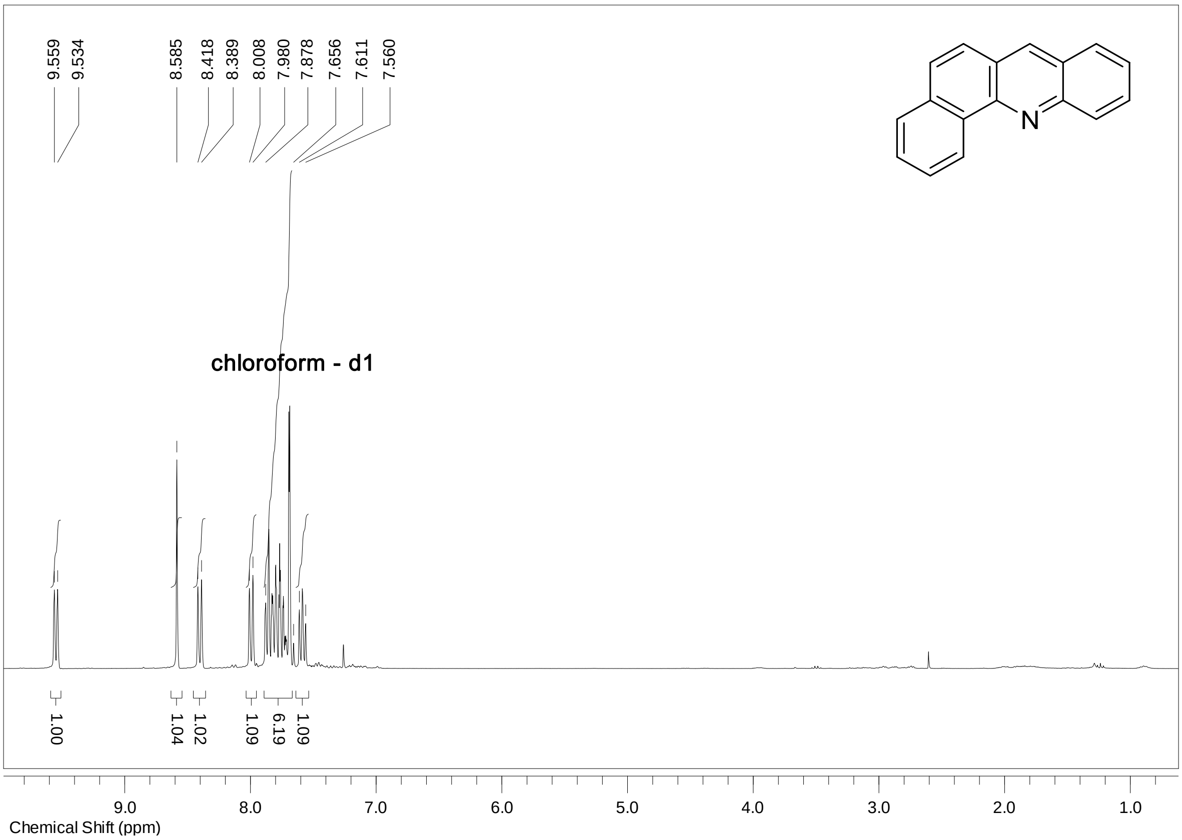

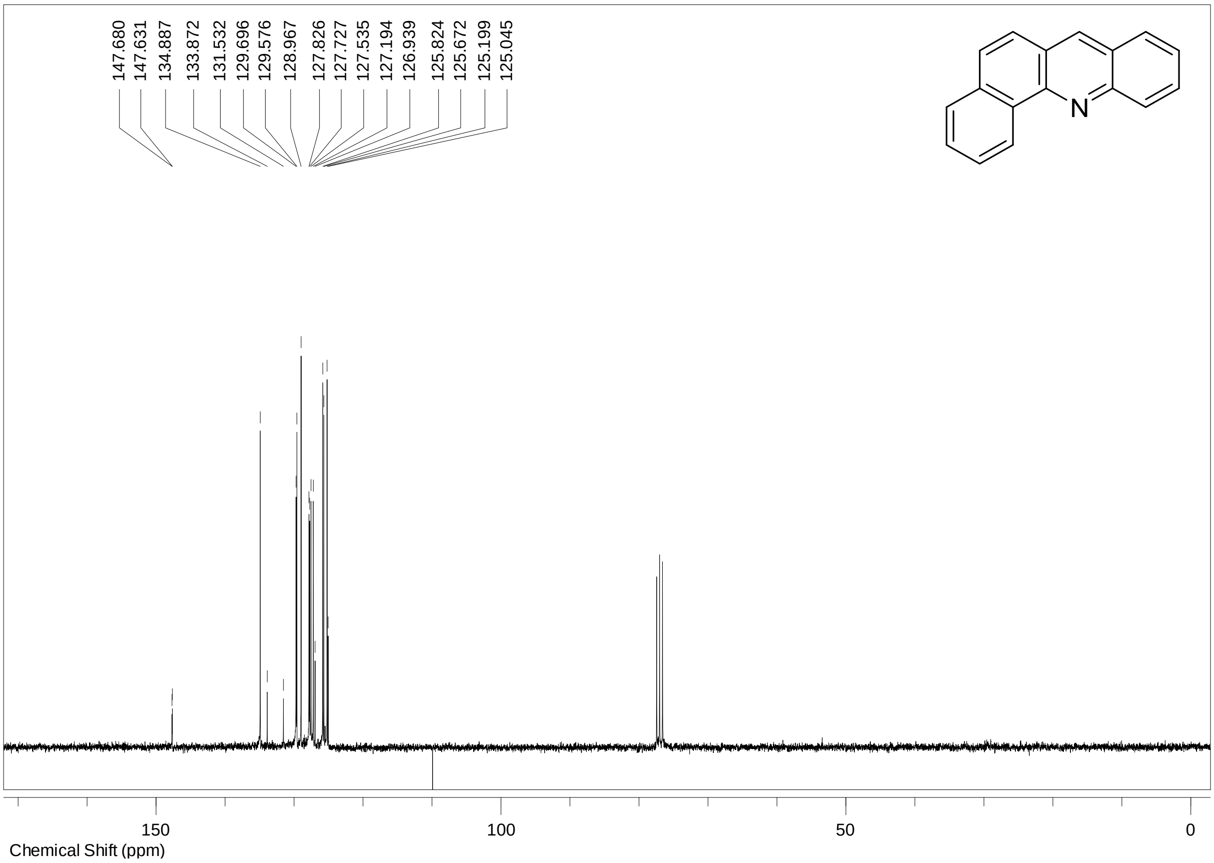

Supplementary Figure 27: NMR Spectra of compound 6e.

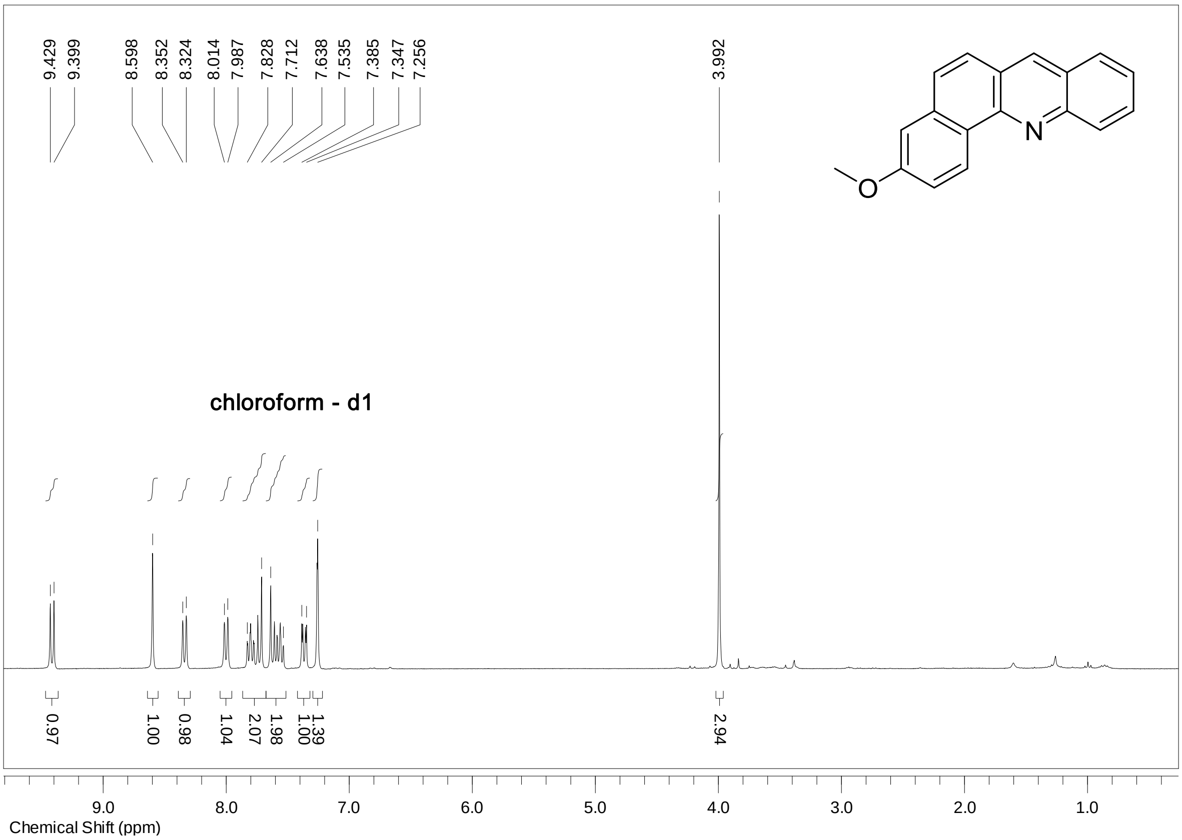

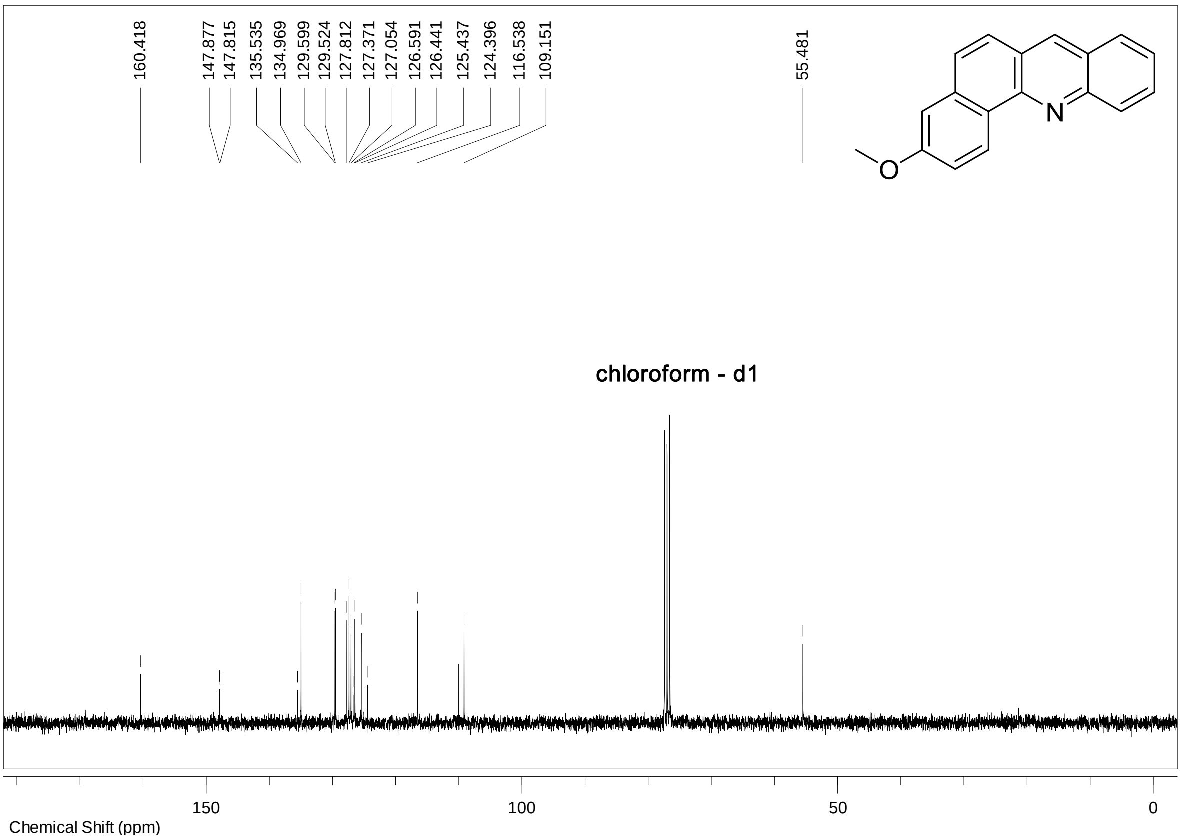

Supplementary Figure 28: NMR Spectra of compound 6f.

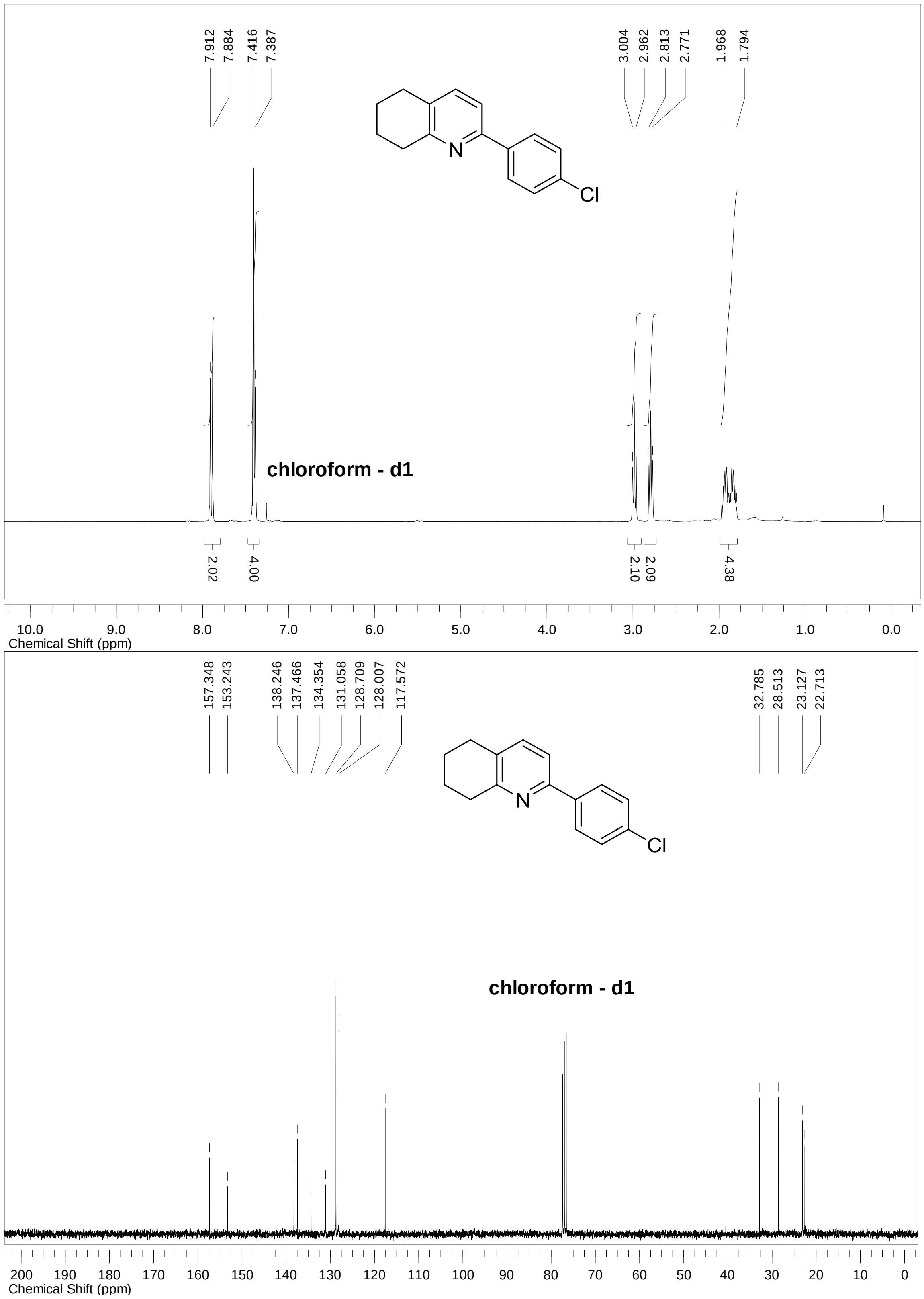

Supplementary Figure 29: NMR Spectra of compound 2f.

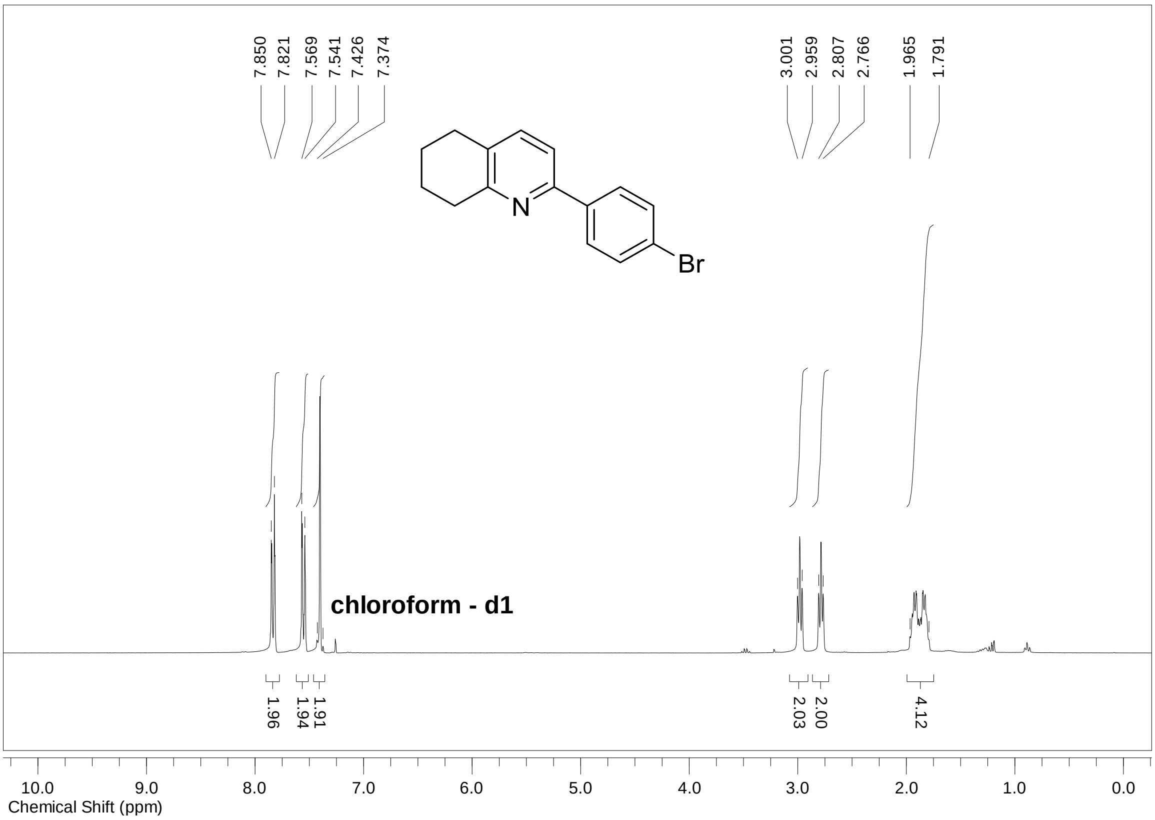

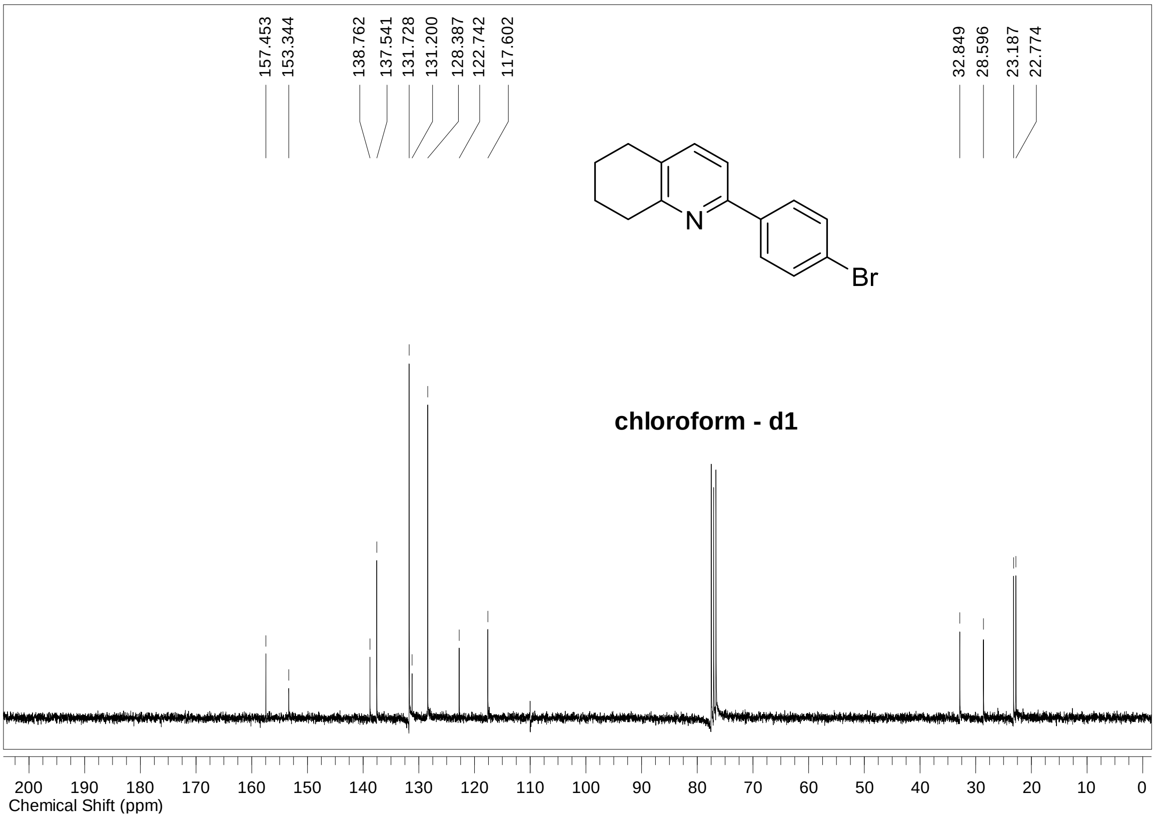

Supplementary Figure 30: NMR Spectra of compound 2g.

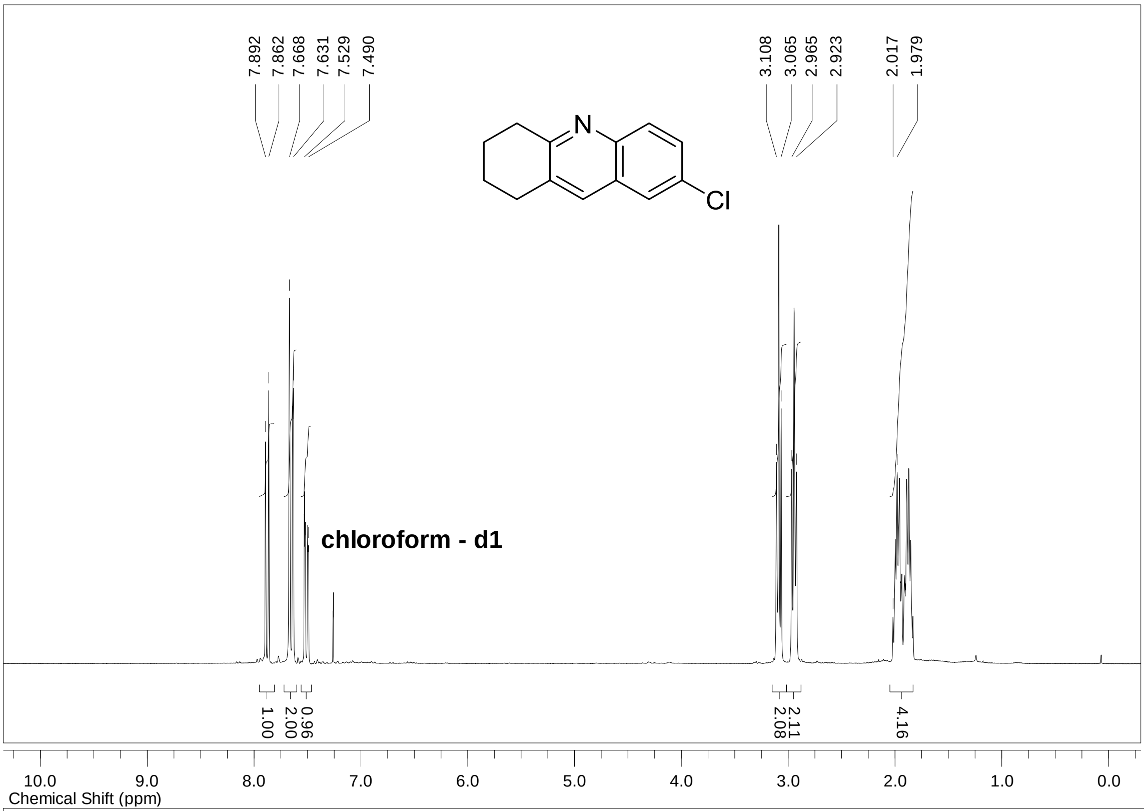

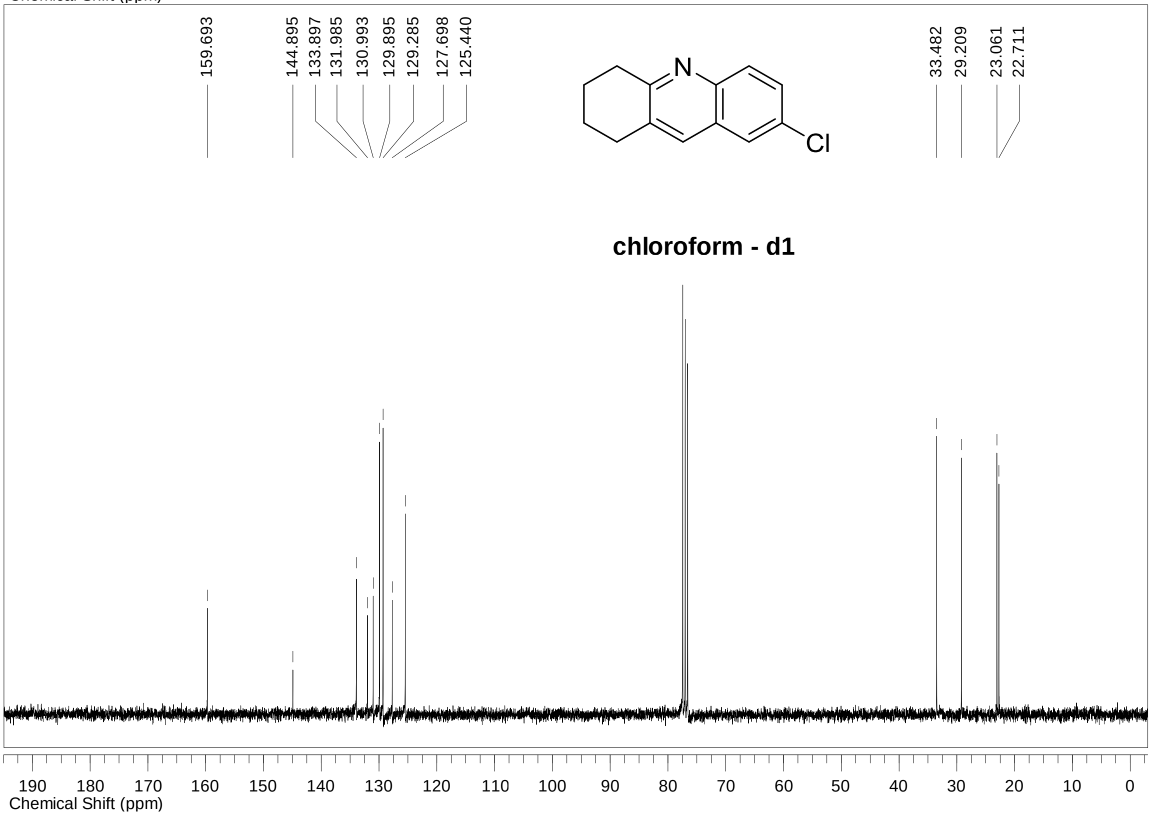

Supplementary Figure 31: NMR Spectra of compound 3e.

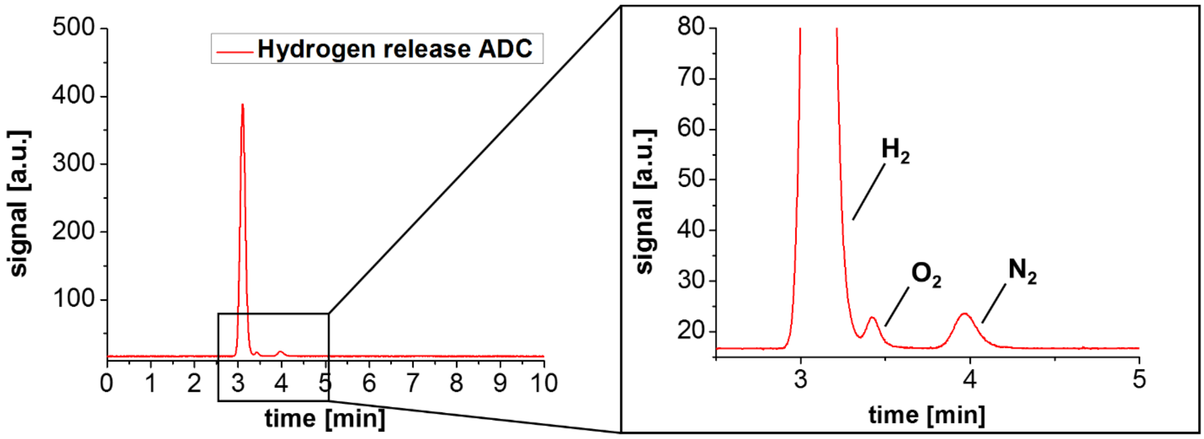

Supplementary Figure 32: GC analysis of the gas phase during ADC verifies the release of H_2. Small amounts of atmospheric nitrogen and oxygen are nearly unavoidable by manual injection.

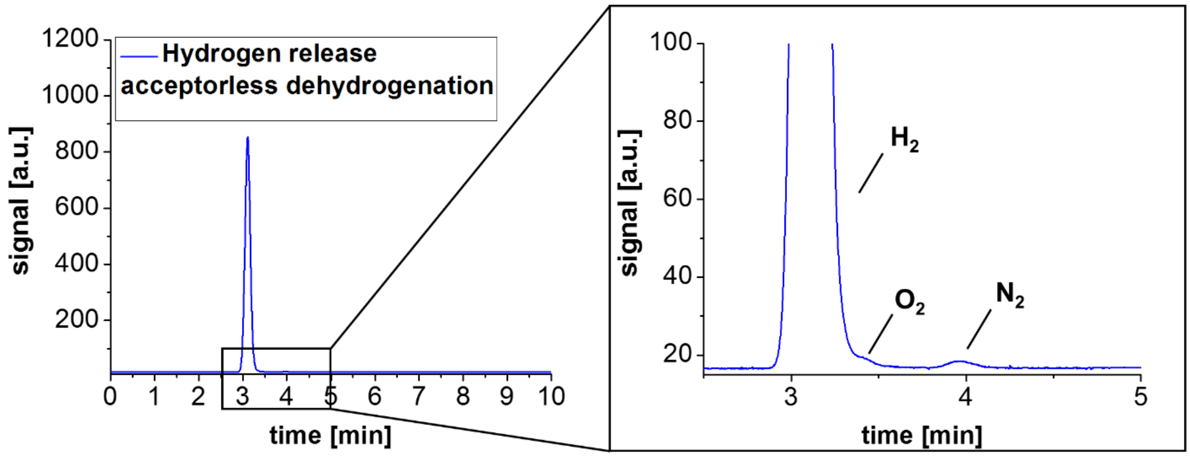

Supplementary Figure 33: GC analysis of the gas phase during acceptorless dehydrogenation verifies the release of H_2. Small amounts of atmospheric nitrogen and oxygen are nearly unavoidable by manual injection.

Supplementary Table 1: Comparison of Ru@SiCN to commercial catalysts in the hydrogenation of phenol[a)]

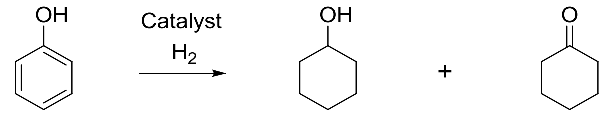

Catalyst	Yield[b)] [%]	Yield[b)] [%]
Ru@SiCN	80	0
Ru/C (5 %)	34	0
Ru/Al_2O_3 (5 %)	15	0
Pd/C (10 %)	3	3
Pd/SiO_2 (5 %)	0	0
Ir/C (1 %)	3	0
Ir/Al_2O_3 (1 %)	3	0
Ir/$CaCO_3$ (5 %)	12	0
Ir@SiCN	18	0
Pd@SiCN	10	22

a) 1 mmol substrate, 50 °C, p(H_2) = 3 bar, 0.03 mol% active metal referring to 5 mg Ru@SiCN, 1 mL H_2O, 5 h. b) Yields were determined by GC using cyclopentanol as internal standard.

Supplementary Table 2: Hydrogenation of phenolic compounds[a)]

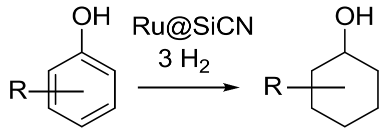

No.	R	Yield [%][b)]
**1**[c)]	none	100
**2**[d)]	none	97[e)]
**3**	1-methyl	100
**4**	1-ethyl	100
**5**	4-methyl	100
**6**	4-*tert*-butyl	100
**7**	3,5-dimethyl	92
**8**[f)]	2-amino	98

a) 1 mmol substrate, 50 °C, p(H_2) = 20 bar, 5 mg Ru@SiCN catalyst (0.03 mol% active metal), 1 mL water, 20 h. b) Yields determined by GC and GC-MS using dodecane as internal standard. c) 50 °C, 3 bar H_2 pressure, 24 h. d)100 mmol substrate, 50 °C, p(H_2) = 20 bar, 200 mg Ru@SiCN catalyst (0.01 mol% active metal), 10 mL water, 24 h. The reactor was pressured again to 20 bar after half of the reaction time. [e] Yield of isolated product. [f] 80 °C, p(H_2) = 50 bar, 24 h, 20 mg catalyst (0.12 mol% active Ru).

Supplementary Table 3: Temperature screening

T (oil bath) [°C]	Yield [%]
110	32
120	51
130	55
140	85
150	23

Reaction conditions: 150 mg (0.5 mol% active metal) Ir@SiCN, cyclohexanol (1268 µL, 12.0 mmol), 3-amino-3-(3,4-dimethoxyphenyl)propan-1-ol (635 mg, 3.0 mmol), 3 mL diglyme, KO^{t}Bu (673 mg, 6.0 mmol), 24 h. The reaction mixture was cooled to RT and water (3 mL) and dodecane as internal standard were added. The mixture was extracted with diethyl ether and a GC sample was taken.

Supplementary Table 4: Acceptorless dehydrogenation of **1a**

**Catalyst**	**Yield[a)] [%]** 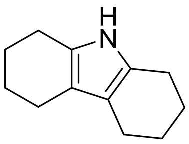	**Yield[a)] [%]** 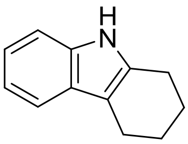	**Yield[a)] [%]** 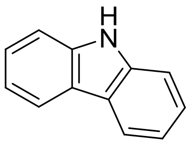
Pd@SiCN[b)]	0	8	92
Pd/C (10 %)[b)]	43	0	57
Pd/SiO_2 (5 %)[b)]	78	0	22
Ru@SiCN	17	74	9
Ru/C (5 %)	97	3	0
Ru/Al_2O_3 (5 %)	97	0	3
Ir@SiCN	96	1	2
Ir/C (1 %)	97	3	0
Ir/$CaCO_3$ (5 %)	100	0	0
Ir/Al_2O_3 (1 %)	100	0	0

Reaction conditions: Catalyst (0.18 mol% active metal), 0.5 mmol (88 mg) **2a**, 1 mL digylme, T (oil bath) = 180 °C (170 °C reaction temperature), Ar flow (4-6 mL/min), 24 h. a) Yields determined by GC. b) Reaction time: 5 h

Supplementary Table 5: Synthesis of carbazoles – Comparison between homogeneous and heterogeneous conditions.

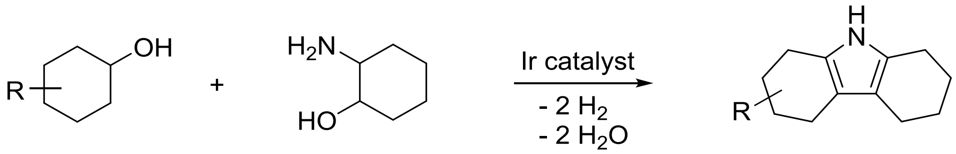

Product		Yield homogeneous cond. [%][b]	Yield heterogeneous cond. [%]	
		**105 °C**	**140 °C[c]**	**160 °C[b]**
**1a**	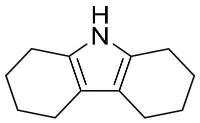	81	40	79
**1b**	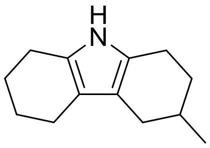	70	32	65
**1c**	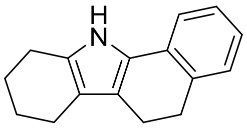	53	27	56

Reaction conditions: Homogeneous: 2.0 mL catalyst I (0.02 mmol, 0.01 M in thf), cyclohexanol compound (15.22 mmol), 2-aminocyclohexanol (7.61 mmol), 10 mL thf, 1.1 eq. KO^{t}Bu, 105 °C, 22 h; Heterogeneous: 150 mg Ir@SiCN (0.5 mol% active metal), cyclohexanol compound (12.0 mmol), 1,3-aminoalcohol (3.0 mmol), 3 mL diglyme, 2.0 eq. KO^{t}Bu, 140 °C / 160 °C, 24 h. [b] Yields of isolated products. [c] Yields were determined by GC and GC-MS using *n*-dodecane as internal standard.

Supplementary Table 6: Hydrogen release for the ADC coupling of cyclohexanol with 2-aminobenzyl alcohol.

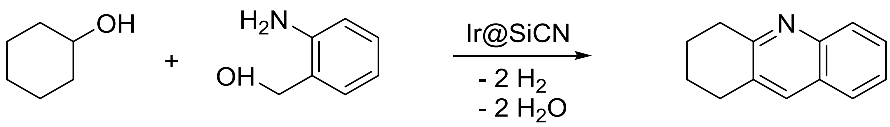

GC Yield H_2 [mmol][a]	Measured Volume H_2 [mmol]	Aberration [%]
4.21	4.52	7

Reaction conditions: 160 mg Ir@SiCN (0.8 mol% active metal), cyclohexanol (8.0 mmol), 2-aminobenzyl alcohol (2.0 mmol), 3 mL diglyme, 2.0 eq. KO^{t}Bu, 140 °C, 15 h. [a] Yields were determined by GC and GC-MS using *n*-dodecane as an internal standard. The calculated amount of hydrogen is based on the yield of 1,2,3,4-tetrahydoacridine, taking additional dehydrogenation of cyclohexanol to cyclo-hexanone into account.

Supplementary Table 7: Hydrogen release for the acceptorless dehydrogenation of 1,2,3,4-tetrahydroacridine.

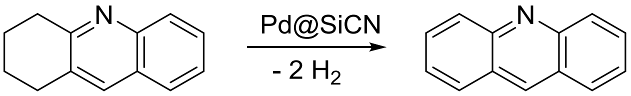

GC Yield H_2 [mmol][a]	Measured Volume H_2 [mmol]	Aberration [%]
3.84	3.52	8.3

Reaction conditions: 250 mg Pd@SiCN (0.23 mol% active metal), 2.0 mmol 1,2,3,4-tetrahydroacridine, 1 ml diglyme, 200 °C, 15 h. [a] Yields were determined by GC and GC-MS using *n*-dodecane as an internal standard. The calculated amount of hydrogen is based on the yield of 1,2,3,4-tetrahydroacridine.

Supplementary Methods

General Methods

Air- and moisture sensitive reactions were carried out under dry argon or nitrogen atmosphere using standard Schlenk or glove box techniques. Dry solvents were obtained from a solvent purification system (activated alumina cartridges) or purchased from Acros. Chemicals were purchased from commercial sources with purity over 95 % and used without further purification. Polysilazane “KiON HTT 1800” was purchased from Clariant Advanced Materials GmbH, Frankfurt (Germany) and used without further purification. NMR spectra were received using a Varian INOVA 300 (300 MHz for ^{1}H, 75 MHz for ^{13}C) at 296 K. Chemical shifts are reported in ppm relative to the residual solvent signal ($CDCl_3$: 7.26 ppm (^{1}H), 77.16 ppm (^{13}C); DMSO-d_6: 2.50 ppm (^{1}H), 39.51 ppm (^{13}C)), coupling constants (J) are reported in Hz. Elemental analysis was performed on an Elementar Vario El *III*. GC analyses were carried out on an Agilent 6890N Network GC system equipped with a HP-5 column (30 m x 0.32 mm x 0.25 µm). GC-MS analyses were carried out on an Agilent 7890A GC system equipped with a HP-5MS column (30 m x 0.32 mm x 0.25 µm) and a 5975C inert MSD detector. High resolution mass spectra (HRMS) were obtained from a Thermo Fisher Scientific Q-Exactive (Orbitrap) instrument in ESI+ mode.

Synthesis of the Ir Catalysts

The used iridium PN_5P-Ir-Pincer[1] and Ir@SiCN[2] catalysts were synthesized, characterized and used as reported.

Synthesis of the Pd@SiCN Catalyst

The Pd@SiCN catalyst was synthesized, characterized and used as reported.[3]

Synthesis of the Ru@SiCN Catalyst

The Pd@SiCN catalyst was synthesized, characterized and used as reported.[3]

Hydrogenation of Phenolic Compounds

Phenol could be hydrogenated at 50 °C and 3 bar H_2 pressure within 24 h using only 0.03 mol% active Ru. A comparison to other commercial catalysts with a reaction time of 5 h is given in Supplementary Table 1. The conditions and results of the hydrogenation of phenolic compounds can be found in Supplementary Table 2.

<u>Up-scaling:</u>

Into a reaction glass vial fitted with a magnetic stirring bar, 121 mmol (11.4 g) phenol, 200 mg Ru@SiCN catalyst (0.01 mol% ruthenium), 3 mL tetrahydrofuran and 2 mL water were added. The reaction vial was then placed in a 300 mL Parr autoclave and flushed three times with hydrogen. The autoclave was then pressured with 20 bar hydrogen and the reaction was stirred for 20 h at 50 °C. After half of the reaction time, the hydrogen pressure was again adjusted to 20 bar. After 20 h the hydrogen pressure was released and the sample was extracted five times with diethyl ether. After removal of the solvent under reduced pressure the crude product was obtained in > 95 % yield and analyzed by GC and GC-MS. The hydrogenation of 3,5-dimethylphenol required 80 °C on large scale for full conversion.

Synthesis of Carbazoles

ADC Coupling:

All carbazoles were prepared by modification of a literature method using the homogeneous iridium PN_5P-Ir-Pincer catalyst **I**.

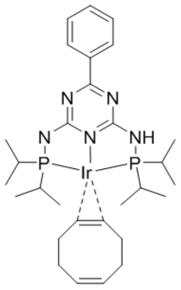

I

Typical Procedure:

In a glove box 2.0 mL catalyst **I** (0.02 mmol, 0.01 M in thf), cyclohexanol (15.22 mmol), 1,2-amino alcohol (7.61 mmol), 10 mL thf and KO^{t}Bu (8.37 mmol) were given in a pressure tube and sealed with a semi-permeable membrane. The tube was heated at 105 °C (oil bath temperature) for 22 h. After cooling to RT 3 mL water and dodecane as internal standard were added. The product was extracted with diethyl ether (2x) and purified by column chromatography or crystallization.

Acceptorless Dehydrogenation:

Typical Procedure:

In a 10 mL Schlenk tube 50 mg (0.18 mol% active metal) Pd@SiCN, 1.0 mmol substrate and 0.75 mL diglyme were evacuated and flushed with argon for three times. A slight argon flow of 4-6 mL/min was adjusted and the mixture was stirred for 20 h at 190 °C (oil bath temperature). After cooling to RT the catalyst was separated by centrifugation and washed with acetone two times. The organic phases were combined and the solvent was removed under reduced pressure at 60 °C giving the pure product. If required, further purification was achieved by either column chromatography or crystallization.

Comparison between heterogeneous and homogenous reaction conditions:

Regarding to carbazole synthesis, the homogeneous Ir pincer catalyst showed a higher activity than the reusable Ir@SiCN catalyst at 140 °C. However, the results could significantly be improved by an increase of the reaction temperature up to 160 °C (Supplementary Table 5).

Synthesis of 1a:

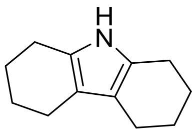

<u>**1a:** 2,3,4,5,6,7,8,9-octahydro-1*H*-carbazole</u>

2.0 mL catalyst **I** (0.02 mmol, 0.01 M in thf), cyclohexanol (1556 µL, 15.22 mmol), 2-aminocyclohexanol (875 mg, 7.61 mmol), 10 mL thf, KO^{t}Bu (943 mg, 8.40 mmol), 22 h at 105 °C. Purification by column chromatography 30:1 pentane : diethyl ether. Yield: 1.13 g = 6.42 mmol = 85 %. M($C_{12}H_{17}N$) = 175.27 gmol^{-1}.

^{1}H NMR (300 MHz, $CDCl_3$, 298 K): δ = 7.28 (s_br, 1H), 2.57-2.53 (m, 4H), 2.42-2.38 (m, 4H), 1.86-1.71 (m, 8H) ppm. ^{13}C NMR (75 MHz, $CDCl_3$, 298 K): δ = 124.9, 115.1, 23.7, 23.5, 22.8, 21.1 ppm. MS (EI, m/z): 174.9 (M^+).

elemental analysis (%) for $C_{12}H_{17}N$ calcd: C 82.23, H 9.78, N 7.99; found: C 82.08, H 9.71, N 7.09.

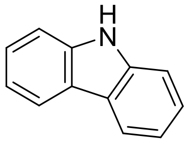

<u>**4a:** 9*H*-carbazole</u>

Yield: quantitative as light brown solid. M($C_{12}H_9N$) = 167.21 gmol^{-1}.

^{1}H NMR (300 MHz, $CDCl_3$, 298 K): δ = 8.13-8.10 (m, 2H), 8.10 (s_br, 1H), 7.46-7.44 (m, 4H), 7.31-7.24 (m, 2H) ppm. ^{13}C NMR (75 MHz, $CDCl_3$, 298 K): δ = 139.4, 125.8, 123.3, 120.3, 119.4, 110.5 ppm. MS (EI, m/z): 166.7 (M^+).

elemental analysis (%) for $C_{12}H_9N$ calcd: C 86.20, H 5.43, N 8.38; found: C 86.33, H 5.49, N 8.07.

The overall yield combining all three steps for product **4a** was 81 %.

Synthesis of 1b:

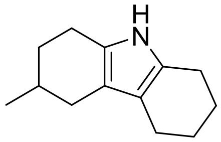

<u>**1b:** 3-methyl-2,3,4,5,6,7,8,9-octahydro-1*H*-carbazole</u>

2.0 mL Catalyst **I** (0.02 mmol, 0.01 M in thf), 4-methylcyclohexanol (1.74 g, 15.22 mmol), 2-aminocyclohexanol (875 mg, 7.61 mmol), 10 mL thf, KO^{t}Bu (943 mg, 8.40 mmol), 22 h at 105 °C. Purification by column chromatography 30:1 → 10 : 1 pentane : diethyl ether. Yield: 1.039 g = 5.49 mmol = 72 % as light yellow solid. M($C_{13}H_{19}N$) = 189.15 gmol^{-1}.

^{1}H NMR (300 MHz, $CDCl_3$, 298 K): δ = 7.28 (s_br, 1H), 2.63-2.53 (m, 5H), 2.43-2.38 (m, 2H), 2.05-1.96 (m, 1H), 1.89-1.72 (m, 6H), 1.56-1.40 (m, 1H), 1.08 (d, J = 6.6 Hz, 3H) ppm. ^{13}C NMR (75 MHz, $CDCl_3$, 298 K): δ = 125.2, 124.7, 115.2, 115.0, 31.9, 30.0, 29.8, 23.6, 23.5, 22.8, 22.6, 22.0, 21.1 ppm. MS (EI, m/z): 189.2 (M^+).

elemental analysis (%) for $C_{13}H_{19}N$ calcd: C 82.48, H 10.12, N 7.40; found: C 81.02, H 9.48, N 6.95.

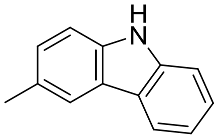

4b: 3-methyl-9*H*-carbazole

Yield: 97 % as colorless light brown solid. M($C_{13}H_{11}N$) = 181.09 $gmol^{-1}$.

^{1}H NMR (300 MHz, $CDCl_3$, 298 K): δ = 8.05 (d, J = 7.8 Hz, 1H), 7.93 (s_br, 1H), 7.88 (s, 1H), 7.41-7.40 (m, 2H), 7.32 (d, J = 8.4 Hz, 1H), 7.26-7.18 (m, 2H), 2.54 (s, 3H) ppm. ^{13}C NMR (75 MHz, $CDCl_3$, 298 K): δ = 141.6, 139.6, 128.3, 127.5, 126.0, 124.4, 124.1, 120.7, 120.7, 119.2, 111.4, 111.2, 21.7 ppm. MS (EI, m/z): 181.1 (M^+).

elemental analysis (%) for $C_{13}H_{11}N$ calcd: C 86.15, H 6.12, N 7.73; found: C 85.28, H 5.83, N 7.63.

The overall yield combining all three steps for product **4b** was 70 %.

Synthesis of 1c:

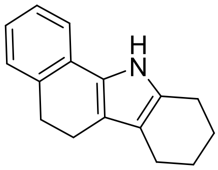

1c: 6,7,8,9,10,11-hexahydro-5*H*-benzo[*a*]carbazole

2.0 mL Catalyst **I** (0.02 mmol, 0.01 M in thf), 1,2,3,4-tetrahydronaphthalen-1-ol (2.23 g, 15.22 mmol), 2-aminocyclohexanol (875 mg, 7.61 mmol), 10 mL thf, KO^{t}Bu (943 mg, 8.40 mmol), 22 h at 105 °C. Purification by column chromatography 30:1 pentane : diethyl ether. Yield: 0.973 g = 4.36 mmol = 57 % as colorless solid. M($C_{16}H_{17}N$) = 223.14 $gmol^{-1}$.

^{1}H NMR (300 MHz, $CDCl_3$, 298 K): δ = 7.85 (s_br, 1H), 7.20-7.14 (m, 2H), 7.10-7.07 (m, 1H), 7.04-6.99 (m, 1H), 2.94 (t, J = 7.5 Hz, 2H), 2.68-2.62 (m, 4H), 2.49 (t, J = 7.5 Hz, 2H), 1.92-1.77 (m, 4H) ppm. ^{13}C NMR (75 MHz, $CDCl_3$, 298 K): δ = 134.4, 129.6, 128.2, 128.1, 126.3, 125.8, 124.2, 118.6, 117.6, 116.1, 29.9, 23.5, 23.4, 23.0, 21.2, 20.0 ppm. MS (EI, m/z): 223.2 (M^+).

elemental analysis (%) for $C_{16}H_{17}N$ calcd: C 86.05, H 7.67, N 6.27; found: C 85.55, H 7.62, N 5.95.

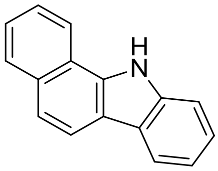

<u>**4c:** 11*H*-benzo[*a*]carbazole</u>

Yield: 96 % as light yellow solid. M($C_{16}H_{11}N$) = 217.09 $gmol^{-1}$.

^{1}H NMR (300 MHz, $CDCl_3$, 298 K): δ = 8.75 (s_br, 1H), 8.17-8.09 (m, 3H), 8.03 (d, *J* = 8.7 Hz, 1H), 7.68 (d, *J* = 8.7 Hz, 1H), 7.62-7.53 (m, 3H), 7.48-7.43 (m, 1H), 7.36-7.31 (m, 1H) ppm. ^{13}C NMR (75 MHz, $CDCl_3$, 298 K): δ = 138.4, 134.8, 132.4, 129.0, 125.5, 125.2, 124.8, 124.2, 121.1, 120.4, 120.2, 120.0, 119.9, 119.3, 118.4, 111.0 ppm. MS (EI, m/z): 217.1 (M^+).

elemental analysis (%) for $C_{16}H_{11}N$ calcd: C 88.45, H 5.10, N 6.45; found: C 88.54, H 5.26, N 6.40.

The overall yield combining all three steps for product **4c** was 53 %.

Synthesis of Tetrahydropyridines and Dehydrogenation to Quinolines

ADC Coupling:

The conditions of the tetrahydropyrrole synthesis were adopted. The best catalyst loading was found to be 0.5 mol% active metal. At the beginning, a small temperature screening was performed resulting in 140 °C as the best reaction temperature (Supplementary Table 3). All products except **2a** were synthesized using the heterogeneous Ir@SiCN catalyst.

General Procedure:

In a glove box 150 mg Ir@SiCN (0.5 mol% active metal), cyclohexanol (12.0 mmol), 1,3-aminoalcohol (3.0 mmol), 3 mL diglyme and KO*t*Bu (673 mg, 6.0 mmol) were added in a pressure tube and the tube was closed by a pressure equalization device. The mixture was stirred at 140 °C (oil bath temperature) for 24 h. After cooling to RT 3 mL water and dodecane as internal standard were added and the product was extracted by diethyl ether (2x). The products were purified either by column chromatography or crystallization.

Acceptorless Dehydrogenation

General Procedure

In a 10 mL Schlenk tube 50 mg (0.18 mol% active metal) Pd@SiCN, 1.0 mmol substrate and 0.75 mL diglyme were evacuated and flushed with argon for three times. A slight argon flow of 4-6 mL/min was adjusted and the mixture was stirred for 18 h at 200 °C (metal bath temperature). After cooling to RT the catalyst was separated by centrifugation and washed with acetone two times. The organic phases were combined and the solvent was removed under reduced pressure at 60 °C giving the pure product. If required, further purification can be achieved either by column chromatography or crystallization.

Synthesis of 2a:

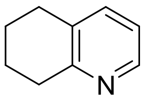

2a: 5,6,7,8-tetrahydroquinoline

1.5 mL Catalyst **I** (0.015 mmol, 0.01 M in thf), cyclohexanol (1268 µL, 12 mmol), 3-amino-1-propanol (228 mg, 3 mmol), 10 mL thf, NaO*t*Bu (317 mg, 3.3 mmol), 22 h at 110 °C. Purification by column chromatography 10:1 pentane : diethyl ether. Yield: 0.271 g = 2.04 mmol = 68 % as light colorless oil. M($C_9H_{11}N$) = 133.09 gmol^{-1}.

^1H NMR (300 MHz, $CDCl_3$, 298 K): δ = 8.31-8.29 (m, 1H), 7.31-7.28 (m, 1H), 6.99-6.95 (m, 1H), 2.91-2.86 (m, 2H), 2.74-2.70 (m, 2H), 1.90-1.72 (m, 4H) ppm. ^{13}C NMR (75 MHz, $CDCl_3$, 298 K): δ = 157.2, 146.5, 136.6, 132.1, 120.7, 32.3, 28.6, 22.9, 22.5 ppm. MS (EI, m/z): 133.1 (M^+).

elemental analysis (%) for $C_9H_{11}N$ calcd: C 81.16, H 8.32, N 10.52; found: C 81.57, H 8.64, N 10.85.

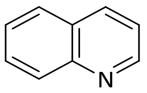

5a: quinoline
Yield: 92 % as yellow brown liquid by column chromatography with pentane : diethyl ether = 10 : 1. $M(C_9H_7N)$ = 129.16 $gmol^{-1}$.

^{1}H NMR (300 MHz, $CDCl_3$, 298 K): δ = 8.93-8.91 (m, 1H), 8.16-8.10 (m, 2H), 7.83-7.80 (m, 1H), 7.74-7.69 (m, 1H), 7.57-7.57 (m, 1H), 7.41-7.37 (m, 1H) ppm. ^{13}C NMR (75 MHz, $CDCl_3$, 298 K): δ = 150.4, 148.3, 136.0, 129.5, 129.4, 128.2, 127.7, 126.5, 121.0 ppm. MS (EI, m/z): 129.1 (M^+).

elemental analysis (%) for C_9H_7N calcd: C 83.69, H 5.46, N 10.84; found: C 83.10, H 5.48, N 10.83.

The overall yield combining all three steps for product **5a** was 58 %.

Synthesis of 2b:

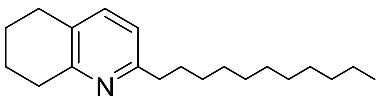

2b: 2-undecyl-5,6,7,8-tetrahydroquinoline
Purification by column chromatography 40:1→5:1 pentane: Et_2O;
Yield: 0.793 g = 0.276 mmol = 84 % as light yellow oil. $M(C_{20}H_{33}N)$ = 287.26 $gmol^{-1}$.

^{1}H NMR (300 MHz, $CDCl_3$, 298 K): δ = 7.24 (d, *J* = 7.8 Hz, 1H), 6.87 (d, *J* = 7.8 Hz, 1H), 2.88 (t, *J* = 6.3 Hz, 2H), 2.73-2.67 (m, 4H), 1.92-1.84 (m, 2H), 1.82-1.74 (m, 2H), 1.71-1.61 (m, 2H), 1.37-1.20 (m, 16H), 0.87 (t, *J* = 6.6 Hz, 3H) ppm. ^{13}C NMR (75 MHz, $CDCl_3$, 298 K): δ = 159.4, 156.4, 137.0, 129.0, 119.7, 38.3, 32.6, 31.9, 30.4, 29.6, 29.6, 29.6, 29.5, 29.5, 29.3, 28.4, 23.2, 22.8, 22.7, 14.1 ppm. MS (EI, m/z): 286.3 (M^+).

elemental analysis (%) for $C_{20}H_{33}N$ calcd: C 83.56, H 11.57, N 4.87; found: C: 82.60, H: 11.67, N: 4.13.

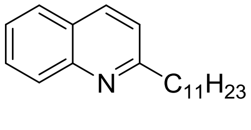

5b: 2-undecylquinoline
Dehydrogenation at 210 °C metal bath temperature for 36 h. Yield: 88 % as brown oil by column chromatography with pentane : Et_2O = 40 : 1. $M(C_{20}H_{29}N)$ = 283.23 $gmol^{-1}$.

^{1}H NMR (300 MHz, $CDCl_3$, 298 K): δ = 8.06 (d, *J* = 8.4 Hz, 1H), 8.06-8.04 (m, 1H), 7.79-7.76 (m, 1H), 7.71-7.65 (m, 1H), 7.50-7.45 (m, 1H), 7.30 (d, *J* = 8.4 Hz, 1H), 2.97 (t, *J* = 8.1 Hz, 2H), 1.86-1.76 (m, 2H), 1.36-1.19 (m, 16H), 0.88 (t, *J* = 6.3 Hz, 2H) ppm. ^{13}C NMR (75 MHz, $CDCl_3$, 298 K): δ = 163.1, 147.9, 136.2, 129.3, 128.8,

127.5, 126.7, 125.6, 121.4, 39.4, 31.9, 30.1, 29.7, 29.6, 29.6, 29.5, 29.4, 29.3, 22.7, 14.1 ppm. MS (EI, m/z): 283.2 (M^+).

elemental analysis (%) for $C_{20}H_{29}N$ calcd: C 84.75, H 10.31, N 4.94; found: C 84.25, H 10.20, N 4.44.

The overall yield combining all three steps for product **5b** was 72 %.

Synthesis of 2c:

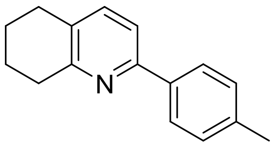

2c: 2-*p*-tolyl-5,6,7,8-tetrahydroquinoline
Purification by column chromatography 30:1→ 5:1 pentane: Et_2O;
Yield: 0.527 g = 2.36 mmol = 79 % as white solid. M($C_{16}H_{17}N$) = 223.24 gmol^{-1}.

^{1}H NMR (300 MHz, $CDCl_3$, 298 K): δ = 7.89 (d, *J* = 8.1 Hz, 2H), 7.41 (dd, *J* = 7.8 Hz, *J* = 9.9 Hz, 2H), 7.27 (d, *J* = 7.8 Hz, 2H), 3.05-3.00 (m, 2H), 2.82-2.78 (m, 2H), 2.42 (s, 3H), 1.99-1.91 (m, 2H), 1.89-1.81 (m, 2H) ppm. ^{13}C NMR (75 MHz, $CDCl_3$, 298 K): δ = 157.0, 154.5, 138.1, 137.2, 137.0, 130.2, 129.2, 126.6, 117.5, 32.8, 28.4, 23.2, 22.8, 21.1 ppm. MS (EI, m/z): 223.2 (M^+).

elemental analysis (%) for $C_{16}H_{17}N$ calcd: C 86.05, H 7.67, N 6.27; found: C 55.99, H 7.94, N 5.97.

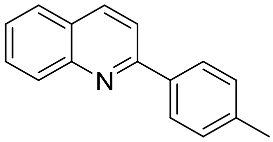

5c: 2-*p*-tolylquinoline
Yield: 94 % as light brown solid by recrystallization from diethyl ether. M($C_{16}H_{13}N$) = 219.28 gmol^{-1}.

^{1}H NMR (300 MHz, $CDCl_3$, 298 K): δ = 8.21-8.16 (m, 2H), 8.09 (d, *J* = 8.1 Hz, 2H), 7.88-7.80 (m, 2H), 7.75-7.70 (m, 1H), 7.54-7.49 (m, 1H), 3.34 (d, *J* = 8.4 Hz, 2H), 2.44 (s, 3H) ppm. ^{13}C NMR (75 MHz, $CDCl_3$, 298 K): δ = 157.33, 148.3, 139.4, 136.9, 136.8, 136.7, 136.7, 129.7, 129.6, 127.4, 127.1, 126.1, 118.9, 21.4 ppm. MS (EI, m/z): 219.2 (M^+).

elemental analysis (%) for $C_{16}H_{13}N$ calcd: C 87.64, H 5.98, N 6.39; found: C 87.30, H 6.12, N 6.36.

The overall yield combining all three steps for product **5c** was 72 %.

Synthesis of 2d:

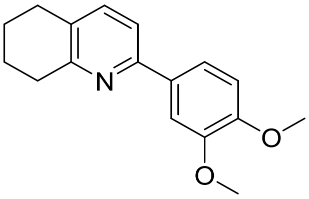

<u>**2d:** 2-(3,4-dimethoxyphenyl)-5,6,7,8-tetrahydroquinoline</u>
Purification by column chromatography 5:1→1:1 pentane: Et_2O;
Yield: 0.687 g = 2.52 mmol = 85 % as colorless solid. M($C_{17}H_{19}NO_2$) = 269.14 $gmol^{-1}$.

^1H NMR (300 MHz, $CDCl_3$, 298 K): δ = 7.59-7.58 (m, 1H), 7.46-7.30 (m, 3H), 6.90-6.87 (m, 1H), 3.95 (s, 3H), 3.87 (s, 3H), 2.98-2.94 (m, 2H), 2.75-2.71 (m, 2H), 1.92-1.72 (m, 4H) ppm. ^{13}C NMR (75 MHz, $CDCl_3$, 298 K): δ = 156.8, 154.0, 149.3, 148.9, 137.1, 132.7, 129.9, 119.0, 117.1, 110.8, 109.7, 55.7, 55.7, 32.6, 28.3, 23.0, 22.6 ppm. MS (EI, m/z): 269.1 (M^+).

elemental analysis (%) for $C_{17}H_{19}NO_2$ calcd: C 75.81, H 7.11, N 5.20; found: C 75.41, H 7.37, N 4.91.

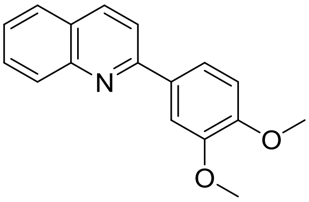

<u>**5d:** 2-(3,4-dimethoxyphenyl)quinoline</u>
Yield: 93 % as colorless solid by recrystallization from diethyl ether. M($C_{17}H_{15}NO_2$) = 265.11 $gmol^{-1}$.

^1H NMR (300 MHz, $CDCl_3$, 298 K): δ = 8.18-8.15 (m, 2H), 7.89-7.78 (m, 3H), 7.74-7.64 (m, 2H), 7.52-7.45 (m, 1H), 7.00-6.97 (m, 2H), 4.05 (s, 3H), 3.95 (s, 3H) ppm. ^{13}C NMR (75 MHz, $CDCl_3$, 298 K): δ = 156.7, 150.3, 149.3, 148.1, 136.6, 132.5, 129.5, 129.4, 127.4, 126.9, 125.9, 120.2, 118.5, 111.0, 110.3, 56.0, 56.0 ppm. MS (EI, m/z): 265.1 (M^+).

elemental analysis (%) for $C_{17}H_{15}NO_2$ calcd: C 76.96, H 5.70, N 5.28; found: C 76.82, H 5.85, N 5.14.

The overall yield combining all three steps for product **5d** was 77 %.

Synthesis of 2e:

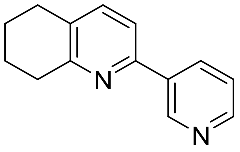

<u>**2e:** 2-(pyridin-3-yl)-5,6,7,8-tetrahydroquinoline</u>
Purification by column chromatography 1:1 pentane: Et_2O → pure Et_2O;
Yield: 0.417 g = 1.98 mmol = 66 % as yellow oil. M($C_{14}H_{14}N_2$) = 210.27 $gmol^{-1}$.

^{1}H NMR (300 MHz, $CDCl_3$, 298 K): δ = 9.14-9.13 (m, 1H), 8.62-8.59 (m, 1H), 8.30-8.26 (m, 1H), 7.46-7.45 (m, 2H), 7.38-7.34 (m, 1H), 3.02-2.97 (m, 2H), 2.84-2.79 (m, 2H), 1.98-1.81 (m, 4H) ppm. ^{13}C NMR (75 MHz, $CDCl_3$, 298 K): δ = 157.8, 151.8, 149.4, 148.2, 137.6, 135.3, 134.2, 131.7, 123.5, 117.9, 32.8, 28.6, 23.1, 22.7 ppm. MS (EI, m/z): 210.2 (M^+).

elemental analysis (%) for $C_{14}H_{14}N_2$ calcd: C 79.97, H 6.71, N 13.32; found: C 79.06, H 6.79, N 12.44.

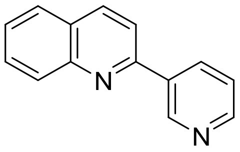

<u>**5e:** 2-(pyridin-3-yl)quinoline</u>
Yield: 97 % as red-brown oil. M($C_{14}H_{10}N_2$) = 206.24 $gmol^{-1}$.

^{1}H NMR (300 MHz, $CDCl_3$, 298 K): δ = 9.36-9.35 (m, 1H), 8.71-8.69 (m, 1H), 8.53-8.49 (m, 1H), 8.25 (d, *J* = 8.7 Hz, 1H), 8.17 (d, *J* = 8.7 Hz, 1H), 7.89-7.83 (m, 1H), 7.86 (s, 1H), 7.78-7.72 (m, 1H), 7.58-7.53 (m, 1H), 7.47-7.43 (m, 1H) ppm. ^{13}C NMR (75 MHz, $CDCl_3$, 298 K): δ = 154.6, 150.2, 148.8, 148.3, 137.1, 135.1, 134.9, 129.9, 129.7, 127.5, 127.3, 126.7, 123.6, 118.5 ppm. MS (EI, m/z): 206.2 (M^+).

HRMS (ESI): calcd. for $C_{14}H_{11}N_2$ $[M+H]^+$: 207.09168; found: 207.09170.

The overall yield combining all three steps for product **5e** was 62 %.

Synthesis of 2f:

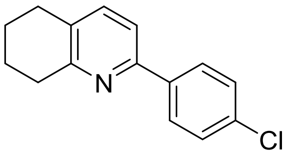

<u>**2f:** 2-(4-chlorophenyl)-5,6,7,8-tetrahydroquinoline</u>
Purification by column chromatography 20:1→5:1 pentane:Et_2O;
Yield: 0.497 g = 2.04 mmol = 68 % as light yellow solid.
M($C_{15}H_{14}NCl$) = 243.73 $gmol^{-1}$

^{1}H NMR (300 MHz, $CDCl_3$, 298 K): δ = 7.91-7.88 (m, 2H), 7.42-7.39 (m, 4H), 3.00-2.96 (m, 2H), 2.81-2.77 (m, 2H), 1.97-1.79 (m, 4H) ppm. ^{13}C NMR (75 MHz, $CDCl_3$, 298 K): δ = 157.3, 153.2, 138.2, 137.5, 134.4, 131.1, 128.7, 128.0, 117.6, 32.8, 28.5, 23.1, 22.7 ppm.

elemental analysis (%) for $C_{15}H_{14}ClN$ calcd: C 73.92, H 5.79, N 5.75; found: C 74.11, H 5.37, N 5.91.

Synthesis of 2g:

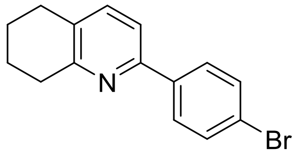

2f: 2-(4-bromophenyl)-5,6,7,8-tetrahydroquinoline

Purification by column chromatography 20:1→5:1 pentane:Et_2O;
Yield: 0.527 g = 1.83 mmol = 61 % as a white solid.
M($C_{15}H_{14}NBr$) = 288.19 $gmol^{-1}$

^{1}H NMR (300 MHz, $CDCl_3$, 298 K): δ = 7.85-7.82 (m, 2H), 7.57-7.54 (m, 2H), 7.43-7.37 (m, 2H), 3.00-2.96 (m, 2H), 2.81-2.77 (m, 2H), 1.97-1.79 (m, 4H) ppm. ^{13}C NMR (75 MHz, $CDCl_3$, 298 K): δ = 157.5, 153.3, 138.8, 137.5, 131.7, 131.2, 128.4, 122.7, 117.6, 32.9, 28.6, 23.2, 22.8 ppm.

elemental analysis (%) for $C_{15}H_{14}NBr$ calcd: C 62.52, H 4.90, N 4.86; found: C 62.35, H 5.31, N 5.13.

Synthesis of Tetrahydroacridines and Dehydrogenation to Acridines

ADC Coupling:

General Procedure:

In a glove box 150 mg Ir@SiCN (0.5 mol% active metal), cyclohexanol (12.0 mmol), 1,3-aminoalcohol (3.0 mmol), 3 mL diglyme and KO*t*Bu (673 mg, 6.0 mmol) were added in a pressure tube and the tube was closed by a pressure equalization device. The mixture was stirred at 140 °C (oil bath temperature) for 24 h. After cooling to RT 3 mL water and dodecane as internal standard were added and the product was extracted by diethyl ether (2x). The products were purified either by column chromatography or crystallization.

Acceptorless Dehydrogenation

General Procedure

In a 10 mL Schlenk tube 50 mg (0.18 mol% active metal) Pd@SiCN, 1.0 mmol substrate and 0.75 mL diglyme were evacuated and flushed with argon for three times. A slight argon flow of 4-6 mL/min was adjusted and the mixture was stirred for 18 h at 200 °C (oil bath temperature). After cooling to RT the catalyst was separated by centrifugation and washed with acetone two times. The organic phases were combined and the solvent was removed under reduced pressure at 60 °C giving the pure product. If required, further purification can be achieved either by column chromatography or crystallization.

Synthesis of 3a:

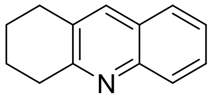

3a: 1,2,3,4-tetrahydroacridine
Purification by column chromatography 1:20 → 1:5 pentane : Et_2O. Yield: 0.457 g = 2.50 mmol = 83 % as yellow solid. M($C_{13}H_{13}N$) = 183.10 gmol^{-1}.

^{1}H NMR (300 MHz, $CDCl_3$, 298 K): δ = 7.99-7.96 (m, 1H), 7.80 (s, 1H), 7.71-7.68 (m, 1H), 7.63-7.57 (m, 1H), 7.45-7.40 (m, 1H), 3.15-3.11 (m, 2H), 3.00-2.96 (m, 2H), 2.04-1.96 (m, 2H), 1.93-1.85 (m, 2H) ppm. ^{13}C NMR (75 MHz, $CDCl_3$, 298 K): δ = 159.3, 146.6, 134.9, 130.9, 128.4, 128.3, 127.2, 126.9, 125.5, 33.6, 29.3, 23.2, 22.9 ppm. MS (EI, m/z): 183.1 (M^+).

elemental analysis (%) for $C_{13}H_{13}N$ calcd: C 85.21, H 7.15, N 7.64; found: C 84.39, H 7.18, N 7.61.

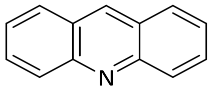

6a: acridine

Yield: 97 % as yellow solid. M($C_{13}H_9N$) = 179.22 gmol^{-1}.

^{1}H NMR (300 MHz, $CDCl_3$, 298 K): δ = 8.76 (s, 1H), 8.26-8.23 (m, 2H), 8.01-7.98 (m, 2H), 7.81-7.76 (m, 2H), 7.56-7.51 (m, 2H) ppm. ^{13}C NMR (75 MHz, $CDCl_3$, 298 K): δ = 149.0, 136.0, 130.3, 129.4, 128.2, 126.6, 125.7 ppm. MS (EI, m/z): 179.1 (M^+).

elemental analysis (%) for $C_{13}H_9N$ calcd: C 87.12, H 5.06, N 7.82; found: C 87.03, H 5.26, N 7.70.

The overall yield combining all three steps for product **6a** was 79 %.

Synthesis of 3b:

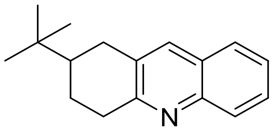

3b: 2-*tert*-butyl-1,2,3,4-tetrahydroacridine

Purification by column chromatography 20:1 → 3:1 pentane : Et_2O. Yield: 0.66 g = 2.76 mmol = 92 % as light yellow solid. M($C_{17}H_{21}N$) = 239.17 gmol^{-1}.

^{1}H NMR (300 MHz, $CDCl_3$, 298 K): δ = 7.99-7.96 (m, 1H), 7.82 (s, 1H), 7.71-7.68 (m, 1H), 7.63-7.57 (m, 1H), 7.45-7.40 (m, 1H), 3.32-3.23 (m, 1H), 3.11-3.01 (m, 2H), 2.77-2.72 (m, 1H), 2.20-2.12 (m, 1H), 1.65-1.52 (m, 2H), 1.00 (s, 9H) ppm. ^{13}C NMR (75 MHz, $CDCl_3$, 298 K): δ = 159.4, 146.6, 135.2, 131.2, 128.4, 128.3, 127.2, 126.8, 125.5, 44.6, 34.4, 32.6, 30.8, 27.3, 24.6 ppm. MS (EI, m/z): 239.1 (M^+).

elemental analysis (%) for $C_{17}H_{21}N$ calcd: C 85.30, H 8.84, N 5.85; found: C 85.07, H 8.87, N 5.77.

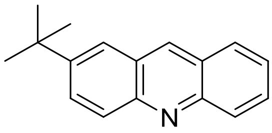

6b: 2-*tert*-butylacridine

Yield: 98 % as colorless solid by recrystallization from pentane/diethyl ether. M($C_{17}H_{17}N$) = 235.14 gmol^{-1}.

^{1}H NMR (300 MHz, $CDCl_3$, 298 K): δ = 8.71 (s, 1H), 8.24-8.17 (m, 2H), 7.99-7.97 (m, 1H), 7.92-7.86 (m, 2H), 7.78-7.72 (m, 1H), 7.54-7.49 (m, 1H), 1.46 (s, 9H) ppm. ^{13}C NMR (75 MHz, $CDCl_3$, 298 K): δ = 148.7, 148.2, 148.1, 135.7, 130.0, 129.8, 129.4, 128.9, 128.1, 126.7, 126.4, 125.4, 125.3, 35.0, 30.9 ppm. MS (EI, m/z): 235.1 (M^+).

HRMS (ESI): calcd. for $C_{17}H_{18}N$ $[M+H]^+$: 236.14337; found: 236.14338.

The overall yield combining all three steps for product **6b** was 87 %.

Synthesis of 3c:

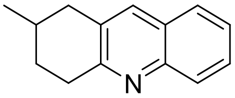

<u>**3c:** 2-methyl-1,2,3,4-tetrahydroacridine</u>
Oil bath temperature: 135 °C. Purification by column chromatography 5:1 → 1:1 pentane : Et_2O. Yield: 0.416 g = 2.11 mmol = 70 % as yellow solid. M($C_{14}H_{15}N$) = 197.12 $gmol^{-1}$.

^{1}H NMR (300 MHz, $CDCl_3$, 298 K): δ = 7.98-7.95 (m, 1H), 7.73 (s, 1H), 7.68-7.65 (m, 1H), 7.61-7.56 (m, 1H), 7.43-7.38 (m, 1H), 3.26-3.17 (m, 1H), 3.14-2.95 (m, 2H), 2.60-2.51 (m, 1H), 2.10-1.90 (m, 2H), 1.65-1.51 (m, 1H), 1.10 (d, *J* = 6.3 Hz, 3H) ppm. ^{13}C NMR (75 MHz, $CDCl_3$, 298 K): δ = 158.9, 146.6, 134.8, 130.5, 128.4, 128.2, 127.1, 126.8, 125.4, 37.7, 33.1, 31.4, 29.0, 21.6 ppm. MS (EI, m/z): 197.1 (M^+).

HRMS (ESI): calcd. for $C_{14}H_{16}N$ $[M+H]^+$: 198.12772; found: 198.12773.

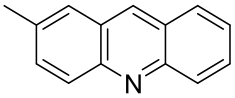

<u>**6c:** 2-methylacridine</u>
Yield: 96 % as yellow-orange solid; purification by column chromatography with diethyl ether as eluent. M($C_{14}H_{11}N$) = 193.09 $gmol^{-1}$.

^{1}H NMR (300 MHz, $CDCl_3$, 298 K): δ = 8.62 (s, 1H), 8.22 (d, *J* = 9.0 Hz, 1H), 8.13 (d, *J* = 9.0 Hz, 1H), 7.97-7.94 (m, 1H), 7.77-7.71 (m, 2H), 7.63-7.59 (m, 1H), 7.53-7.48 (m, 1H), 2.56 (s, 3H) ppm. ^{13}C NMR (75 MHz, $CDCl_3$, 298 K): δ = 148.5, 148.0, 135.4, 134.8, 133.2, 129.7, 129.4, 129.0, 128.1, 126.7, 126.2, 125.5, 21.8 ppm. MS (EI, m/z): 193.1 (M^+).

HRMS (ESI): calcd. for $C_{14}H_{12}N$ $[M+H]^+$: 194.09642; found: 194.09643.

The overall yield combining all three steps for product **6c** was 68 %.

Synthesis of 3d:

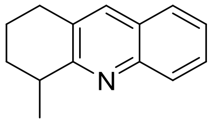

<u>**3d:** 4-methyl-1,2,3,4-tetrahydroacridine</u>
Oil bath temperature: 135 °C. Purification by column chromatography 3:1 pentane : Et_2O. Yield: 0.427 g = 2.17 mmol = 72 % as yellow oil. M($C_{14}H_{15}N$) = 197.12 $gmol^{-1}$.

^{1}H NMR (300 MHz, $CDCl_3$, 298 K): δ = 8.02-7.96 (m, 1H), 7.75-7.53 (m, 3H), 7.43-7.35 (m, 1H), 3.23-3.17 (m, 1H), 2.93-2.92 (m, 2H), 2.17-2.08 (m, 1H), 2.01-1.87 (m, 1H), 1.84-1.67 (m, 2H), 1.50-1.48 (m, 3H) ppm. ^{13}C NMR (75 MHz, $CDCl_3$, 298 K): δ = 162.9, 146.7, 134.6, 130.3, 128.4, 128.1, 126.9, 126.6, 125.3, 36.5, 31.2, 29.6, 21.5, 20.1 ppm. (EI, m/z): 197.1 (M^+).

HRMS (ESI): calcd. for $C_{14}H_{16}N$ $[M+H]^+$: 198.12380; found: 198.12773.

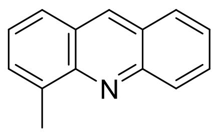

6d: 4-methylacridine
Yield: 93 % as yellow solid; purification by crystallization from pentane/diethyl ether. M($C_{14}H_{11}N$) = 193.09 gmol^{-1}.

^{1}H NMR (300 MHz, $CDCl_3$, 298 K): δ = 8.71 (s, 1H), 8.31-8.28 (m, 1H), 7.98 (d, *J* = 8.4 Hz, 1H), 7.84 (d, *J* = 8.4 Hz, 1H), 7.79-7.74 (m, 1H), 7.63-7.61 (m, 1H), 7.55-7.50 (m, 1H), 7.45-7.40 (m, 1H), 2.96 (s, 3H) ppm. ^{13}C NMR (75 MHz, $CDCl_3$, 298 K): δ = 148.6, 148.4, 137.2, 135.9, 130.0, 129.7, 129.5, 127.9, 126.6, 126.4, 126.2, 125.5, 125.5, 18.4 ppm. MS (EI, m/z): 194.1 (M^+).

elemental analysis (%) for $C_{14}H_{11}N$ calcd: C 87.01, H 5.74, N7.25; found: C 86.45, H 6.04, N 7.10. HRMS (ESI): calcd. for $C_{14}H_{12}N$ $[M+H]^+$: 194.09250; found: 194.09596.

The overall yield combining all three steps for product **6d** was 65 %.

Synthesis of 3f:

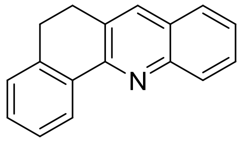

3e: 5,6-dihydrobenzo[*c*]acridine:
Purification by column chromatography 1:20 pentane : Et_2O; Yield: 0.643 g = 2.78 mmol = 93 % as colorless solid. M($C_{17}H_{13}N$) = 231.10 gmol^{-1}.

^{1}H NMR (300 MHz, $CDCl_3$, 298 K): δ = 8.61-8.58 (m, 1H), 8.16-8.13 (m, 1H), 7.92 (s, 1H), 7.76-7.73 (m, 1H), 7.68-7.63 (m, 1H), 7.50-7.35 (m, 3H), 7.30-7.27 (m, 1H), 3.16-3.11 (m, 2H), 3.04-2.99 (m, 2H) ppm. ^{13}C NMR (75 MHz, $CDCl_3$, 298 K): δ = 153.4, 147.6, 139.4, 134.7, 133.6, 130.5, 129.6, 129.4, 128.6, 127.9, 127.8, 127.3, 129.9, 126.0, 125.9, 28.8, 28.4 ppm. MS (EI, m/z): 230.2 (M^+).

elemental analysis (%) for $C_{17}H_{13}N$ calcd: C 88.28, H 5.67, N 6.06; found: C 88.13, H 5.90, N 5.71.

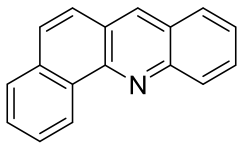

6e: benzo[*c*]acridine
Yield: 98 % as colorless solid by recrystallization from pentane/diethyl ether. M($C_{17}H_{11}N$) = 229.09 gmol^{-1}.

^{1}H NMR (300 MHz, $CDCl_3$, 298 K): δ = 9.56-9.53 (m, 1H), 8.59 (s, 1H), 8.40 (d, *J* = 8.7 Hz,1H), 7.99 (d, *J* = 8.4 Hz,1H), 7.88-7.66 (m, 6H), 7.61-7.56 (m, 1H) ppm.

^{13}C NMR (75 MHz, $CDCl_3$, 298 K): δ = 147.7, 147.6, 134.9, 133.9, 131.5, 129.7, 129.6, 129.0, 127.8, 127.7, 127.5, 127.2, 126.9, 125.8, 125.7, 125.2, 125.0 ppm. MS (EI, m/z): 229.1 (M^+).

elemental analysis (%) for $C_{17}H_{11}N$ calcd: C 89.06, H 4.84, N 6.11; found: C 88.66, H 5.02, N 5.93.

The overall yield combining all three steps for product **6e** was 88 %.

Synthesis of 3g:

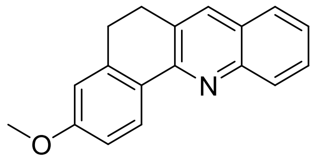

2H-4f: 3-methoxy-5,6-dihydrobenzo[*c*]acridine
Purification by column chromatography 5:1 → 1:2 pentane : Et_2O. Yield: 0.724 g = 2.77 mmol = 92 % as light yellow solid. M($C_{18}H_{15}NO$) = 261.12 gmol^{-1}.

^{1}H NMR (300 MHz, $CDCl_3$, 298 K): δ = 8.57 (d, *J* = 8.7 Hz, 1H), 8.14 (d, *J* = 8.7 Hz, 1H), 7.80 (s, 1H), 7.70-7.62 (m, 2H), 7.46-7.41 (m, 1H), 7.00-6.96 (m, 1H), 6.79-6.78 (m, 1H), 3.85 (s, 3H), 3.08-3.03 (m, 2H), 2.96-2.92 (m, 2H) ppm. ^{13}C NMR (75 MHz, $CDCl_3$, 298 K): δ = 160.7, 153.2, 147.5, 141.1, 133.3, 129.8, 129.0, 128.4, 127.6, 127.4, 126.8, 126.8, 125.4, 112.9, 112.7, 55.1, 28.7, 28.6 ppm. MS (EI, m/z): 261.1 (M^+).

elemental analysis (%) for $C_{18}H_{15}NO$ calcd: C 82.73, H 5.79, N 5.36; found: C 82.83, H 6.00, N 5.18.

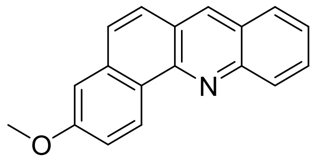

6f: 3-methoxybenzo[*c*]acridine
Yield: 98 % as colorless solid. M($C_{18}H_{13}NO$) = 259.10 gmol^{-1}.

^{1}H NMR (300 MHz, $CDCl_3$, 298 K): δ = 9.41 (d, *J* = 9.0 Hz, 1H), 8.60 (s, 1H), 8.34 (d, *J* = 8.4 Hz, 1H), 8.00 (d, *J* = 8.1 Hz, 1H), 7.83-7.71 (m, 2H), 7.64-7.54 (m, 2H), 7.39-7.35 (m, 1H), 7.26 (s, 1H), 3.99 (s, 3H) ppm. ^{13}C NMR (75 MHz, $CDCl_3$, 298 K): δ = 160.4, 147.9, 147.8, 135.5, 135.0, 129.6, 129.5, 127.8, 127.4, 127.1, 126.6, 126.4, 125.4, 124.4, 116.5, 109.2, 55.5 ppm. MS (EI, m/z): 259.1 (M^+).

elemental analysis (%) for $C_{18}H_{13}NO$ calcd: C 83.37, H 5.05, N 5.40; found: C: 82.78, H 5.05, N 5.21.

The overall yield combining all three steps for product **6f** was 79 %.

Synthesis of 3e:

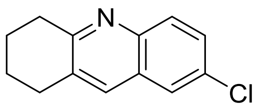

<u>**3e:** 7-chloro-1,2,3,4-tetrahydroacridine</u>
Purification by column chromatography 15:1→2:1 pentane:Et_2O;
Yield: 0.470 g = 2.16 mmol = 72 % as a light yellow solid.
M($C_{13}H_{12}ClN$) = 217.70 $gmol^{-1}$.

^{1}H NMR (300 MHz, $CDCl_3$, 298 K): δ = 7.89-7.86 (m, 1H), 7.67-7.63 (m, 2H), 7.53-7.49 (m, 1H), 3.11-3.07 (m, 2H), 2.97-2.92 (m, 2H), 2.02-1.98. ^{13}C NMR (75 MHz, $CDCl_3$, 298 K): δ = 159.7, 144.9, 133.9, 132.0, 131.0, 129.9, 129.3, 127.7, 125.4, 33.5, 29.2, 23.1, 22.7 ppm.

elemental analysis (%) for $C_{13}H_{12}ClN$ calcd: C 71.73, H 5.56, N 6.43; found: C 71.29, H 5.37, N 6.31.

Comparison to Commercial Catalysts

All available heterogeneous catalysts were applied in the acceptorless dehydrogenation of 2,3,4,5,6,7,8,9-octahydro-1*H*-carbazole **1a** (Supplementary Table 4).

In a 10 mL Schlenk tube 0.5 mmol **1a** were solved in 1.0 mL diglyme and the catalyst (0.18 mol% active metal) was added. The reaction mixture was evacuated and flushed with argon for three times and a slight argon flow of 4-6 mL/min was adjusted. The Schlenk tube was placed in a pre-heated oil bath at 180 °C for 6-24 h. After cooling to RT in an argon atmosphere, dodecane as internal standard was added and a sample for GC and GC-MS analysis was taken.

Hydrogen release experiments

The yield of H_2 was quantified for the ADC coupling of cyclohexanol and 2-aminobenzyl alcohol, as well as for the following dehydrogenation of 1,2,3,4-tetrahydroacridine.

ADC:

In a glove box 160 mg Ir@SiCN (0.8 mol% active metal), cyclohexanol (8.0 mmol), 2-aminobenzyl alcohol (2.0 mmol), 3 mL diglyme and 2 eq. KO^{t}Bu (448 mg, 4.0 mmol) were added in a 25 ml Schlenk tube. The tube was connected to a reflux condenser, linked to a water column. The mixture was heated up to 140 °C and after a short equilibration time the released hydrogen was collected. The results are in good agreement with the theoretically expected values (Supplementary Table 6). To ensure a clean and selective dehydrogenation process a GC analysis of the gas phase was accomplished (Supplementary Figure 32).

Acceptorless Dehydrogenation:

In a glove box 2.0 mmol 1,2,3,4-tetrahydroacridine, 1 ml diglyme and 250 mg Pd@SiCN were given in a 25 mL Schlenk tube. The tube was connected to a reflux condenser, linked to a water column. The mixture was heated up to 200 °C and after a short equilibration time the released hydrogen was collected. The results are in good agreement with the theoretically expected values (Supplementary Table 7). To ensure a clean and selective dehydrogenation process a GC analysis of the gas phase was accomplished (Supplementary Figure 33).

Supplementary References

1. Michlik, S. & Kempe, R. Regioselectively functionalized pyridines from sustainable resources. *Angew. Chem. Int. Ed.* **52**, 6326–6329 (2013).

2. Forberg, D. et al. The synthesis of pyrroles via acceptorless dehydrogenative condensation of secondary alcohols and 1,2-amino alcohols mediated by a robust and reusable catalyst based on nanometer-sized iridium particles. *Catal. Sci. Technol.* **4**, 4188–4192 (2014).

3. Forberg, D. et al. Single-catalyst high-weight% hydrogen storage in an N-heterocycle synthesized from lignin hydrogenolysis products and ammonia. *Nat. Commun.* **7**, 13201 (2014).
